# Insights into the mechanisms of NH_3_ inhibition on Cu-CHA SCR catalysts

**DOI:** 10.1038/s41467-026-72879-7

**Published:** 2026-07-28

**Authors:** Dhruba J. Deka, Mingyu Wan, Garam Lee, Eric Walter, Fanglin Che, Kenneth G. Rappe, János Szanyi, Yong Wang

**Affiliations:** 1 Institute for Integrated Catalysis, Pacific Northwest National Laboratory, Richland, WA USA; 2 Department of Chemical Engineering, University of Massachusetts Lowell, Lowell, MA USA; 3 Department of Chemical Engineering, Worcester Polytechnic Institute, Worcester, MA USA; 4 The Gene and Linda Voiland School of Chemical Engineering and Bioengineering, Washington State University, Pullman, WA USA

**Keywords:** Heterogeneous catalysis, Catalytic mechanisms, Chemical engineering

## Abstract

Ammonia (NH_3_) inhibition during the selective catalytic reduction (SCR) of nitrogen oxides (NO_x_) over Cu-exchanged chabazite (Cu-CHA) catalysts limits low-temperature performance, yet its molecular origin remains unclear. Here we show that excess NH_3_ selectively suppresses the oxidation half-cycle of the SCR reaction while leaving the reduction half-cycle largely unaffected. By combining kinetic measurements, operando electron paramagnetic resonance spectroscopy, and density functional theory calculations, we identify hindered mobility of Cu^+^ ions as the key factor. Specifically, NH_3_ coordination increases the energy barrier for Cu^+^ diffusion, preventing the formation of reactive Cu^2+^-oxo dimer intermediates required for efficient oxidation. Spectroscopic measurements further reveal that the extent of inhibition depends strongly on temperature and copper loading. These insights provide a mechanistic basis for mitigating NH_3_ inhibition, suggesting that improved catalyst design and optimized operating conditions can enhance low-temperature SCR performance in practical emission control systems.

## Abstract (web summary)

Selective catalytic reduction (SCR) of nitrogen oxides (NOₓ) using ammonia (NH₃) over small-pore Cu-zeolites is a key technology for mitigating NOₓ emissions from lean-burn engines. Here, the authors clarify the atomic-scale mechanism responsible for ammonia inhibition during NOₓ SCR on Cu-CHA catalysts.

## Introduction

The selective catalytic reduction (SCR) of nitrogen oxides (NO_x_) with ammonia (NH_3_) over small-pore Cu-zeolites (e.g., Cu-CHA, Cu-AEI, Cu-LTA) is a cornerstone technology for controlling NO_x_ emissions from lean-burn engines^1,2^. These materials offer high activity, selectivity, and hydrothermal stability, rendering them indispensable in advanced emissions control systems. In Cu-CHA, the primary active sites are isolated Cu^II^ ions that exist in two distinct coordination environments: ZCu^II^OH, where Cu^II^ is coordinated to a single framework Al and a hydroxyl group, and Z₂Cu^II^, where Cu^II^ is stabilized by paired Al sites in the framework^3–5^.

The NH_3_-SCR reaction (4NO + 4NH_3_ + O_2_ → 4N_2_ + 6H_2_O) proceeds via a redox mechanism involving a reduction half-cycle (RHC) and an oxidation half-cycle (OHC), with isolated Cu sites cycling between Cu^II^ and Cu^I^ states^4–8^. The RHC is achieved through the reduction of Cu^II^ to Cu^I^ by NO and NH_3_, forming linear [Cu^I^(NH_3_)_2_]^+^ complexes. The solvation effect of NH_3_ on Cu^I^ sites decreases their electrostatic interaction with the negatively charged zeolite framework, granting mobility to these complexes^9–11^. The OHC involves the activation of O_2_ on Cu^I^ sites. Since O_2_ activation requires four electrons, and Cu^I^ can provide only one, the process is more facile when two Cu^I^ sites are involved which leads to the formation of O_2_-bridged dimeric Cu^II^ species^4,5,12–14^. The mobility of [Cu^I^(NH_3_)_2_]^+^ complexes is crucial for the OHC as they need to diffuse between cages to co-locate with another [Cu^I^(NH_3_)_2_]^+^ to form Cu dimers. This quasi-homogeneous process dominates below ~250 °C, while at higher temperatures Cu(I) ions become immobilized, and SCR proceeds via more heterogeneous pathways involving mononuclear Cu-nitrate/nitrite intermediates^4,15–18^.

The mobility of Cu ions, especially at low temperatures, is influenced by several physicochemical properties, including zeolite topology, Si/Al ratio, Al siting, Cu loading, and Cu speciation^5,9,19–21^. For example, a higher Si/Al ratio reduces Brønsted acidity and Cu exchange capacity, impacting both reactivity and diffusion^3^. Spatial inhomogeneities in Al distribution can further constrain Cu mobility due to the difficulty of intercage migration between Al-rich and Al-lean regions^9^. Cu loading also strongly affects Cu-Cu pairing probability and, thus, OHC kinetics: higher loadings promote dimer formation, while lower loadings reduce Cu ion encounters and amplify diffusion limitations^20^.

Reaction environment and solvation further modulate Cu mobility. NH_3_ and H_2_O solvation lowers mobility restrictions at low temperatures, but excess NH_3_ can occupy Brønsted sites and obstruct critical diffusion pathways (e.g., 8-membered ring windows), thereby impeding Cu transport^3^. Excess reactants such as NO, H_2_O, and O_2_ can further increase the energy required for [Cu^I^(NH_3_)_2_]^+^ diffusion.^22^. Additionally, certain reactants, such as NO_2_ or O_2_, can destabilize mobile Cu^I^ species or oxidize them into framework-bound Cu^II^, further limiting mobility and redox cycling^23^. These observations suggest that SCR performance is highly sensitive to local reaction conditions and the coordination environment of Cu sites.

Reactant-induced inhibition is a broader catalytic phenomenon wherein excess reactants or intermediates impair activity by forming inactive complexes, competing for adsorption, or hindering diffusion^23–25^. Understanding these mechanisms and their impacts is essential for optimizing catalytic processes. The inhibition of SCR by NH_3_ is well-documented, particularly on vanadium-based catalysts, where NH_3_ competes with NO for active V_2_O_5_ sites^26^. NH_3_ inhibition of fast SCR reactions on Fe-zeolites, on the other hand, results from NH_3_ interacting with surface nitrates, preventing their reaction with NO^27^. However, despite growing interest in Cu-CHA, NH_3_ inhibition in standard SCR has received limited attention. Most existing insights derive from theoretical predictions, indirect experimental evidence, or demonstration under transient conditions^19,22,28–31^. The lack of fundamental studies can be attributed to the focus on high Cu-loadings, where inhibition is not easily observable in steady-state tests. In real-world applications, Cu sites deactivate with time, reducing effective loading and thereby exposing the catalyst to NH_3_ inhibition, especially under onboard excess dosing strategies aimed at avoiding parasitic NH_3_ oxidation losses. Furthermore, NH_3_ internal combustion engine exhaust may contain up to 2–3 times as much NH_3_ as NO_x_, raising the potential for NH_3_ inhibition even on relatively fresh Cu-CHA catalysts.

In this study, we uncover the atomic-level mechanisms of NH_3_ inhibition on Cu-CHA catalysts by integrating steady-state kinetic measurements, RHC/OHC-specific modeling, operando EPR spectroscopy, and DFT calculations. We demonstrate that NH_3_ inhibition emerges above a Cu- and temperature-dependent critical NH_3_:NO ratio, where excess NH₃ impairs Cu ion mobility and selectively suppresses OHC kinetics. These findings provide actionable insights into catalyst design and operating conditions for mitigating NH_3_ inhibition in next-generation SCR systems.

## Results and discussion

### SCR kinetics and NH_3_-inhibition

We first demonstrate the practical relevance of NH_3_ inhibition on Cu-CHA during SCR by examining the performance of commercial degreened (DG) and field-aged (FA) samples. Sample details are provided in Supplementary Table 1. As shown in Supplementary Fig. 1a, both DG and FA samples showed a steady increase in NOₓ conversion at 200 °C with rising ammonia-to-NO_x_ ratios (ANR), up to 1.0 for DG and 0.5 for FA. Beyond these points, conversion declines with further increases in ANR, indicating NH_3_-induced inhibition above a critical ANR that depends on catalyst state. The manifestation of inhibition at 200 °C, a temperature relevant to real-world exhaust conditions, highlights the operational significance of this phenomenon. Transient light-off and light-down tests (Supplementary Fig. 1b) further corroborate the presence of NH_3_ inhibition.

To investigate its mechanistic origin, we synthesized a series of model Cu-CHA catalysts with Cu loadings of 0.48 wt%, 1.38 wt%, and 2.48 wt%, referred to as Cu-0.5, Cu-1.4, and Cu-2.5, respectively. The elemental compositions are detailed in Supplementary Table 1. All samples were prepared from the same parent zeolite support (Si/Al ratio of ~12). Recent studies have shown that differences in the spatial distribution of [AlO_2_]^–^ centers in the zeolite framework can influence Cu ion mobility and SCR activity^19^. Thus, by using a single parent support, we eliminate the confounding effects of both Cu and Al ions to ensure that our observations are primarily influenced by the state of the Cu ions.

**Fig. 1:**
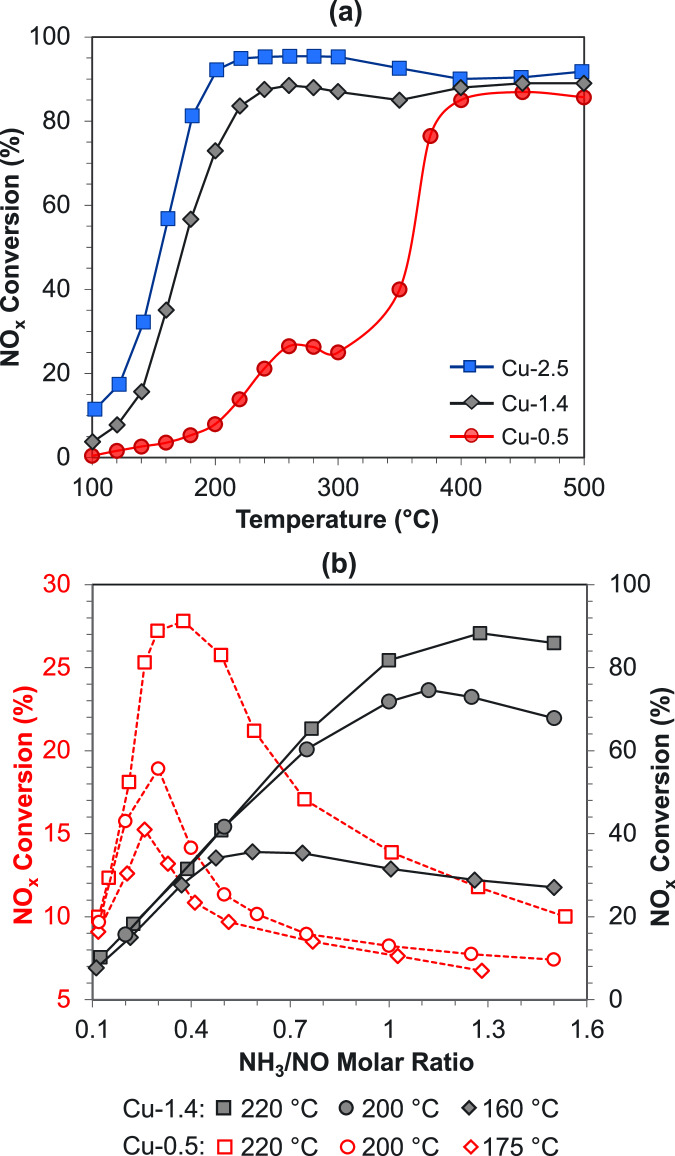
NH_3_ inhibition effects on NO_x_ conversion over Cu-CHA catalysts. **a** NO_x_ conversion during standard SCR as a function of temperature for three Cu-CHA catalysts with different copper loadings: Cu-0.5 (0.48 wt% Cu), Cu-1.4 (1.39 wt% Cu), and Cu-2.5 (2.48 wt% Cu), measured at NH_3_:NO = 1. **b** NO_x_ conversion as a function of NH_3_:NO molar ratio (*x*-axis) for Cu-1.4 and Cu-0.5 at multiple temperatures: 220 °C, 200 °C, and 160 °C for Cu-1.4; and 220 °C, 200 °C, and 175 °C for Cu-0.5. Feed conditions: 350 ppm NO, 10% O_2_, 3% H_2_O and varying NH_3_ levels to achieve desired NH_3_-to-NO_x_ ratios, at 150,000/h gas hourly space velocity (GHSV).

Figure 1a presents the standard SCR NOₓ conversion as a function of temperature. As expected, NOₓ conversion at a given temperature increases with Cu loading. Cu-0.5 exhibits the well-known “seagull” shape behavior with a mid-temperature activity dip, which reflects the temperature-dependent shift from quasi-homogeneous catalysis (Cu^I^ mobility enabled by NH_3_ solvation) below 250 °C to heterogeneous SCR above 300 °C involving less mobile, framework-bound Cu sites^4^. The low-temperature SCR OHC involves oxygen activation on a dimeric oxygen-bridged copper species, requiring isolated Cu^I^ ions to migrate between cages to reside in proximity with another Cu^I^ ion. Negri and coworkers used in-situ X-ray absorption and infrared spectroscopies, along with DFT calculations, to reveal that these dimers were formed by two mobile linear [Cu^I^(NH_3_)_2_]^+^ complexes, consisting of a side-on μ-η^2^,η^2^-peroxo diamino copper(II) structure ([Cu^II^_2_(NH_3_)_4_(O_2_)]^2+^)^12^. At low Cu loading (Cu-0.5), the reduced likelihood of Cu–Cu encounters limits the formation of dimeric Cu-oxo species needed for efficient OHC, intensifying this kinetic bottleneck. This explains the lower activity and pronounced seagull dip in these samples. In contrast, Cu-1.4 shows only a subtle dip, while Cu-2.5 displays a monotonic increase, reflecting reduced kinetic constraints with higher Cu content.

Figure 1b presents the SCR NOₓ conversion on Cu-1.4 and Cu-0.5 as a function of ANR across temperatures ranging from 160 to 220 °C. Both samples exhibit a clear inhibition regime at elevated ANR. Two systematic trends emerge: (a) for a given catalyst, the critical ANR—defined as the ratio beyond which activity begins to decline − decreases with temperature (e.g., for Cu-1.4: 1.25 at 220 °C, 1.1 at 200 °C, 0.6 at 160 °C); and (b) at a given temperature, the critical ANR decreases with Cu loading (e.g., 1.25 for Cu-1.4 vs. 0.4 for Cu-0.5 at 220 °C). These trends highlight a strong interplay between Cu mobility and NH_3_ concentration. We selected Cu-0.5 for further kinetic evaluations as it provides a copious amount of data points in the kinetically limited region (<20% NO_x_ conversion). It is essential to note that, while we require data under kinetic limitation for reliable rate parameter estimations (i.e., negligible reactant or product mass transfer limitation), the NH_3_ inhibition process is not only confined to low NO_x_ conversions; Cu-1.4 exhibits inhibition at conversions as high as 85%, underscoring the practical importance of this effect. Further supporting this notion, the SCR light-off and light-down performance of Cu-2.5, shown in Supplementary Fig. 2 and discussed in Supplementary Note 2, illustrates significant NH_3_ inhibition below 220 °C during transient operations typical of real-world driving conditions. This sample has a high Cu loading similar to fresh commercial samples, again demonstrating the practical importance of understanding NH_3_ inhibition in SCR systems.

**Fig. 2:**
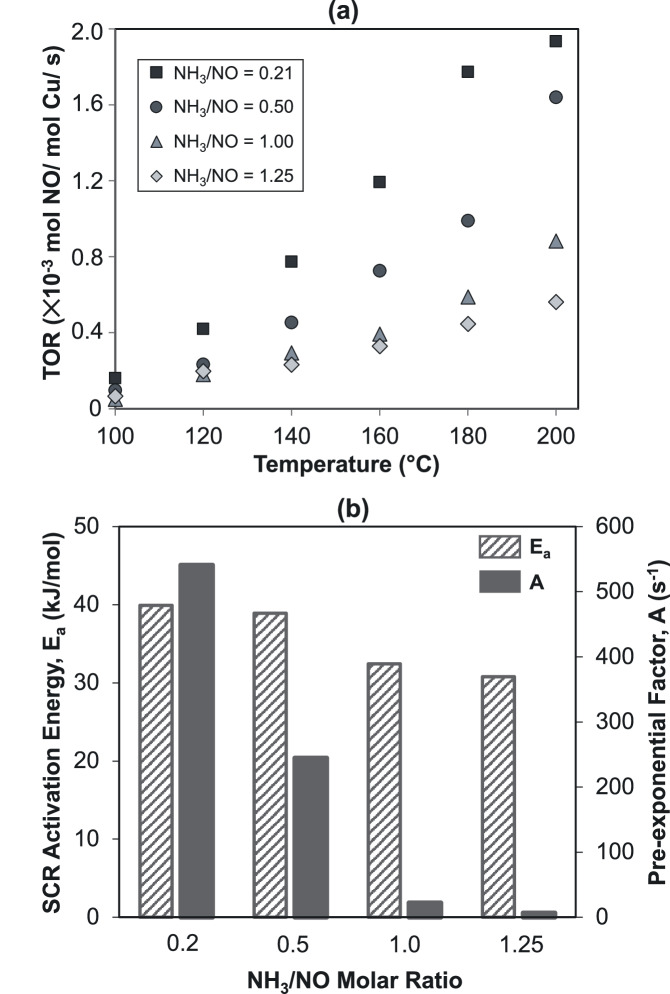
Kinetic parameters for standard SCR over Cu-CHA (Cu-0.5). **a** NO Turnover rates (TOR), and **b** apparent activation energy and pre-exponential factors for NO_x_ conversion derived from temperature-dependent SCR measurements on the Cu-0.5 catalyst (0.48 wt% Cu). Kinetic parameters were obtained by fitting NO_x_ conversion data under varying NH_3_:NO_x_ ratios (ANR). Feed conditions: 350 ppm NO, 10% O_2_, 3% H_2_O, and balance gas, with NH_3_ adjusted to achieve the desired ammonia-to-NO_x_ ratio (ANR), at a gas hourly space velocity (GHSV) of ~150,000 h⁻¹.

To quantify the effect of NH_3_ on intrinsic SCR rates, we evaluated low-temperature turnover rates (TOR) for Cu-0.5 as a function of ANR (Fig. 2a). TORs decline monotonically with increasing ANR. Corresponding NO_x_ conversion vs. temperature data in Supplementary Fig. 3a, b reveal two opposing effects of NH_3_ concentration: while higher ANR increases NOₓ conversion above 350 °C due to faster kinetics and complete NH₃ consumption, conversion drops markedly below 220 °C. Above 350 °C, the reasons behind this effect are clear: accelerated kinetics increase conversion rates for both NO_x_ and NH_3_, and at ANR < 1.0, NH_3_ is entirely consumed. In addition to NH_3_ being the limiting reactant at these higher temperatures, there is also a contribution from non-selective NH_3_ oxidation; the latter is evident from continued NO_x_ conversion increases when ANR increases from 1.0 to 1.25. Conversely, below 220 °C, a decreased NO_x_ conversion with increasing ANR clearly demonstrates a strong NH_3_ inhibition effect; this is in-line with slow SCR kinetics following a quasi-homogeneous mechanism requiring mobile Cu ions. The low-temperature activity data in Fig. 2a were further analyzed utilizing a first order kinetic equation, $r=\,\frac{F}{W}\,(-{{{\mathrm{ln}}}}\left(1-X\right))$, where F is NO_x_ flow rate (mol/s), *W* is catalyst amount (g) and *X* is fractional NO_x_ conversion. The Arrhenius equation, $k=\,\frac{r}{{[{NO}]}_{0}}={A}{e}^{-\,\frac{{E}_{a}}{{RT}}}$, was used to calculate apparent activation energy (*E*_*a*_) and pre-exponential factor (*A*), where ${[{NO}]}_{0}$ is the concentration of NO in the feed. Supplementary Fig. 3c presents the Arrhenius plot utilized to estimate SCR apparent activation energies and pre-exponential factors presented in Fig. 2b. The apparent activation energies exhibit a slight, gradual decrease with increasing ANR. These variations, however, are not large and suggest a consistent reaction mechanism and rate-determining step across all cases. Gao et al. demonstrated that the apparent SCR activation energy is typically around 80 kJ/mol for high Cu-loading Cu-CHA, decreasing to approximately 35 kJ/mol in low Cu-loading scenarios where the oxidation half-cycle (OHC) becomes rate-limiting^20^. In our case, the apparent activation energies at all ANRs range between 30 and 40 kJ/mol, suggesting that SCR on Cu-0.5 is already in the OHC-limited regime. The slight decrease in activation energy with rising ANR may indicate further increases in OHC limitations, a notion we will discuss in more detail shortly. In contrast, the pre-exponential factors show a rapid decline with increasing ANR, dropping by two orders of magnitude as this ratio increases from 0.2 to 1.25. This decline indicates a significantly greater entropic barrier to reaction with increased ANR and suggests significant impedance of collisions between active Cu sites and reactant molecules at higher NH_3_ concentrations. Therefore, the observed NH_3_ inhibition of the SCR reaction is not due to a significant change in the reaction mechanism, but rather a decrease in the efficient utilization of active Cu sites.

The interplay between the reduction half-cycle (RHC) and the oxidation half-cycle (OHC) determines the overall SCR kinetics. Different feed NH_3_ concentrations can impact either one or both redox half-cycles. To further resolve the influence of NH_3_ on the individual redox half-cycles, we adopted a method recently introduced by Krishna and coworkers^14,19,32^. This approach models steady-state SCR rates as a function of O_2_ partial pressure using a Langmuir-type rate law to decouple the apparent rate constants for Cu oxidation and reduction.

1 $$-\frac{{r}_{{NO}}}{\left[{{Cu}}_{{tot}}\right]}=\frac{{k}_{{Ox}}\,{k}_{{red}}\,{p}_{{O}_{2}}}{{k}_{{red}}\,+\,{k}_{{ox}}\,{p}_{{O}_{2}}}$$

**Fig. 3:**
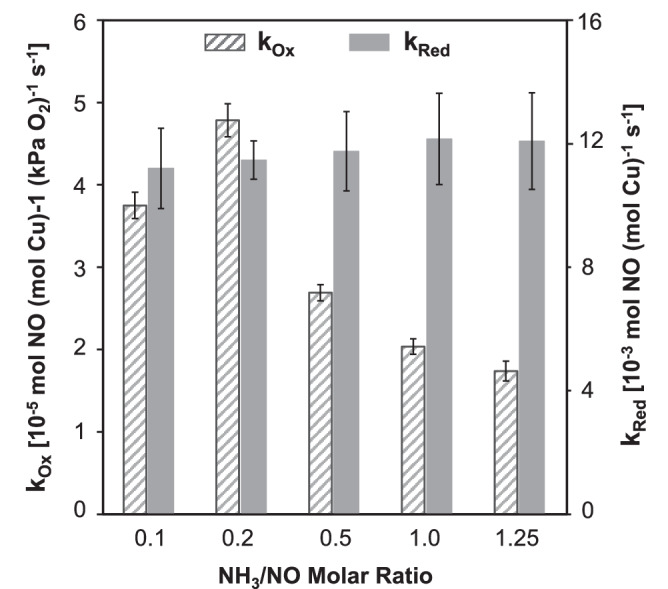
Oxidation and reduction rate constants for Cu-CHA (Cu-0.5). Rate constants for Cu oxidation (k_ox_) and reduction (k_Red_) determined for the Cu-0.5 catalyst (0.48 wt% Cu) using Langmuir-type regression of standard SCR reaction rates as a function of O_2_ partial pressure (data shown in Supplementary Fig. 4). Error bars represent *R*^2^ error from the regression.

Here, ${k}_{{ox}}$ and ${k}_{{red}}$ represent apparent rate constants for Cu oxidation (Cu^I^ → Cu^II^) and reduction (Cu^II^ → Cu^I^), respectively, and ${p}_{{O}_{2}}$ in the oxygen partial pressure. At the extremes of this analysis, high ${p}_{{O}_{2}}$ approaches $-\frac{{r}_{{NO}}}{\left[{{Cu}}_{{tot}}\right]}=\,{k}_{{red}}$ and low ${p}_{{O}_{2}}$ approaches $-\frac{{r}_{{NO}}}{\left[{{Cu}}_{{tot}}\right]}=\,{k}_{{ox}}\,{p}_{{O}_{2}}$. Supplementary Fig. 4 presents the steady state SCR TOR at 200 °C as a function of oxygen partial pressure at various ANRs, alongside their model fits. The extracted rate constants are depicted in Fig. 3. Interestingly, the Cu reduction rate constant (${k}_{{Red}}$) is largely insensitive to ANR. In contrast, the Cu oxidation rate constant (${k}_{{Ox}}$) is ANR dependent: ${k}_{{Ox}}$ initially increases as the ANR rises from 0.1 to 0.2, then consistently decreases with further increases in ANR. The critical ANR at 200 °C, observed to be between 0.2 and 0.4 (as seen in Fig. 1b), suggests that the initial rise in ${k}_{{Ox}}$ with ANR enhances SCR activity. A modest NH₃ concentration enhances OHC kinetics up to a critical ANR (~0.4), beyond which excessive NH₃ suppresses Cu oxidation. These trends reinforce the view that NH_3_ inhibition primarily stems from impairment of the OHC, while the reduction process remains relatively unaffected. Ideally, this analysis would be conducted at different temperatures to estimate the activation energy of each half-cycle. However, this would require accurate resolution of NO concentrations as low as 1–2 ppm, which is outside of the capability of our MKS FTIR gas analyzer. Therefore, we adopted an alternate approach described below involving transient response experiments with associated kinetic models.

In addition, the Langmuirian-type expression applied to steady-state analysis is not meant to represent the elementary Cu^I^ kinetics. Its purpose is to describe how the observed macroscopic SCR rate depends on O_2_ pressure under steady-state conditions. Krishna et al. have shown that for samples with low Cu-loading, like the ones used in our study, the apparent O_2_ reaction order is close to unity^14^. On the contrary, the elementary Cu(I) kinetics has a second-order dependence on Cu, as shown by O_2_-pulse experiments described in Supplementary Note 3 and Fig. S5.

To quantify the temperature dependence of these rate constants, we conducted transient kinetic experiments inspired by prior work from Deka et al.^33^ and Nasello et al.^34^. Supplementary Note 4 (and Supplementary Fig. 6) provides a detailed description of the methodology. Briefly, the RHC was probed by initiating NO flow over a NH_3_-saturated oxidized Cu-0.5 sample. Since the RHC reaction can be expressed as NO + NH_3_ + ZCu^II^OH → N_2_ + 2H_2_O + ZCu^I^, each mole of NO consumed corresponds to one mole of Cu^II^ reduced^33,35^. The NO transient obtained from these experiments therefore provides kinetic information on Cu^II^ reduction. We used a kinetic model assuming the plug flow reactor as 20 CSTRs in series and used it to fit NO transients and estimate RHC rate constants (k_RHC_). After completing each RHC NO transient, O_2_ was introduced to perform SCR. The steady state NO_x_ concentration achieved during such an SCR, along with the amount of Cu^I^ re-oxidized during RHC → SCR transition, was used to estimate OHC rate constant (k_OHC_) (further details in Supplementary Note 4).

**Fig. 4:**
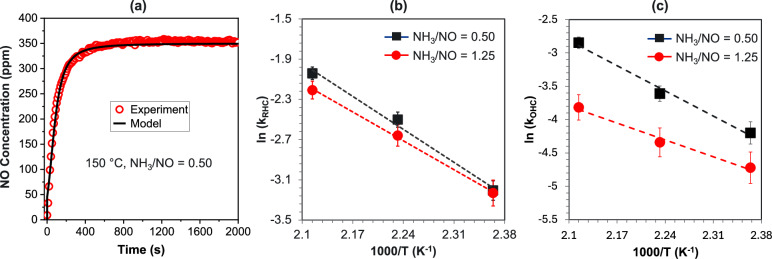
Transient analysis of reduction and oxidation kinetics over Cu-CHA (Cu-0.5). **a** Experimental NO transient response (circles) obtained during NO + NH_3_ titration, along with the corresponding model fit (solid line). **b** Reduction half-cycle (RHC) rate constants derived from model fits to NO transient data at ammonia-to-NO_x_ ratios (ANR) of 0.5 and 1.25 (transients shown in Supplementary Fig. 7). **c** Oxidation half-cycle (OHC) rate constants obtained from steady-state SCR measurements and post-SCR RHC transient analysis (data shown in Supplementary Fig. 8) for the Cu-0.5 catalyst (0.48 wt% Cu). Error bars represent *R*^2^ error from the regression.

Figure 4a presents an example RHC NO transient and its model fit at ANR = 0.50 and 150 °C, and Supplementary Fig. 7 shows all these transients across varying ANRs and temperatures. Additionally, Supplementary Fig. 8 shows the subsequent SCR NO transients. The calculated k_RHC_ and k_OHC_, presented as Arrhenius plots in Fig. 4b, c, respectively, reveal a minor decrease in k_RHC_ with increasing ANR, but a significantly pronounced decrease in k_OHC_, consistent with the trends in Fig. 3. The apparent activation energies and pre-exponential factors for RHC and OHC, derived from these rate constants, are summarized in Table 1. These activation energies align with those reported by Jones et al. for RHC and OHC under stoichiometric NH_3_/NO conditions on Cu-CHA with a Cu density of less than 0.1 per cage, akin to Cu-0.5 in this study^14^. The RHC activation energy remains relatively unchanged with increasing ANR, and the pre-exponential factor decreases modestly by a factor of ~4, which is insufficient to explain the ~100× decrease observed during steady-state SCR. Conversely, the OHC pre-exponential factors exhibit ~100× decrease when ANR is increased, clearly indicating that NH_3_ primarily inhibits SCR by slowing down OHC kinetics. The activation energies for OHC, estimated from these transient experiments, also show a decrease with increasing NH_3_/NO, a trend similarly observed for SCR activation energies.

**Table 1:** Apparent activation energies (*E*_*a*_) and pre-exponential factors (*A*) for the reduction half-cycle (RHC) and oxidation half-cycle (OHC) determined for the Cu-0.5 catalyst (0.48 wt% Cu)

Rate parameter	Reduction half cycle		Oxidation half cycle	
	NH_3_/NO 0.50	NH_3_/NO 1.25	NH_3_/NO 0.50	NH_3_/NO 1.25
*E*_*a*_ (kJ/mol)	39	35	45	31
*A*	2.7 × 10^3^	6.5 × 10^2^	4.9 × 10^3^	4.3 × 10^1^

Our findings so far suggest that NH_3_ inhibition of SCR is primarily caused by impaired OHC kinetics. The following sections elaborate on the molecular-level origins of this effect. As previously noted, the OHC proceeds via two steps: (a) inter-cage diffusion of linear [Cu^I^(NH_3_)_2_]^+^ complexes to pair up with another [Cu^I^(NH_3_)_2_]^+^ complex in the same cage, (b) two [Cu^I^(NH_3_)_2_]^+^ complexes in close proximity activate an O_2_ molecule to form a [Cu^II^_2_(NH_3_)_4_(O_2_)]^2+^ dimeric species. Both Cu^I^ and N have atomic radii >0.5 Å, and since Cu-N and N-H bond lengths are approximately 2 Å and 1 Å, respectively, the linear [Cu^I^(NH_3_)_2_]^+^ complexes need to be at an appropriate orientation to be able to pass through the ~3.8 Å openings of *cha* cages^4^. Given that such orientation is random, transport of these ions, particularly at low Cu loading, can be slow. In such cases, the intercage transfer of [Cu^I^(NH_3_)_2_]^+^ becomes entropically costly, and hence the dimeric Cu moiety formation rather than O_2_ activation determines the SCR kinetics^5,22,36^. Consequently, slow OHC kinetics at high ANR suggest a higher barrier to [Cu^I^(NH_3_)_2_]^+^ diffusion. Evidence from Supplementary Fig. 9, which shows NO_x_ and NH_3_ conversion data on Cu-0.5 at 220 °C across varying ANRs, reveals that the onset of inhibition coincides with excess gas-phase NH_3_ (i.e., NH_3_ conversion <100%). This unreacted NH_3_ likely saturates the CHA cages and 8-membered rings, physically hindering Cu^I^ migration. Additionally, NH_3_-TPD results (Supplementary Fig. 10 and Supplementary Note. 5) confirm that NH_3_ adsorption on Cu sites increases substantially at high ANRs. These findings motivated our use of DFT calculations to further elucidate the effect of excess NH_3_ on Cu ion mobility.

### Density functional theory

**Fig. 5:**
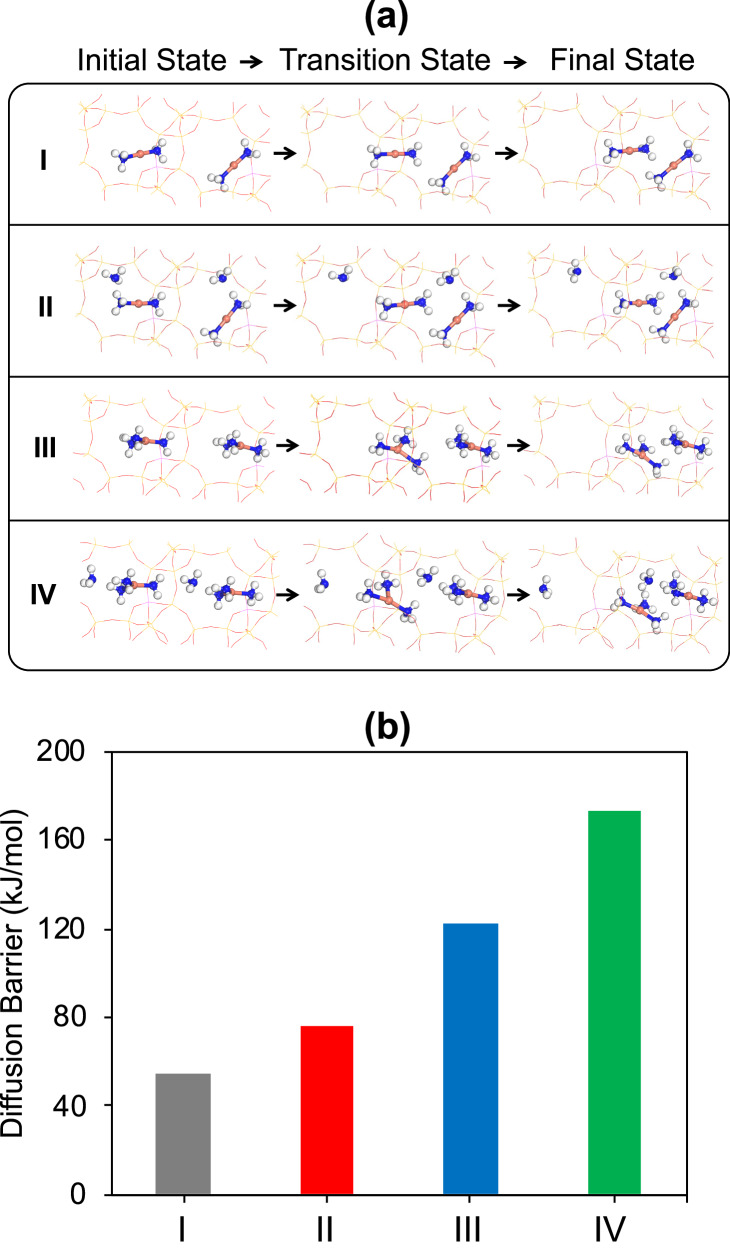
DFT insights into NH_3_-assisted Cu^I^(NH_3_)_x_ (x = 2 or 3) diffusion in Cu-CHA. **a** Optimized geometries of the initial state, transition state, and final state for Cu^I^(NH_3_)_x_ diffusion in Cu-CHA at 473 K with varying NH₃ coordination environments: I, two ligated NH_3_; II, two ligated NH_3_ with one additional free NH_3_; III, three ligated NH_3_; IV, three ligated NH_3_ with one additional free NH_3_. **b** Corresponding diffusion barriers for Cu^I^ ions as a function of the number of ligated and free NH₃ molecules. Atomic color scheme: Cu (orange), N (blue), H (white), O (red), Si (yellow), Al (pink).

To probe the molecular origins of NH_3_ inhibition, we performed DFT calculations focused on how excess NH_3_ affects the mobility of Cu(I) species in Cu-CHA. NH_3_ can interact with Cu ions in two primary configurations: (1) coordination (ligated NH_3_), which makes a chemical bond with the Cu ions to form [Cu^I^(NH_3_)_x_]^+^, and (2) weak physisorption within the zeolite cage as free NH_3_. To evaluate the impact on diffusion, we computed energy barriers for [Cu^I^(NH_3_)_x_]^+^ migration across an 8-membered ring (8-MR) window separating adjacent CHA cages, both with and without the presence of excess NH_3_. Figure 5a presents the initial, transition, and final geometries for the following cases:

(I) [Cu^I^(NH_3_)_2_]^+^,
(II) [Cu^I^(NH_3_)_2_]^+^ with 1 free NH_3_,
(III) [Cu^I^(NH_3_)_3_]^+^,
(IV) [Cu^I^(NH_3_)_3_]^+^ with 1 free NH_3_.

Figure 5b and Supplementary Figs. 11 and 12 present the diffusion barriers in each of these cases. For the base case of [Cu^I^(NH_3_)_2_]^+^, the calculated diffusion barrier is ~45−55 kJ/mol, close to the measured SCR activation energy range shown in Fig. 2, suggesting that [Cu^I^(NH_3_)_2_]^+^ diffusion is the rate-limiting step in this process. When an additional NH_3_ molecule enters the CHA cage, it may either remain free or weakly bind to the Cu ion. This was validated through NH_3_-temperature programmed desorption (NH_3_-TPD) on a reduced Cu-0.5 sample and the corresponding H-CHA support. In these experiments, NO and NH_3_ were flowed over the catalyst at 180 °C to reduce all Cu^II^ sites to Cu^I^ while simultaneously adsorbing NH_3_. The subsequent NH_3_-desorption phase included a 3-h isothermal step followed by a ramp up to 550 °C under an inert N_2_ flow. As detailed in Supplementary Note 6 and Supplementary Fig. 13, we estimated the NH_3_:Cu molar ratio for chemisorbed NH_3_ on Cu^I^ sites to be approximately 2:1 while that for weakly-bound (physisorbed) NH_3_ was approximately 0.75:1, giving a total NH_3_:Cu ratio of ~2.75:1. This indicates that under excess NH_3_ conditions, roughly 25% of the reduced Cu sites are present as [Cu^I^(NH_3_)_2_]^+^ and the remaining 75% as [Cu^I^(NH_3_)_3_]^+^. It is worth noting that the third NH_3_ ligand on [Cu^I^(NH_3_)_3_]^+^ is weakly bound and only present under conditions with excess NH_3_, which occurs when the ANR exceeds the critical value. In both scenarios, the DFT-computed diffusion barrier increases relative to the base [Cu^I^(NH_3_)_2_]^+^ case, suggesting that excess NH_3_ hinders Cu ion mobility. Notably, the diffusion barrier for [Cu^I^(NH_3_)_3_]^+^ is ~45 kJ/mol higher than that of [Cu^I^(NH_3_)_2_]^+^ with 1 free NH_3_, likely due to the increased bulk of the Cu complex, which reduces its diffusivity. The highest diffusion barrier, approximately 173.7 kJ/mol, occurs when 3 ligated NH_3_ are present along with a fourth free NH_3_, rendering [Cu^I^(NH_3_)_3_]^+^ diffusion energetically unfavorable. These findings clearly indicate that excess NH_3_ significantly impedes Cu^I^ ion diffusion across the CHA cages, thereby limiting the formation of reactive Cu dimers essential for OHC and suppressing overall SCR activity.

**Fig. 6:**
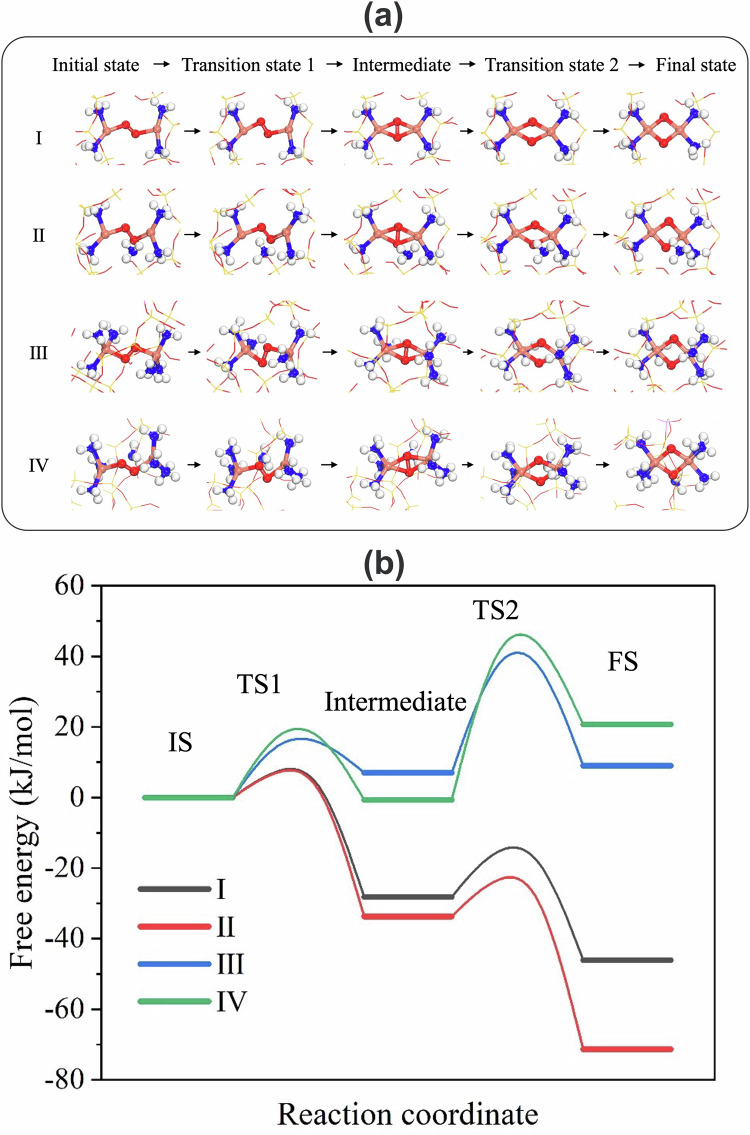
Effect of NH_3_ coordination on O_2_ activation energetics over Cu-CHA. **a** DFT-optimized geometries along the O_2_ activation pathway on Cu^I^(NH_3_)_x_ (*x* = 2 or 3) sites at 473 K, including the initial state (IS), O_2_ binding transition state (TS1), intermediate, O–O activation transition state (TS2), and final state (FS), for different NH_3_ coordination environments: I, two ligated NH_3_; II, two ligated NH_3_ with one additional free NH_3_; III, three ligated NH_3_; IV, three ligated NH_3_ with one additional free NH_3_. **b** Corresponding free energy barriers for O_2_ binding (TS1) and O–O activation (TS2) as a function of the number of ligated and free NH_3_ molecules. Atomic color scheme: Cu (orange), N (blue), H (white), O (red), Si (yellow), Al (pink).

The O_2_-driven oxidation half-cycle requires two [Cu^I^(NH_3_)_2_]^+^ species to come together and form an O_2_-bridged dimer^5,37^. [Cu^I^(NH_3_)_2_]^+^ oxidation probed via O_2_-pulse experiments presented in Supplementary Fig. 14 shows that excess NH_3_ leads to a decreased O_2_ uptake by the Cu^I^ sites. Because both the dimer formation and the subsequent O_2_ activation may be rate-limiting, we next investigate these two elementary steps after the two Cu ions diffuse into the same CHA cage (Fig. 6). For [Cu^I^(NH_3_)_2_]^+^(Case I), the reaction barrier for O_2_ binding and O-O activation are 8.7 and 17.1 kJ/mol, respectively. When an additional free NH_3_ is present (Case II), the corresponding barriers are 8.4 and 15.2 kJ/mol, indicating that excess free NH_3_ has a negligible effect on O_2_ binding and O-O activation on [Cu^I^(NH_3_)_2_]^+^. In contrast, for [Cu^I^(NH_3_)_3_]^+^ (Case III), the barriers for O_2_ binding and O-O activation increase to 17.2 and 35.7 kJ/mol, respectively, demonstrating that excess ligated NH_3_ inhibits both processes. When an additional free NH_3_ molecule is introduced (Case IV), the barriers further increase to 22.3 and 48.2 kJ/mol, indicating that excess free NH_3_ suppresses O_2_ binding and O-O activation on [Cu^I^(NH_3_)_3_]^+^. However, as shown in the preceding analysis (Fig. 5b), the diffusion barriers for [Cu^I^(NH_3_)_2_]^+^ and [Cu^I^(NH_3_)_3_]^+^ across the eight-membered ring are 55 and 123 kJ/mol, respectively, which are significantly higher than the barriers associated with O_2_ binding and O-O activation, suggesting that Cu^I^ ion diffusion is the rate-controlling step of the overall reaction.

In summary, excess NH_3_ in [Cu^I^(NH_3_)_3_]^+^ modestly increases the barriers for O_2_ binding and O-O activation, but these barriers remain substantially lower than the corresponding diffusion barriers across the 8-membered ring. This indicates that Cu^I^ ion diffusion, which is strongly hindered by excess NH_3_ through increased ligation and steric bulk, is the dominant rate-controlling step governing Cu dimer formation and, consequently, the overall SCR activity.

### Operando EPR spectroscopy

**Fig. 7:**
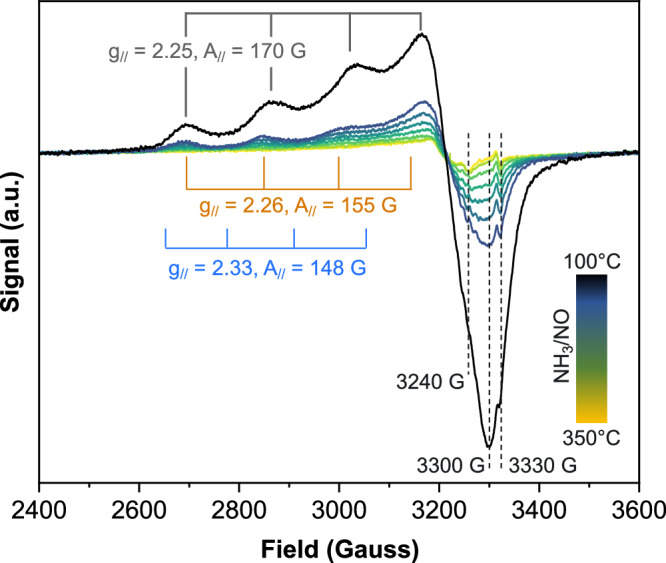
Temperature-dependent operando EPR of Cu-CHA under standard SCR conditions. Operando EPR spectra collected on the Cu-0.5 catalyst (0.48 wt% Cu) under steady-state standard SCR at an ammonia-to-NOₓ ratio (ANR) of 1 over a temperature range of 100–350 °C (100, 160, 180, 200, 220, 250, 300, and 350 °C). Spectral features correspond to Cu²⁺ species present under reaction conditions. Feed conditions: 500 ppm NO, 500 ppm NH₃, 10% O₂, 3% H₂O, balance gas, at a gas hourly space velocity (GHSV) of 400,000 h⁻¹.

The state of Cu^II^ ions in Cu-0.5, including coordination environment and oxidation state, was investigated via *operando* electron paramagnetic resonance (EPR) under SCR conditions, across a range of temperatures and ANRs. Figure 7 shows EPR spectra collected at fixed ANR = 1.0 and temperature range of 100–350 °C. In the high field region, three overlapping signals are observed at 3240 G, 3300 G and 3330 G. The 3330 G signal is an instrumental artifact, and hence is not associated to any catalytically relevant process (see details in Supplementary Note 7 and Fig. 15). The 3300 G EPR signal is assigned to Cu^II^-ions with high Cu–N coordination, such as Cu^II^(NH_3_)_4_ or Cu^II^OH(NH_3_)_3_, while the 3240 G signal arises from mobility-restricted Cu^II^-ions with mixed Cu–N/Cu–O_L_ coordination. Cu ions are readily solvated by NH_3_ at low temperatures as evidenced by the high Cu–N coordination signature at 3300 G. As the temperature increases, a gradual decrease in Cu–N coordination and an increase in Cu–O_L_ coordination (O_L_ = lattice oxygen atom) is apparent. This trend reflects the progressive immobilization of Cu^II^ ions described by the following reaction: Cu^II^(NH_3_)_n_ ⇔ Cu^II^(O_L_)(NH_3_)_n-1_ + NH_3_^38–40^. The contribution from C-N coordination at 3300 G decreases with increasing temperature, persisting through ~220 °C, and the contribution from Cu–O_L_ coordination at 3240 G increases above ~220 °C. This is consistent with the activity trends in Fig. 1. The high field region in the EPR spectra also displays superhyperfine features between 160 and 300 °C, which we will discuss shortly.

Mobility-restricted Cu^II^-ions have anisotropic characteristics that give rise to hyperfine splitting features in *x* and *y* directions with varied tensor values based on changes in their coordination environment. These features offer further insights into Cu coordination environments. At 100 °C, the observed features with g_//_ = 2.25 and A_//_ = 170 G are attributable to Cu^II^(NH_3_)_n_ (n ≤ 5) complexes^40^. With increasing temperature, the hyperfine features gradually shift to lower fields. Consequently, spectra above 160 °C consist of two overlapping hyperfine signals, with tensor values of g_//_ = 2.26, A_//_ = 155 G and g_//_ = 2.33, A_//_ = 148 G, with the latter becoming more prevalent at higher temperatures. The feature at g_//_ = 2.26, A_//_ = 155 G comes from largely anisotropic Cu^II^-ions that contain mixed Cu–N/Cu–O_L_ coordination^20^. The tensor values g_//_ = 2.33, A// = 148 G are close to those reported for para-Z_2_Cu (coordinated to Al-pair with Al-Si-Si-Al linkage) in previous studies, indicating that the associated Cu^II^-ions primarily contain O_L_ ligands^39,41–43^. These features mainly arise at 350 °C, i.e., above the NH_3_ solvation region.

**Fig. 8:**
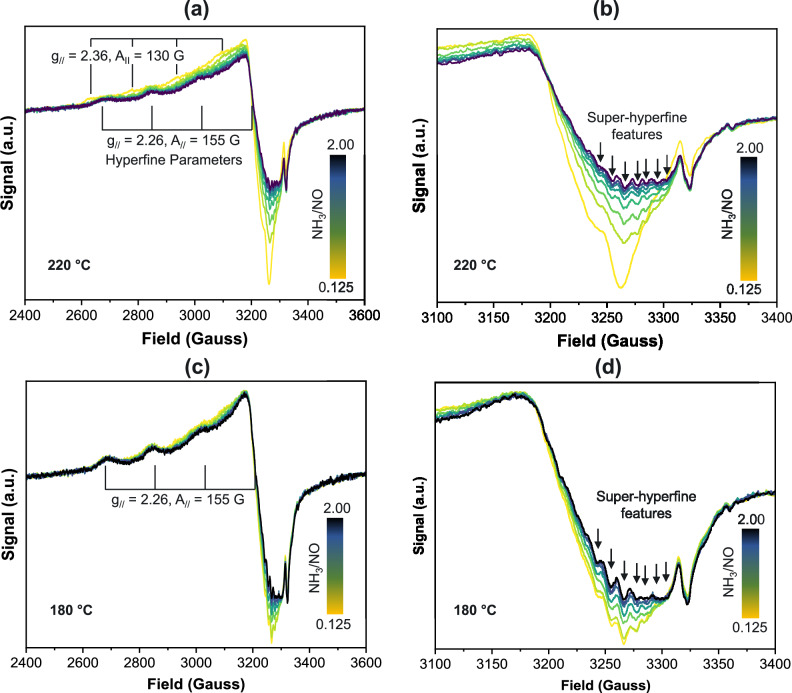
Effect of NH_3_:NO_x_ ratio on Cu speciation from operando EPR. **a**, **b** Operando EPR spectra collected on the Cu-0.5 catalyst (0.48 wt% Cu) under steady-state standard SCR at 220 °C with varying ammonia-to-NO_x_ ratios (ANR). **c**, **d** Corresponding spectra acquired at 180 °C under identical conditions. Changes in spectral features reflect variations in Cu^2+^ speciation with NH_3_:NO_x_ ratio and temperature. 500 ppm NO, varying NH_3_ to achieve the desired ANR, 10% O_2_, 3% H_2_O, balance gas, at a space velocity (SV) of 400,000 h^-1^.

To examine NH₃ effects more directly, we collected EPR spectra under varying ANRs (0.125–2.0) at two temperatures: 220 °C (Fig. 8a, b) and 180 °C (Fig. 8c, d). At 220 °C (Fig. 8a), the low field region displays two overlapping hyperfine features with spin-Hamiltonian parameters g_//_ ≈ 2.26, A_//_ ≈ 155 G and g_//_ ≈ 2.36, A_//_ ≈ 135 G. These two features were used to fit each EPR spectrum, and the detailed method and results are described in Supplementary Note 8 and Supplementary Figs. 16–18. The fitting results show that the first feature dominates at higher ANR, while the second is more prominent at the lowest ANR. We have already assigned the first hyperfine feature to Cu^II^-ions with mixed Cu–N/Cu–O_L_ coordination. The second feature has similar tensor values to those seen in ex-situ spectra of Cu-0.5 shown in Supplementary Note 9 and Fig. 19, which suggests that Cu^II^-ions at low ANRs (as low as 0.125) have negligible NH_3_ ligands—at least within the EPR’s detection limit. This explains the lack of NH_3_ inhibition at this condition since (i) NH_3_ conversion is close to 100% and (ii) NH_3_ likely reacts with NO on Cu ions with a short residence time that EPR cannot detect, resulting in a low inventory of unreacted NH_3_ on the catalyst.

The high-field region of the 220 °C spectra (Fig. 8b) reveals “spikes” indicating superhyperfine features at ANR ≥ 0.5. These features arise from the coupling between the unpaired electron in Cu^II^ and the nuclear spin of ^14^N in NH_3_ ligands, leading to (2n*I* + 1) superhyperfine lines (where *n* is the number of NH_3_ ligands, *I* is the nuclear spin, *I* = 1 for ^14^N)^20,40,44^. For ANR ≥ 0.5, we observe at least seven such signals, suggesting that Cu^II^ ions exist as Cu(OH)(NH_3_)_3_ (i.e., *n* = 3 NH_3_ ligands). Although seven superhyperfine lines are visible, it is possible that there are more, with their intensity being too low, especially at the extremes of the high-field signal. For example, Wu et al. observed 9 such features on low Cu-CHA samples with low Cu-loading (similar to Cu-0.5 in this work)^41^. Thus, it is plausible that some Cu ions could exist as Cu(NH_3_)_4_ (i.e., *n* = 4 with 9 superhyperfine signals) at the highest ANRs. However, our fundamental conclusions outlined below remain unchanged irrespective of whether Cu^II^ions are present as Cu(NH_3_)_4_ or Cu(OH)(NH_3_)_3_. Moreover, prior studies have established that Cu(NH_3_)_4_ complexes are not redox active on their own and need to hydrolyze to form Cu(OH)(NH_3_)_3_ complexes before they can participate in subsequent RHC^3,4,7^. The high-field spectra at ANR = 0.125 do not display any superhyperfine signals, and only three to five discernible features are seen at ANR = 0.25 (indicating 1 or 2 NH_3_ ligands on Cu). These observations clearly demonstrate that the number of NH_3_ ligands on Cu^II^-sites is negligible at ANR = 0.125 but increases with higher NH_3_ levels in the feed. An NH_3_ coordination = 3 of three on Cu^II^-sites is achieved at ANR = 0.5 and remains constant with further increases in ANR. These trends of Cu-N/Cu-O coordination as a function of ANR as inferred from superhyperfine features agree well with those obtained from hyperfine parameters and fit results shown in Fig. 8a and Supplementary Figs. 16–18.

Interestingly, at 220 °C, where all these spectra in Fig. 8a, b were collected, the SCR activity results shown in Fig. 1 indicate that the critical ANR is 0.5—which is close to where EPR spectra reveal maximized NH_3_ coordination on Cu^II^ ions. When the ANR is <0.5, Cu^II^ ions exhibit fewer NH_3_ ligands, reducing the likelihood of forming mobile Cu^I^(NH_3_)_2_ complexes. Consequently, fewer Cu^I^ ions occupy the same cage, thereby limiting OHC kinetics. This observation aligns well with the data in Figs. 1 and 3, showing that both SCR activity and OHC rate constant increase with rising ANR up to the critical value of 0.5. Beyond this critical ANR, although the NH_3_ coordination of Cu^II^ ions remains maximized, the excess NH_3_ begins to impede the mobility of Cu^I^ ions, as demonstrated by our DFT calculations. As a result, OHC kinetics slow, SCR NO_x_ conversion drops, and the observed NH_3_ inhibition of SCR takes effect.

At 180 °C, the low field region (Fig. 8c) has only one discernible hyperfine feature that appears at all ANRs. This feature is characterized by spin-Hamiltonian values of g_//_ = 2.26 and A_//_ = 155 G, attributed to Cu^II^-ions with mixed Cu–N/Cu–O_L_ coordination. The absence of the feature at g_//_ = 2.36 and A_//_ = 135 G, associated with Cu^II^-ions void of NH_3_ ligands (e.g., as observed at 220 °C), indicates higher Cu–N coordination at 180 °C. This higher Cu–N coordination is further supported by the high-field region shown in Fig. 8d, where at least 7 superhyperfine signals are visible at ANR as low as 0.25. The SCR activity data in Fig. 1 show that critical ANR decreases with decreasing temperature, which aligns with the EPR data, indicating that maximized NH_3_ coordination of Cu is achieved at a lower ANR at lower temperatures.

The double integration of the EPR signal is directly proportional to the number of EPR-visible Cu ions, making it a valuable tool for quantifying these sites^20^. Since SCR is not active at 100 °C on Cu-0.5 (e.g., as shown in Supplementary Fig. 3), we can assume that all Cu sites are in the Cu^II^ state at this temperature. Once SCR becomes active, a fraction of Cu^II^ sites reduces to Cu^I^. With this assumption, we can calculate the fractional composition of Cu^II^ as Cu^II^/Cu_Total_ from EPR as a function of reaction temperature and ANR, where Cu_Total_ is the total EPR signal at 100 °C. Supplementary Note 10 and Fig. 20 present Cu^II^/Cu_Total_ values at 180 °C and 220 °C across ANRs ranging from 0.125 to 2.0. At both temperatures, the Cu^II^/Cu_Total_ decreases as the ANR increases. At a given reaction condition, the fractions of Cu^I^ and Cu^II^ are determined by the relative kinetics of RHC & OHC and, generally speaking, a higher relative OHC kinetics (to RHC) will lead to greater fractional population of Cu^II^ ions^5,20^. Hence, our observation of decreasing Cu^II^/Cu_Total_ with increasing ANR suggests that OHC kinetics are relatively slower at elevated NH_3_ levels. Supplementary Fig. 20 also presents Cu^II^ distribution calculated for a high-Cu sample (Cu-2.5) under SCR conditions at 180 °C. The relevant *operando* EPR spectra and discussion are provided in Supplementary Note 11 and Supplementary Fig. 21. Interestingly, unlike Cu-0.5, the Cu^II^ fraction on Cu-2.5 does not decrease with increasing ANR. Wu et al. recently elucidated the degree of rate-control from NH_3_-solvated Cu ion transfer and redox kinetics on Cu-CHA catalysts with varying Cu content, and they demonstrated that Cu-transfer is not the rate-limiting factor for high Cu containing samples^20,41^. The low kinetic relevance of [Cu^I^(NH_3_)_2_]^+^ diffusion on SCR makes Cu-2.5 less susceptible to NH_3_-inhibition. All these findings agree well with our SCR kinetics, individual RHC and OHC rate parameters, and DFT calculations.

At the beginning of Section “SCR kinetics and NH_3_-inhibition”, we discussed how the field-aged (FA) catalyst exhibits a higher extent of NH_3_ inhibition than the degreened (DG) sample, with inhibition appearing at a substantially lower ANR. Field aging modifies Cu-CHA catalysts via two primary pathways: partial loss of active Cu through CuOₓ cluster formation and depletion of framework Al via dealumination, both of which restrict Cu ion mobility. The effect of reduced active Cu-sites has been already discussed with the help of Fig. 1. We independently probed the role of framework Al density by synthesizing a 0.5 wt% Cu-CHA catalyst with a higher Si/Al ratio (Si/Al = 36), compared to the Si/Al ≈ 12 samples used throughout this study. Increasing the Si/Al ratio lowers the density of charge-compensating Al sites and increases their average separation, thereby reducing Cu mobility. As shown in Supplementary Fig. 22, the Si/Al = 36 catalyst displays significantly stronger NH_3_ inhibition, with the critical ANR shifting to markedly lower values relative to the Si/Al ≈ 12 Cu-0.5 sample. Together with the FA-DG comparison, these results demonstrate that NH_3_ inhibition becomes more severe as Cu mobility decreases, whether mobility is restricted by lower Cu loading, reduced Al density, or combined aging effects. This observation reinforces the central conclusion that NH_3_ inhibition is governed by mobility-limited oxidation half-cycle kinetics.

The insights gained from this study inform several strategies to mitigate NH₃ inhibition of SCR on Cu-CHA catalysts. A key approach is maintaining a high Cu loading to avoid OHC limitations. Although fresh commercial catalysts often start with high Cu content (2–3 wt%), real-world aging decreases active Cu content, increasing NH_3_ inhibition risk over time. Thus, rational material design should also address other factors that alleviate [Cu^I^(NH_3_)]^+^ diffusion limitations, such as optimizing the Si/Al ratio, distribution of Al site density and Al-Al distances. For instance, recent work demonstrated that the stability of Cu pairs varies significantly with Al distribution, differing by up to 0.55 eV, with the most favorable Cu-pair formation occurring when the Al–Al distance is approximately 7.5 Å^36^. Along with material design considerations, control over operating conditions is also important. A high temperature, when feasible, helps reduce inhibition. Moreover, controlling the NH_3_:NO feed ratio is crucial; ratios above the critical point impair catalyst performance and lead to unwanted NH_3_ emissions and increased costs. These findings emphasize the importance of advanced urea dosing strategies, such as segmented NH_3_ dosing, which spatially distributes NH_3_ injection across multiple zones in the SCR system to balance reactivity and minimize localized inhibition. When combined with closed-loop feedback control, such dosing strategies can dynamically adjust NH_3_ feed under transient driving conditions. This is especially important for lean-burn and hybrid powertrains that operate at lower exhaust temperatures, where NH_3_ inhibition risks are amplified. Altogether, these strategies can help extend catalyst durability, ensure compliance with emissions regulations, and support next-generation low-carbon mobility.

In summary, this study reveals the mechanistic origin of NH_3_ inhibition during standard SCR over Cu-CHA catalysts and identifies practical strategies for its mitigation. We demonstrate that NH_3_ inhibition arises primarily from suppression of the oxidation half-cycle (OHC), while the reduction half-cycle (RHC) remains largely unaffected. Kinetic modeling of both steady-state and transient experiments shows that excess NH_3_ significantly reduces the pre-exponential factor for OHC, indicating a strong entropic or diffusional barrier under NH_3_-rich conditions. DFT calculations confirm that additional ligated or free NH_3_ increases the diffusion barrier for [Cu^I^(NH_3_)_2_]⁺ complexes, hindering the formation of Cu-oxo dimers essential for O_2_ activation. Operando EPR spectroscopy reveals a direct correlation between NH_3_ coordination on Cu sites and SCR activity, with excessive NH_3_ solvation restricting Cu mobility and shifting the redox balance toward Cu^I^.

Together, these findings provide a comprehensive molecular picture of NH_3_ inhibition and highlight actionable pathways to mitigate its effects – namely, by optimizing Cu content, enhancing framework design to promote Cu mobility, controlling operating temperatures, and employing advanced NH_3_ dosing strategies such as segmented injection and feedback control. These insights are particularly relevant for low-temperature applications and aged catalysts, offering a foundation for robust and durable SCR systems in future lean-burn and hybrid vehicles.

## Methods

### Catalyst preparation

Three Cu-SSZ-13 catalysts with varying Cu content were prepared using the following steps: (1) hydrothermal synthesis of Na-SSZ-13 zeolite support with Si/Al ratio of 12 and (2) series of ion-exchange processes (Na-SSZ-13 to NH_4_^+^-SSZ-13 and NH_4_^+^-SSZ-13 to Cu-SSZ-13).

To synthesize Na-SSZ-13 with Si/Al ratio of ~12, 0.8 g of NaOH (Sigma-Aldrich) was dissolved in 38 g of deionized water. While stirring the aqueous solution, 17 g of N,N,N-trimethyl-1- adamantyl ammonium hydroxide (TMAda-OH, Sachem Inc., 25 wt% in H_2_O) was added. Then, under constant stirring, 1.6 g of Al(OH)_3_ (Aldrich, containing ~54% Al_2_O_3_) was slowly added to form a homogeneous mixture. After the mixing, 40 g of colloidal SiO_2_ (AS-30, Sigma-Aldrich, 30 wt% suspension in H_2_O) was added slowly into the mixture and left under constant stirring for 2 h. The resulting mixture was transferred to a 125 mL Teflon-lined stainless-steel autoclave with a magnetic stirrer. The hydrothermal synthesis was carried out in a sand bath at 165 °C for 96 h under constant stirring. After the synthesis, the resulting white solid zeolite was recovered via centrifugation, followed by a three-time washing with DI water and subsequent centrifugation. The solid powder obtained this way was dried overnight in a vacuum oven at 70 °C and subsequently calcined under stagnant air at 650 °C for 5 h. The Si/Al ratio of ~12 was confirmed via inductively coupled plasma atomic emission spectroscopy (ICP-AES) performed at Galbraith Laboratories, Knoxville, Tennessee (Supplementary Table 1).

The parent Na-SSZ-13 zeolite support was first transformed into NH_4_^+^-SSZ-13 via two-time aqueous phase ion exchange with 0.1 M NH_4_NO_3_ (Sigma-Aldrich) solution at 80 °C for 2 h under constant stirring. The solid was recovered via three-time centrifugation and washing with DI water. Then NH_4_^+^-SSZ-13 was transformed into Cu-SSZ-13 with three different Cu loadings of 0.48 %, 1.39 % and 2.48 % (termed as Cu-0.5, Cu-1.4 and Cu-2.5, respectively) via aqueous ion exchange with Cu(NO_3_)_2_·2.5H_2_O (Sigma-Aldrich) solution at 80 °C for 2 h under constant stirring. The variation in Cu wt% was achieved by the following variations in the ion exchange procedure: one-time exchange with 0.02 M Cu^2+^ solution for Cu-0.5, two-time exchange with 0.02 M Cu^2+^ for Cu-1.4, and one-time exchange with 0.1 M Cu^2+^ for Cu-2.5. The obtained Cu-SSZ-13 solid was recovered with three-time centrifugation and washing with DI water, which was then dried overnight in a vacuum oven at 70 °C and subsequently calcined under stagnant air at 550 °C for 5 h.

On top of lab-synthesized Cu-SSZ-13 catalysts, commercial degreened and real-world field-aged (used for 710,000 miles) Cu-SSZ-13 catalysts provided by Cummins Inc. were investigated in this study. Details of all samples, including lab-synthesized and commercial catalysts, are provided in Supplementary Table 1.

### Catalyst testing

All catalytic tests were performed in a fixed-bed reactor system composed of a vertically mounted quartz tube, a K-type thermocouple placed directly above the catalyst bed, a tubular furnace (Applied Test Systems) with a PID controller (Omega CN3251), a set of Brooks 5850E series mass flow controllers, a Perma Pure MH™-Series humidifier (MH-110-12-S-4) for supplying H_2_O, and a MultiGas™ 2030 FTIR gas analyzer (MKS Instrument). Typically, ~100 mg of catalyst powder (sieved in 40–60 mesh) was loaded into a quartz tube. In standard steady-state SCR with varying ANR, the simulated exhaust feed was composed of 350 ppm NO, varying NH_3_, 10% O_2_, and 3% H_2_O in N_2_ balance with GHSV of ~150,000/h. NOx conversion was calculated using the following equation:

2 $${{{\rm{NOx}}}}\; {{{\rm{Conversion}}}}(\%)=\frac{{\left({{{\rm{NO}}}}+{{{{\rm{NO}}}}}_{2}\right)}_{{{{\rm{inlet}}}}}-\,{\left({{{\rm{NO}}}}+{{{{\rm{NO}}}}}_{2}\right)}_{{{{\rm{outlet}}}}}}{{\left({{{\rm{NO}}}}+{{{{\rm{NO}}}}}_{2}\right)}_{{{{\rm{inlet}}}}}}{{{\rm{x}}}}100$$

Cu oxidation and reduction kinetic parameters on Cu-0.5 were estimated using two different methods: (1) Langmuirian regression of standard SCR as a function of O_2_ partial pressure introduced by Krishna and coworkers^19,32^, and (2) transient response methodology used by Deka et al.^33^ and Nasello et al.^34^. For Langmuirian regression method, steady state SCR rate was measured for ANR of 0.1, 0.2, 0.5, 1.0, and 1.25 with varying O_2_ partial pressure from 0 kPa to 187 kPa at 200 °C. For the transient response method, oxidized Cu-0.5 was first saturated under 175 ppm (for ANR of 0.5) or 437 ppm (for ANR of 1.25) NH_3_, 10% O_2_, and 3% H_2_O in N_2_ balance at 150, 175, and 200 °C. After the saturation, O_2_ was removed and subsequently the RHC was performed by introducing 350 ppm NO while constantly flowing NH_3_ and 3% H_2_O in N_2_ balance. This allowed the measurement of RHC NO transient under two different ANRs and three different temperatures. After the RHC, 10% O_2_ was introduced for creating standard SCR environment. RHC was performed once more at the end of standard SCR. The RHC and SCR transients were fitted with kinetic models as described in Supplementary Note 3.

### Density functional theory computational setup

DFT calculations were performed using the Vienna Ab-initio Simulation Package (VASP) package (version 5.4.4)^45,46^. The Perdew–Burke–Ernzerhof (PBE) functional^47^ and projected augmented wave (PAW) potentials were adopted for all the calculations. The convergence criteria of energy and force are 0.03 eV/Å and 10^−4^ eV for relaxing structures, respectively. A Monkhorst–Pack mesh^48^ k-point grids of (3 × 3 × 3) and an energy cutoff for the valence plane waves of 400 eV were used in this work.

To measure the diffusion barrier of Cu ions with NH_3_, we built a dual-rhombohedral chabazite unit cell with *a* = 18.72 Å, *b* = 9.48 Å, and *c* = 9.36 Å, including 24 tetrahedral sites (22 Si, 2 Al) and 48 O atoms, where the Si/Al ratio was 11. Initially, two Cu ions with NH_3_ were placed in two CHA cages separately, with a Cu-Cu distance of ~9 Å. A Cu ion, coordinated with NH_3_, migrated through the 8-MR window and settled in the same cage as the other Cu ion, with a Cu-Cu distance of approximately 4.5 Å. We captured 9 images throughout the migration process. The image with the highest energy was identified as the transition state, and the energy difference between this transition state and the initial state was used to determine the diffusion barrier. While calculating the energy barriers, all DFT energies were corrected by including zero-point energy (ZPE), internal energy (∆*U*) and entropy (*S*) contribution, and the Gibbs free energy (*G*) was obtained by *G* = *E*_DFT_ + ZPE + ∆*U* – TS. The thermodynamic corrections were performed using the VASPKIT package^49^. The climbing image nudged elastic band (CI-NEB) and dimer methods were used to search for the transition state of O_2_ binding and O-O activation, which was further verified by calculating the vibrational frequencies, confirming that a single imaginary frequency was obtained.

### NH_3_-temperature programmed desorption (NH_3_-TPD)

NH_3_-TPD was performed on ~100 mg Cu-0.5 sample in the same fixed-bed reactor system used for the catalytic tests. For NH_3_-TPD on reduced Cu-SSZ-13 catalyst, the catalyst was saturated with NH_3_ under a gas flow of 350 ppm NH_3_ and 350 ppm NO in N_2_ balance at 180 °C. The system was then purged under constant N_2_ flow for 3 h. Subsequently, TPD was performed under the same N_2_ flow with 2 °C/min ramp to 550 °C. For NH_3_-TPD on oxidized Cu-SSZ-13 catalyst, NH_3_ saturation was first performed under 350 ppm NH_3_ and 10% O_2_ in N_2_ balance at 180 °C. Then, the system was purged, and TPD was performed as described above. NH_3_-TPD on H-SSZ-13 with Si/Al ratio of 12 was performed by first saturating H-SSZ-13 under 350 ppm NH_3_ in balance N_2_ at 180 °C, then purging the system under constant N_2_ flow, and subsequently performing desorption with 2 °C/min ramp to 550 °C. Additional details can be found in Supplementary Note 5.

### *Operando* electron paramagnetic resonance (EPR)

EPR spectra were collected with a Bruker 580 Elexsys equipped with a SHQE resonator. The sample temperature was maintained with a Bruker ER4131VT nitrogen temperature control system. Typically, the microwave frequency was 9.3 GHz at 0.2 mW power. The field was swept 1200 G in 84 s with 5 G field modulation and 82 ms of time constant. For ex situ measurements, the samples were contained in 4 mm OD quartz tubes (Wilmad). The EPR sample holder used for operando experiments was a coaxial plug flow reactor consisting of an inner 3 mm tube that contained the sample (~15 mg) between plugs of quartz wool. An outer 5 mm tube carried the gas in a cross-flow manner to preheat the reactant gases to the desired temperature before contacting the sample. Total gas flow was 100 ml/min, with the mixture controlled by four Brooks computer-controlled flow meters. Feed gases (raised to the desired temperature by an electrically heated coil) enter through the outer tube, and then flow through the aforementioned pinhole and leave the system through the open end of the sample holder (which is connected and sealed with a custom-built adapter). Around 20–30 mg of Cu-0.5 samples was loaded into the inner tube, supported with quartz wool at the top and bottom, and reactant gases flow through this bed. EPR spectra were collected under steady-state SCR at 100–350 °C temperature range and ANR of 0.125–2.0 with 500 ppm NO, 500 ppm NH_3_, 10% O_2_, and 3% H_2_O with a space velocity of ~400,000/h. The EPR spectra were collected throughout the experiment. Changes in ANRs and temperatures were made only when three stable subsequent spectra were obtained at a specific ANR and temperature combination.

## Supplementary information

Supplementary Information

Transparent Peer Review file

## Data Availability

Data are available from the corresponding authors upon request.
