# Supplementary material for: Insights into the mechanisms of NH3 inhibition on Cu-CHA SCR catalysts
Source: Nat Commun. 2026 Jul 28;17:7421. doi: 10.1038/s41467-026-72879-7 (PMC13408143; doi:10.1038/s41467-026-72879-7)
Supplement: Supplementary file 1 — Supplementary Information [file 41467_2026_72879_MOESM1_ESM.pdf]

SUPPLEMENTARY INFORMATION FILE

Insights into the mechanisms of NH_3 inhibition on Cu-CHA SCR catalysts

Dhruba J. Deka [1, a, #], Mingyu Wan [2, a], Garam Lee [1], Eric Walter [1], Fanglin Che [3, $], Kenneth G. Rappe [1], János Szanyi [1], Yong Wang [1,4, *]

[1] *Institute of Integrated Catalysis, Pacific Northwest National Laboratory, Richland, WA 99354, USA*

[2] *Department of Chemical Engineering, University of Massachusetts Lowell, Lowell, MA 01854, USA*

[3] *Department of Chemical Engineering, Worcester Polytechnic Institute, Worcester, MA 01609, USA*

[4] *The Gene and Linda Voiland School of Chemical Engineering and Bioengineering, Washington State University, Pullman, WA 99163, USA*

* Corresponding author: wang42@wsu.edu

[#] Co-corresponding author: dhrubajyoti.deka@pnnl.gov

[$] Co-corresponding author: fche@wpi.edu

[a] These authors contributed equally to the work

SUPPLEMENTARY METHODS:

Catalyst preparation:

Three Cu-SSZ-13 catalysts with varying Cu content were prepared using following steps: (1) hydrothermal synthesis of Na-SSZ-13 zeolite support with Si/Al ratio of 12 and (2) series of ion-exchange processes (Na-SSZ-13 to NH_4^+-SSZ-13 and NH_4^+-SSZ-13 to Cu-SSZ-13).

To synthesize Na-SSZ-13 with Si/Al ratio of ~12, 0.8 g of NaOH (Sigma-Aldrich) was dissolved in 38 g of deionized water. While stirring the aqueous solution, 17 g of N,N,N-trimethyl-1- adamantyl ammonium hydroxide (TMAda-OH, Sachem Inc., 25 wt% in H_2O) was added. Then, under constant stirring, 1.6 g of $Al(OH)_3$ (Aldrich, containing ~54% Al_2O_3) was slowly added to form a homogeneous mixture. After the mixing, 40 g of colloidal SiO_2 (AS-30, Sigma-Aldrich, 30 wt% suspension in H_2O) was added slowly into the mixture and left under constant stirring for 2 hours. The resulting mixture was transferred to a 125 mL Teflon-lined stainless-steel autoclave with a magnetic stirrer. The hydrothermal synthesis was carried out in a sand bath at 165 °C for 96 hours under constant stirring. After the synthesis, the resulting white solid zeolite was recovered via centrifugation followed by a three-time washing with DI water and subsequent centrifugation. The solid powder obtained this way was dried overnight in a vacuum oven at 70 °C and subsequently calcined under stagnant air at 650 °C for 5 hours. The Si/Al ratio of ~12 was confirmed via inductively coupled plasma atomic emission spectroscopy (ICP-AES) performed at Galbraith Laboratories, Knoxville, Tennessee (**Supplementary Table 1**).

The parent Na-SSZ-13 zeolite support was first transformed into NH_4^+-SSZ-13 via two-time aqueous phase ion exchange with 0.1 M NH_4NO_3 (Sigma-Aldrich) solution at 80 °C for 2 hours under constant stirring. The solid was recovered via three-time centrifugation and washing with DI water. Then NH_4^+-SSZ-13 was transformed into Cu-SSZ-13 with three different Cu loadings of 0.48 %, 1.39 % and 2.48 % (termed as Cu-0.5, Cu-1.4 and Cu-2.5, respectively) via aqueous ion exchange with $Cu(NO_3)_2 \cdot 2.5H_2O$ (Sigma-Aldrich) solution at 80 °C for 2 hours under constant stirring. The variation in

Cu wt% was achieved by the following variations in ion exchange procedure: one time exchange with 0.02 M Cu^{2+} solution for Cu-0.5, two-time exchange with 0.02 M Cu^{2+} for Cu-1.4 and one time exchange with 0.1 M Cu^{2+} for Cu-2.5. The obtained Cu-SSZ-13 solid was recovered with three-time centrifugation and washing with DI water, which was then dried overnight in a vacuum oven at 70 °C and subsequently calcined under stagnant air at 550 °C for 5 hours.

On top of lab-synthesized Cu-SSZ-13 catalysts, commercial degreened and real-world field aged (used for 710,000 miles) Cu-SSZ-13 catalysts provided by Cummins Inc. were investigated in this study. Details of all samples including lab-synthesized and commercial catalysts are provided in **Table 1** in the supplementary information (SI).

Catalyst testing:

All catalytic tests were performed in a fixed-bed reactor system composed of a vertically mounted quartz tube, a K-type thermocouple placed directly above the catalyst bed, a tubular furnace (Applied Test Systems) with a PID controller (Omega CN3251), a set of Brooks 5850E series mass flow controllers, a Perma Pure MH™-Series humidifier (MH-110-12-S-4) for supplying H_2O, and a MultiGas™ 2030 FTIR gas analyzer (MKS Instrument). Typically, ~100 mg of catalyst powder (sieved in 40-60 mesh) was loaded into a quartz tube. In standard steady-state SCR with varying ANR, the simulated exhaust feed was composed of 350 ppm NO, varying NH_3, 10% O_2, and 3% H_2O in N_2 balance with GHSV of ~150,000/h. NOx conversion was calculated using the following equation:

$$\text{NOx Conversion (\%)} = \frac{(\text{NO+NO}_2)_{\text{inlet}} - (\text{NO+NO}_2)_{\text{outlet}}}{(\text{NO+NO}_2)_{\text{inlet}}} \text{ x } 100 \quad (1)$$

Cu oxidation and reduction kinetic parameters on Cu-0.5 were estimated using two different methods: (1) Langmuirian regression of standard SCR as a function of O_2 partial pressure introduced by Krishna and coworkers,[1, 2] and (2) transient response methodology used by Deka et al.[3] and Nasello et al.[4] For Langmuirian regression method, steady state SCR rate was measured for ANR of 0.1, 0.2, 0.5, 1.0,

and 1.25 with varying O_2 partial pressure from 0 kPa to 187 kPa at 200 °C. For transient response method, oxidized Cu-0.5 was first saturated under 175 ppm (for ANR of 0.5) or 437 ppm (for ANR of 1.25) NH_3, 10% O_2, and 3% H_2O in N_2 balance at 150, 175, and 200 °C. After the saturation, O_2 was removed and subsequently the RHC was performed by introducing 350 ppm NO while constantly flowing NH_3 and 3% H_2O in N_2 balance. This allowed the measurement of RHC NO transient under two different ANRs and three different temperatures. After the RHC, 10% O_2 was introduced for creating standard SCR environment. RHC was performed once more at the end of standard SCR. The RHC and SCR transients were fitted with kinetic models as described in **Note 3**.

Density functional theory computational setup:

DFT calculations were performed using the Vienna Ab-initio Simulation Package (VASP) package (version 5.4.4).[5,6] The Perdew-Burke-Ernzerhof (PBE) functional[7] and projected augmented wave (PAW) potentials were adopted for all the calculations. The convergence criteria of energy and force are 0.03 eV/Å and 10^{-4} eV for relaxing structures, respectively. A Monkhorst-Pack mesh[8] k-point grids of (3×3×3) and an energy cutoff for the valence plane waves of 400 eV were used in this work.

To measure the diffusion barrier of Cu ions with NH_3, we built a dual-rhombohedral chabazite unit cell with a = 18.72 Å, b = 9.48 Å, and c = 9.36 Å, including 24 tetrahedral sites (22 Si, 2 Al) and 48 O atoms, where the Si/Al ratio was 11. Initially, two Cu ions with NH_3 were placed in two CHA cages separately, with a Cu-Cu distance of ~9 Å. A Cu ion, coordinated with NH_3, migrated through the 8-MR window and settled in the same cage as the other Cu ion, with a Cu-Cu distance of approximately 4.5 Å. We captured 9 images throughout the migration process. The image with the highest energy was identified as the transition state, and the energy difference between this transition state and the initial state was used to determine the diffusion barrier. While calculating the energy barriers, all DFT energies were corrected by including zero-point energy (ZPE), internal energy (ΔU) and entropy (S) contribution, and the Gibbs free energy (G) was obtained by $G = E_{DFT} + ZPE + \Delta U - TS$. The thermodynamic corrections were

performed using the VASPKIT package [9] . The climbing image nudged elastic band (CI-NEB) and dimer methods were used to search for the transition state of O_2 binding and O-O activation, which was further verified by calculating the vibrational frequencies confirming that a single imaginary frequency was obtained.

NH_3-temperature programmed desorption (NH_3-TPD):

NH_3-TPD was performed on ~100 mg Cu-0.5 sample in the same fixed-bed reactor system used for the catalytic tests. For NH_3-TPD on reduced Cu-SSZ-13 catalyst, the catalyst was saturated with NH_3 under a gas flow of 350 ppm NH_3 and 350 ppm NO in N_2 balance at 180 °C. The system was then purged under constant N_2 flow for 3 hours. Subsequently, TPD was performed under the same N_2 flow with 2 °C/min ramp to 550 °C. For NH_3-TPD on oxidized Cu-SSZ-13 catalyst, NH_3 saturation was first performed under 350 ppm NH_3 and 10% O_2 in N_2 balance at 180 °C. Then, the system was purged and TPD was performed as described above. NH_3-TPD on H-SSZ-13 with Si/Al ratio of 12 was performed by first saturating H-SSZ-13 under 350 ppm NH_3 in balance N_2 at 180 °C, then purging the system under constant N_2 flow, and subsequently performing desorption with 2 °C/min ramp to 550 °C. Additional details can be found in **Note 5**.

***Operando* electron paramagnetic resonance (EPR):**

EPR spectra were collected with a Bruker 580 Elexsys equipped with a SHQE resonator. The sample temperature was maintained with a Bruker ER4131VT nitrogen temperature control system. Typically, the microwave frequency was 9.3 GHz at 0.2 mW power. The field was swept 1200 G in 84 seconds with 5 G field modulation and 82 ms of time constant. For ex situ measurements, the samples were contained in 4 mm OD quartz tubes (Wilmad). The EPR sample holder used for operando experiments was a coaxial plug flow reactor consisting of an inner 3 mm tube that contained the sample (~15 mg) between plugs of quartz wool. An outer 5 mm tube carried the gas in a cross-flow manner to preheat the reactant gases to the desired temperature before contacting the sample. Total gas flow was 100

ml/minute, with the mixture controlled by four Brooks computer controlled flow meters. Feed gases (raised to the desired temperature by an electrically heated coil) enter through the outer tube, and then flow through the aforementioned pinhole and leaves the system through the open end of the sample holder (which is connected and sealed with a custom-built adapter). Around 20-30 mg of Cu-0.5 samples was loaded into the inner tube, supported with quartz wool at the top and bottom, and reactant gases flow through this bed. EPR spectra were collected under steady-state SCR at 100-350 °C temperature range and ANR of 0.125-2.0 with 500 ppm NO, 500 ppm NH_3, 10% O_2, and 3% H_2O with space velocity of ~400,000/hr. The EPR spectra was collected throughout the experiment. Changes in ANRs and temperatures were made only when three stable subsequent spectra were obtained at a specific ANR and temperature combination.

Supplementary Table 1. Elemental composition of Cu-SSZ-13 catalysts

Sample Id	Si content (wt%)	Al content (wt%)	Si/Al ratio	Cu content (wt%) [a]	Cu/Al ratio	Cu-ion per CHA cage
Cu-0.5	39.1	3.17	11.9	0.47	0.06	0.05
Cu-1.4				1.39	0.19	0.15
Cu-2.5				2.48	0.33	0.30
CMI-DG *	--	--	12	2.31	0.30	0.05
CMI-FA **	--	--	12	2.42 (1.39)	0.30 (0.18)	0.29 (0.17)

[a] Values within parenthesis show isolated Cu content measured by EPR (1.39 wt% Cu in this case) and Cu/Al ratio and Cu-ion per cage based on such isolated Cu content

* CMI-DG: Degreened Cu-SSZ-13 from Cummins

** CMI-FA: Field aged Cu-SSZ-13 from Cummins (exposed to 710,000 miles in a real-world vehicle)

Note 1: Standard SCR on commercial degreened and field aged catalysts:

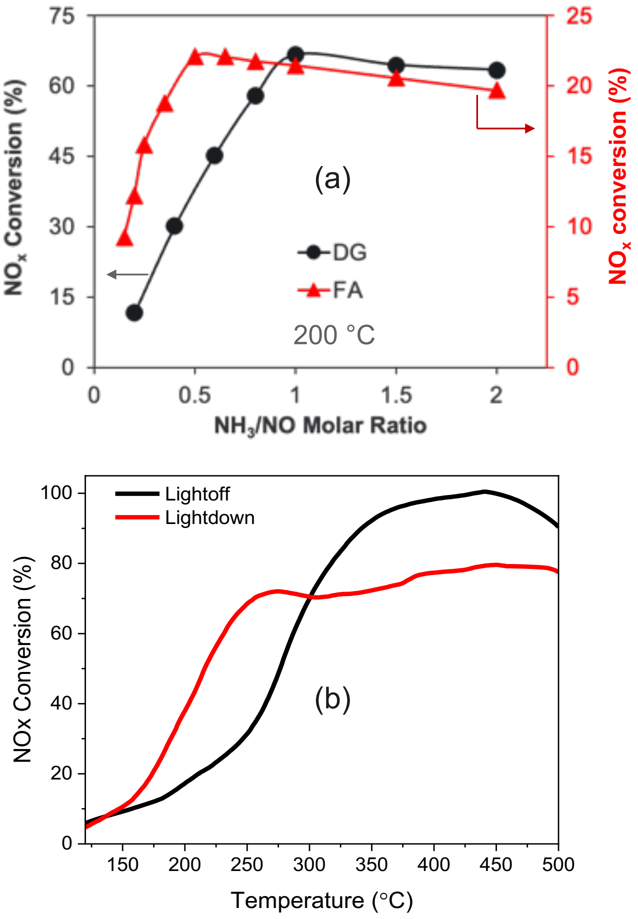

Supplementary Figure 1 | SCR performance: ANR and temperature effects (fresh vs aged catalysts). (a) Standard SCR NO_x conversion at a steady state temperature of 200 °C and ANR's varying between 0.2 to 2.0 on degreened (DG) and field aged (FA) samples (sample details in Supplementary Table 1). NO_x conversion on DG is plotted along primary y-axis, and that for FA is plotted along secondary y-axis. (b) SCR light-off and light-down NO_x conversion on FA catalyst measured under temperature ramp with 20 °C/min. All experiments use feed composition of 350 ppm NO, 10% O_2, 3% H_2O, ~150,000/h GHSV with varying NH_3 to deliver a desired NH_3/NO ratio (ANR). Light-off/Light-down test had a feed ANR of 1.0.

Figure 1a demonstrates that both DG and FA catalysts exhibit NH_3-inhiition above critical NH_3/NO values of 1.0 and 0.5, respectively. The NO_x conversion light-off and light-down curves collected under transient temperature (Fig. 1b) show two key differences: (1) light-off is higher than light-down at temperatures > 300 °C, (2) light-off is lower than light-down at <300 °C. This can be explained by considering the NH_3 inventory of the catalyst. When light-off temperature ramp begins, the catalyst contains a significant amount of adsorbed NH_3 which creates an additional reservoir of NH_3 along with the NH_3 present in reactor feed. That is why the NO_x conversion reaches as high as 100% at >400 °C. At higher temperatures, however, the adsorbed NH_3 reservoir is exhausted due to weak NH_3 binding at such high temperatures as well as the NH_3 oxidation side reaction, which leads to the observed decrease in NO_x conversion. Due to the same reason, the catalyst sees less NH_3 at temperatures below 300 °C under light-down conditions than light-off, and yet the NO_x conversion is higher under light-down than light-off. The high effective NH_3/NO ratio during light-off leads to NH_3-inhibition of the SCR reaction, resulting in a lower conversion than light-down. Both steady-state and transient tests thus show NH_3 inhibition of SCR on commercial catalysts, with a higher extent of inhibition observed in the field-aged sample.

Note 2: NH_3-inhibtion on Cu-2.4 during transient SCR:

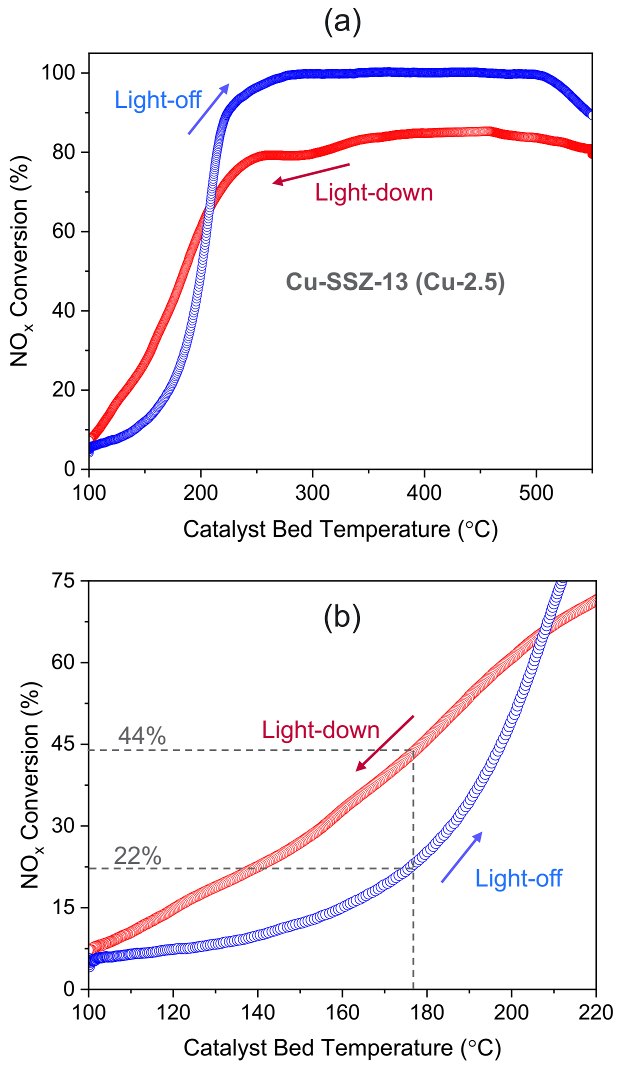

Supplementary Figure 2 | SCR light-off/down behavior and NH_3 inhibition. SCR light-off and light-down NO_x conversion on Cu-2.4 catalyst measured under temperature ramp with 20 °C/min. Feed composition: 350 ppm NO, 350 ppm NH_3, 10% O_2, 3% H_2O, ~150,000/h GHSV. Panel (a) shows the data between 100 °C-525 °C, while panel (b) shows at an enlarged scale of 100 °C-220 °C for better visual comparison of low-temperature region. The light-off and light-down curves on Cu-2.5 show similar behavior as explained for Fig. 1b. In the low-temperature range, the NOx conversion during light-down could decrease to almost half during light-off (44% NOx conversion to 22% at 178 °C) owing to NH_3-inhibition in the light-off curve.

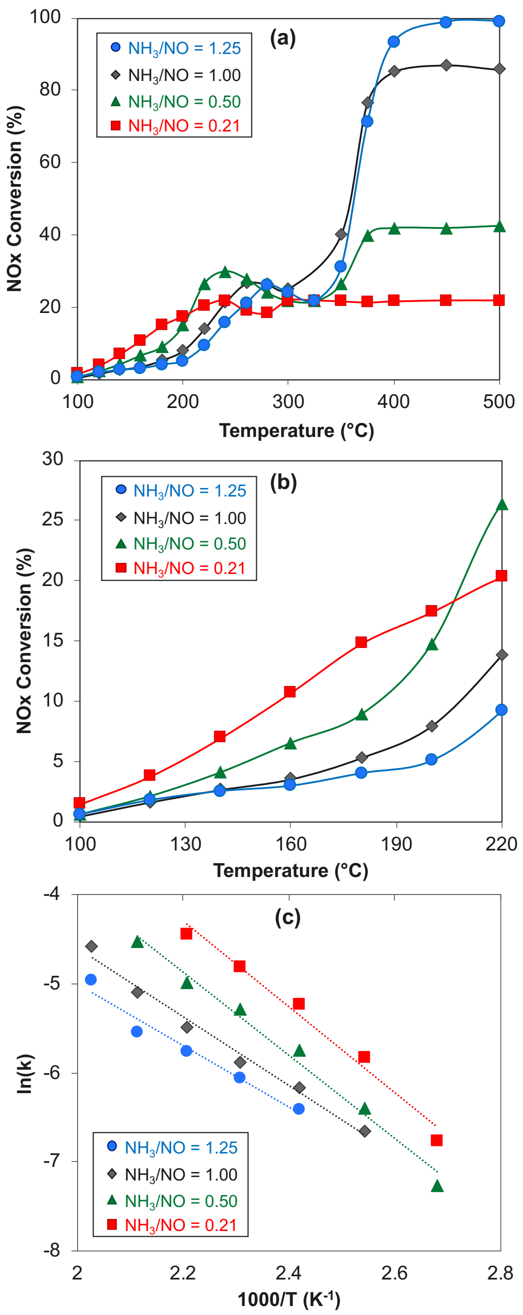

Supplementary Figure 3 | SCR activity and kinetics vs. ANR. (a) Standard SCR NO_x conversion on Cu-0.5 in the temperature range 100 °C-500 °C across four different NH_3/NO_x molar ratios: 0.21, 0.50, 1.00 and 1.25, (b) the same data in the low-temperature range of 100 °C-220 °C to allow a better visual in this range, (c) Arrhenius plot for data shown in panels (a) and (b). Feed composition: 350 ppm NO, 350 ppm NH_3, 10% O_2, 3% H_2O, ~150,000/h GHSV.

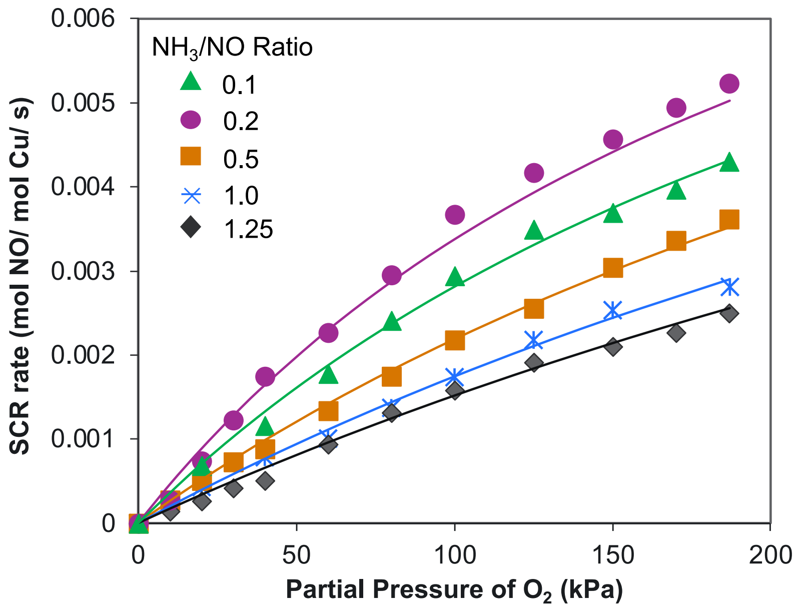

Supplementary Figure 4 | SCR rates as a function of O_2 partial pressure at varying ANRs: Steady state SCR rates (normalized over per Cu site) at 200 °C as a function of O_2 pressure across five different NH_3/NO molar ratios: 0.1, 0.2, 0.5, 1.0 and 1.25. All experiments use feed composition of 350 ppm NO, 3% H_2O, ~150,000/h GHSV, varying O_2 and with varying NH_3 to deliver a desired ANR.

Note 3: O_2-pulse experiments to measure Cu(I) oxidation kinetics

Cu(I) oxidation measurements were performed to isolate the elementary Cu(I) oxidation kinetics based on its 2nd order dependence on Cu. In these experiments (**Supplementary Fig. 5a**), the entire Cu inventory was first reduced to Cu(I) by exposing the catalyst to NO+NH_3 at 200 °C, followed by exposure to O_2 for controlled time periods to oxidize a finite fraction of Cu(I) to Cu(II), and then re-reduced to Cu(I) with NO+NH_3. The amount of NO consumed in these NO+NH_3 titrations are then calculated to quantify the amount of Cu(II) formed during each discrete oxidation period. **Supplementary Figure 5b** presents such Cu(II) fraction formation as a function of time. These measurements were performed at ANRs 0.5 and 1.25, as the Cu(II) formation rate was higher in the former case than the latter as evident from, **Supplementary Fig. 5b**, indicating a higher Cu(I) oxidation rate at the lower ANR. Such Cu(II) oxidation rate are then fitted to the following rate expression depicting 2nd order dependence on Cu(I):

$$R_{OHC} = k_{OHC} \cdot [O_2] \cdot [Cu(I)]^2 \qquad (2)$$

Since O_2 is in large excess, [O_2] can be considered a constant. The Cu(I) oxidation rate vs. [Cu(I) fraction]^2 plot is linear as seen in **Supplementary Fig. 5c**, confirming the Cu(I) reaction order as 2. The rate constants calculated from the plot are 1.35×10^{-2} s^{-1} at ANR = 0.5, and 1.15×10^{-2} s^{-1} at ANR = 1.25, consistent with the lower oxidation rate at higher the ANR.

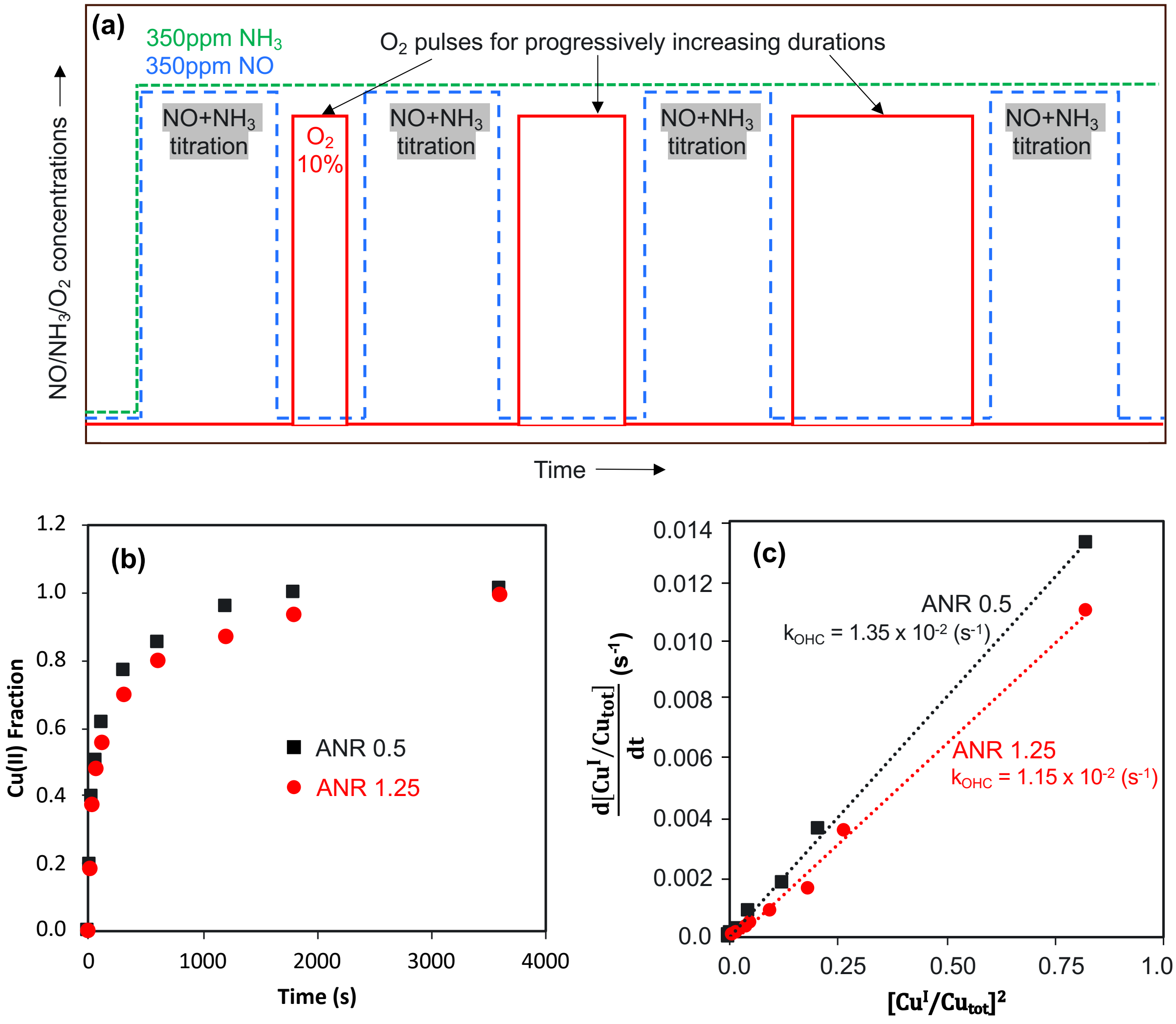

Supplementary Figure 5 | O_2 pulse protocol and second-order Cu^I oxidation kinetics. (a) O_2-pulse experimental protocol to probe Cu^I oxidation rate: Initially, 350 ppm NO + 350 ppm NH_3 is flowed over the catalyst (Cu-0.5) to reduce all Cu sites to Cu^I. NO is then turned off and O_2 is introduced for a designated length of time, following which O_2 is turned off. NO and NH_3 is restarted to reduce the Cu^{II} sites formed by O_2-derived oxidation of Cu^I sites, and by calculating the NO consumed at the step provides the number of Cu^I sites oxidized by O_2 pulse. This process is repeated with various time durations of O_2 pulses. The Cu^{II} fraction vs. oxidation time calculated this way is plotted in panel (b) for two ANRs: 0.5 and 1.25. Panel (c) plots the consumption rate of Cu(I) by O_2-derived oxidation vs. the square of Cu^I fraction. A linear relationship is obtained indicating a second order dependence of Cu(I) oxidation rate on Cu^I concentration.

Note 4: Transient RHC and SCR protocol and associated kinetic model

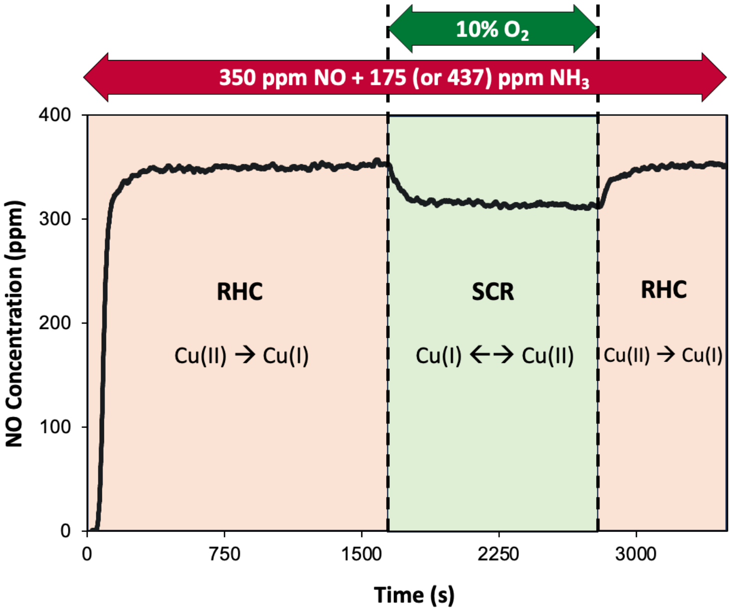

Supplementary Figure 6 | RHC-SCR transient protocol under varying ANRs. RHC-SCR transient protocol begins with NO flow over NH_3-saturated oxidized Cu-0.5. The concentration of NH_3 was either 175 ppm or 437 ppm to vary the NH_3/NO molar ratio to 0.5 or 1.25, respectively. The SCR step includes 10% O_2 flow along with NO and NH_3 flow. All experiments were done under 3% H_2O, balance N_2 and 150,000/h space velocity.

A kinetic model that describes the RHC NO transient was developed by assuming the catalyst bed used in our experiments as 20 continuous stirred tank reactors (CSTR) in series at isothermal and isobaric condition, similar to the method used by Nasello et al.[4] The mass balance of each CSTR is expressed as:

NO mass balance: $$\frac{dN_{NO}}{dt} = (F_{NO}^{0} - F_{NO}) - Cu_{Total} \cdot r_{RHC} \quad (3)$$

$$\Rightarrow \boldsymbol{\tau}_R \frac{dy_{NO}}{dt} = (y_{NO}^{0} - y_{NO}) - \boldsymbol{\tau}_{cat} \cdot r_{RHC} \quad (4)$$

Cu^{II} balance: $$\frac{dx_{Cu^{II}}}{dt} = -r_{RHC} \quad (5)$$

Here, F_{NO}^{0} is NO molar flowrate entering a CSTR, F_{NO} is NO molar flowrate exiting a CSTR, y_{NO}^{0} is NO mole fraction in CSTR feed, y_{NO} is NO mole fraction in CSTR exit, $\boldsymbol{\tau}_R$ is the residence time in CSTR, $\boldsymbol{\tau}_{cat}$ is the contact time with catalyst expressed as N_{Cu}/F (mol Cu/mol feed/s) where N_{Cu} is total moles of Cu sites and F is total feed molar flowrate. In addition, r_{RHC} is the rate of reduction half cycle, and $x_{Cu^{II}}$ is the fraction of Cu^{II} sites.

The RHC reaction is expressed as:[3, 4]

$$ZCu^{II}OH + NO + NH_3 \longrightarrow ZCu^{I} + N_2 + H_2O \quad (6)$$

Associated RHC reaction rate law is assumed as:

$$r_{RHC} = r_{RHC}\left(\frac{y_{NO}}{y_{NO}^{0}}\right) x_{Cu^{II}} \text{ , where } x_{Cu^{II}} = \frac{N_{CuII}}{N_{CuTotal}} \quad (7)$$

The differential equations related to all CSTRs were solved simultaneously to find a fit to the overall RHC NO transient. The fit to the NO transient provides us a solution to the RHC rate constant (k_{RHC}). These experiments were run at three different temperatures (150 °C, 175 °C and 200 °C), and two different ANRs (0.5 and 1.25). Experimental data and model fits are presented in **Fig. 7**. An Arrhenius equation, $k = A\ exp\ (-E_a/RT)$ was used to estimate the RHC activation energy (E_a) and pre-exponential factor (A) for each ANR.

Following the RHC transient, O_2 was introduced into the system while NO and NH_3 flow continues, thus creating an SCR environment. As seen in **Fig. 6** and **Fig. 8**, NO concentration slowly decreases and achieves a steady state which also oxidizes a fraction of the Cu^{I} sites to create a steady state population of Cu^{I} and Cu^{II}. Another RHC transient was collected following this SCR steady state by stopping O_2 flow and allowing only NO and NH_3 to flow. The area under this post-SCR RHC NO transient provides an estimate of the total number of Cu^{I} sites that get oxidized to Cu^{II} (i.e. $x_{Cu^{II}}$) during steady state SCR. The OHC rate can be expressed as:

$$r_{OHC} = k_{OHC}\left(\frac{P_{O_2}}{P_{O_2}^{0}}\right)(1 - x_{Cu^{II}})^2 \quad (8)$$

Where is the OHC rate constant, P_{O_2} is the oxygen partial pressure, and $x_{Cu^{II}}$ is Cu^{II} fraction. The OHC rate is second order with respect to Cu^{II} sites, since oxygen activation occurs on dimeric Cu^{II}. Since we already know $x_{Cu^{II}}$ and r_{RHC} as described in the previous paragraphs, and $r_{OHC} = r_{RHC}$ at steady state SCR, k_{OHC} can be readily calculated. An Arrhenius analysis of k_{OHC} at different temperatures and ANRs provides an estimate of OHC activation energy and pre-exponential factor.

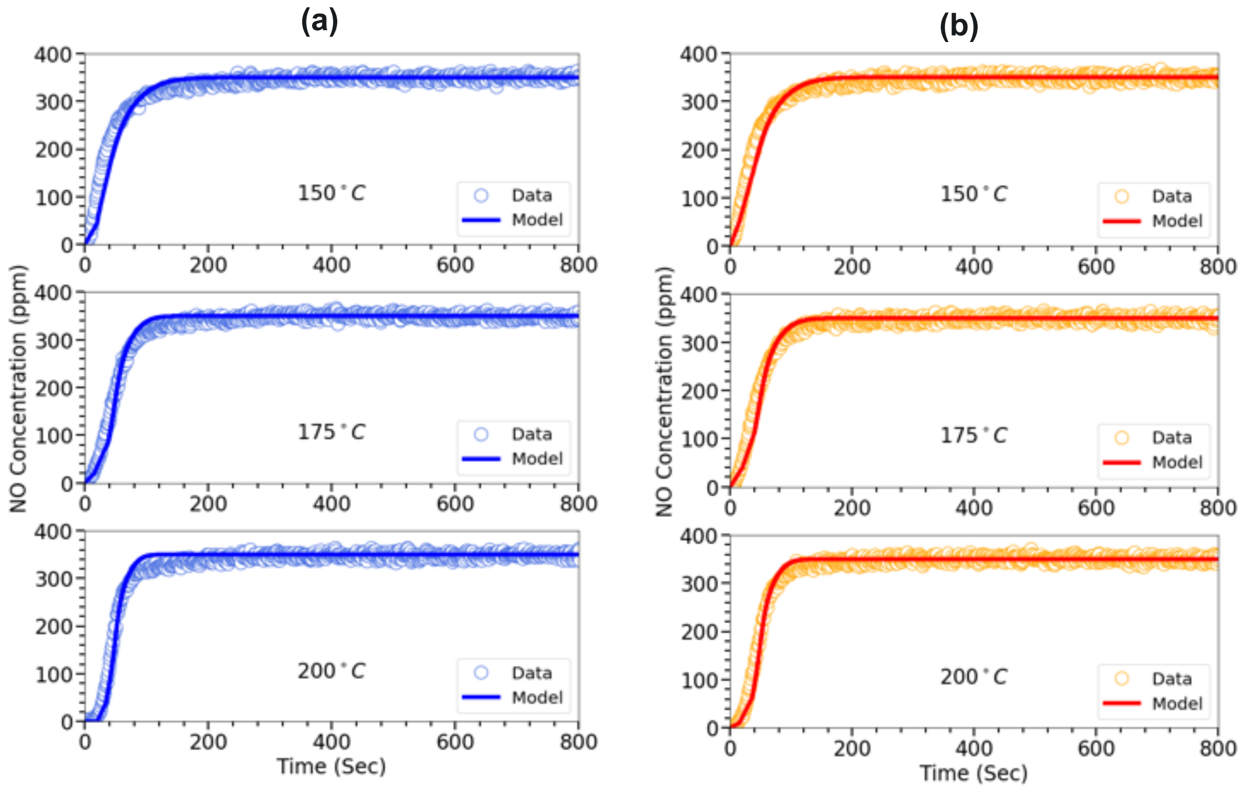

Supplementary Figure 7 | RHC NO transients and model fits. NO transient measured during RHC protocol at (a) NH_3/NO = 0.5 and (b) NH_3/NO = 1.25. circular symbols represent experimental data, and solid lines represent model fits. Each NH_3/NO ratio was replicated at three different temperatures: 150 °C, 175 °C and 200 °C.

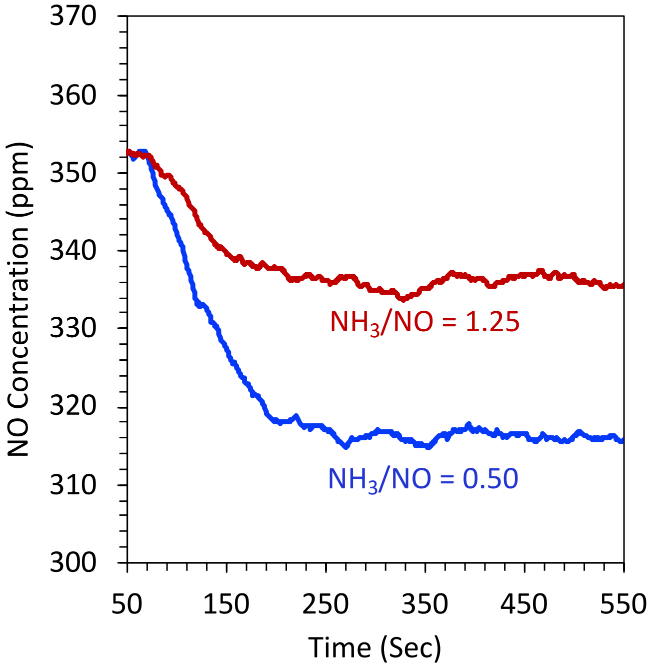

Supplementary Figure 8 | SCR NO transient and Cu^{I} oxidation quantification. (a) NO transient collected during the SCR step in RHC-SCR transient protocol. At the beginning of this SCR step, the catalyst is under reducing NO+NH_3 environment. Introduction of O_2 kicks off the SCR step, leading to the SCR NO transient seen in this figure. The data shown in these figures belong to ANR = 0.5 and 1.25 at 200 °C. The amount of Cu^{I} that was oxidized to Cu^{II} during the transient phase of the SCR step before it reached steady state is calculated based on the area under the subsequent RHC NO transient with post-RHC steady state NO as the baseline. At 200 °C, amount of Cu^{I} that oxidized to Cu^{II} was calculated to be 0.86 µmols and 0.69 µmols at ANR = 0.5 and 1.25, respectively. Similar experiments were also performed at 150 °C and 175 °C to facilitate OHC activation energy and pre-exponential factor calculations.

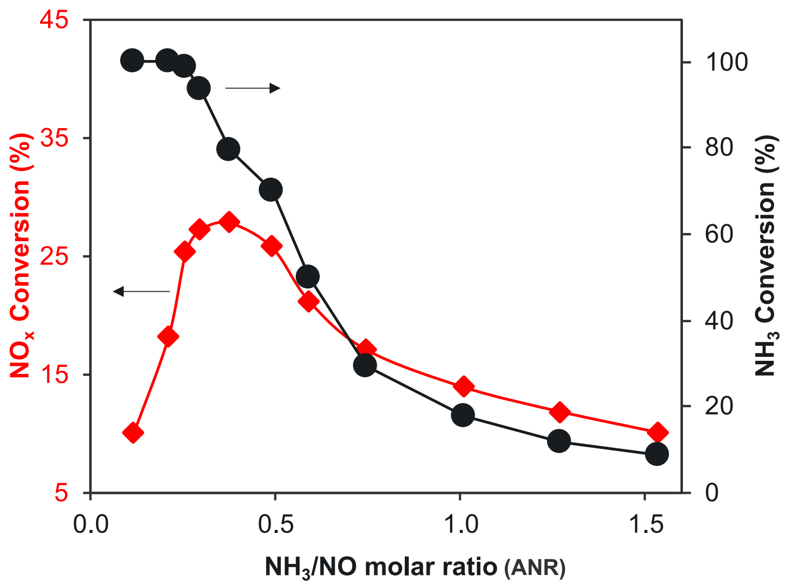

Supplementary Figure 9 | SCR NO_x and NH_3 conversion vs ANR. SCR NOx conversion (primary Y-axis) and corresponding NH_3 conversion (secondary Y-axis) as a function of ANR on Cu-0.5 sample at 220 °C. Feed composition: 350 ppm NO, 10% O_2, 3% H_2O, ~150,000/h GHSV with varying NH_3 to deliver a desired ANR.

Note 5: NH_3-temperature programmed desorption following steady state SCR

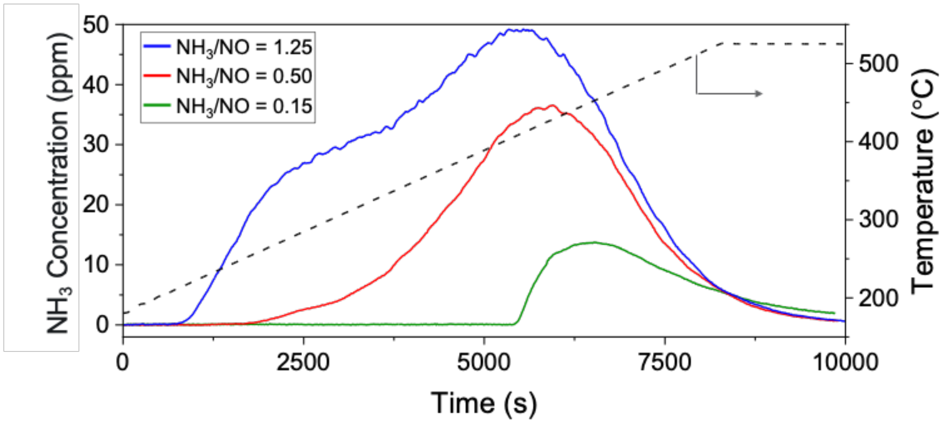

Supplementary Figure 10 | NH_3-TPD after SCR operation at 180 °C at varying ANRs. NH_3 TPD profiles obtained from Cu-0.5 following SCR at three different ANRs: 0.15, 0.5 and 1.25. In these experiments, SCR test was first performed at 180 °C until a steady state was reached, following which all reactants were stopped, and a 90 mins isothermal flushing was done under N_2 flow. Temperature ramp was then started to desorb NH_3 from the catalysts.

Two distinct peaks, one at low temperature and another high temperature, were observed for NH_3/NO of 1.25. The low-temperature peak is typically attributed to NH_3 adsorbed on Cu sites, while the high-temperature peak to Bronsted-bound NH_3.[10, 11] Intensity of both peaks decreases at lower NH_3/NO. In fact, the low-temperature peak completely vanished at NH_3/NO of 0.15. Since formation of mobile $[Cu^{I}(NH_3)_2]^+$ complexes is indispensable at low temperatures to drive the oxidation half reaction,[12, 13] no presence of Cu-bound NH_3 at NH_3/NO = 0.15 means the residence time of such species is very short, as the abundant NO molecules react with these NH_3 molecules. Nevertheless, such post-SCR NH_3-TPD profiles show that the presence of adsorbed NH_3 increases with increasing NH_3/NO molar ratio. Since we observed NH_3-inhibition on SCR activity above NH_3/NO of 0.5, this indicates that the overabundance of

adsorbed NH_3, along with unreacted free gas phase NH_3, could have a negative impact on SCR. This hypothesis was confirmed with DFT calculations presented in the manuscript.

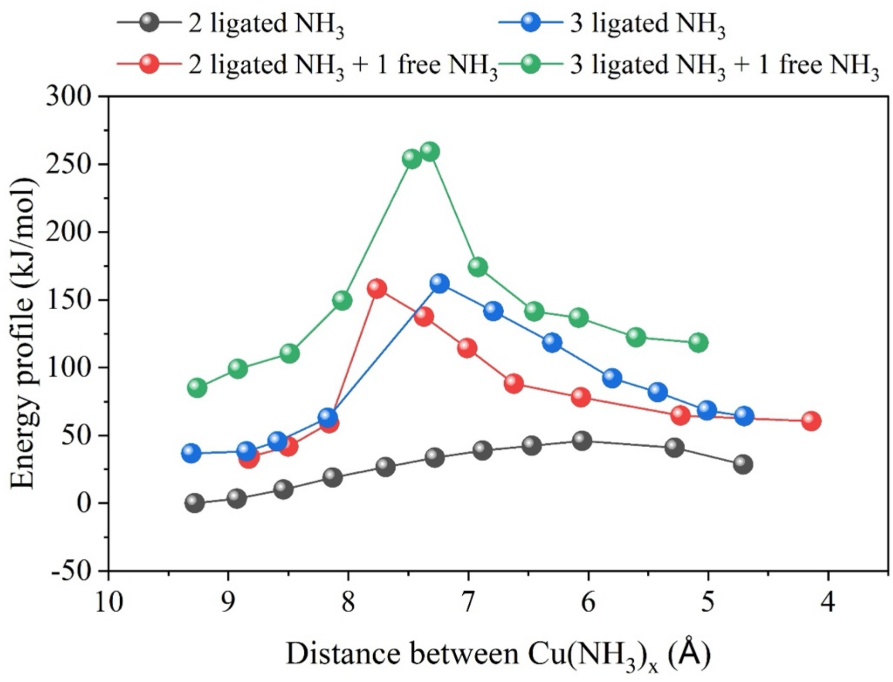

Supplementary Figure 11 | DFT energy profile for $Cu(NH_3)_x$ diffusion. Density functional theory (DFT) calculated energy profiles describing the diffusion of $Cu(NH_3)_x$ ($x = 2$ or 3) species as a function of the distance between Cu centers. All energies are referenced to the configuration with two ligated NH_3 molecules on Cu. Increased NH_3 coordination or the presence of free NH_3 increases barrier to $Cu(NH_3)_x$ diffusion.

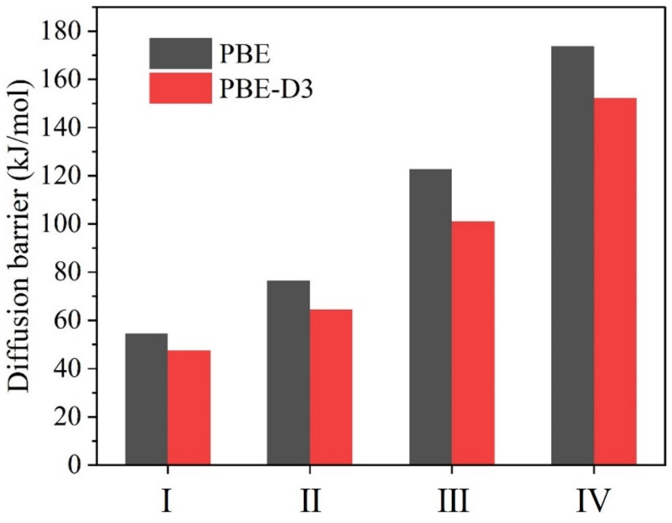

Supplementary Figure 12 | Comparison of $Cu^{I}(NH_3)_x$ (x = 2 or 3) diffusion barrier using PBE and PBE-D3 functionals. To examine the influence of the D3 dispersion correction on our conclusions, we compared the diffusion energy barriers of copper ions calculated using the PBE and PBE-D3 functionals [14]. The result shows, after accounting for long-range interactions, the absolute values of the diffusion barriers for Cu^{I} ions in all four cases decrease by approximately 5–20 kJ/mol. However, this does not alter our qualitative conclusion that excess NH_3 inhibits Cu^{I} ion diffusion. Therefore, the PBE functional remains appropriate for the present study.

Note 6: NH_3-temperature programmed desorption from reduced Cu-0.5 and H-CHA

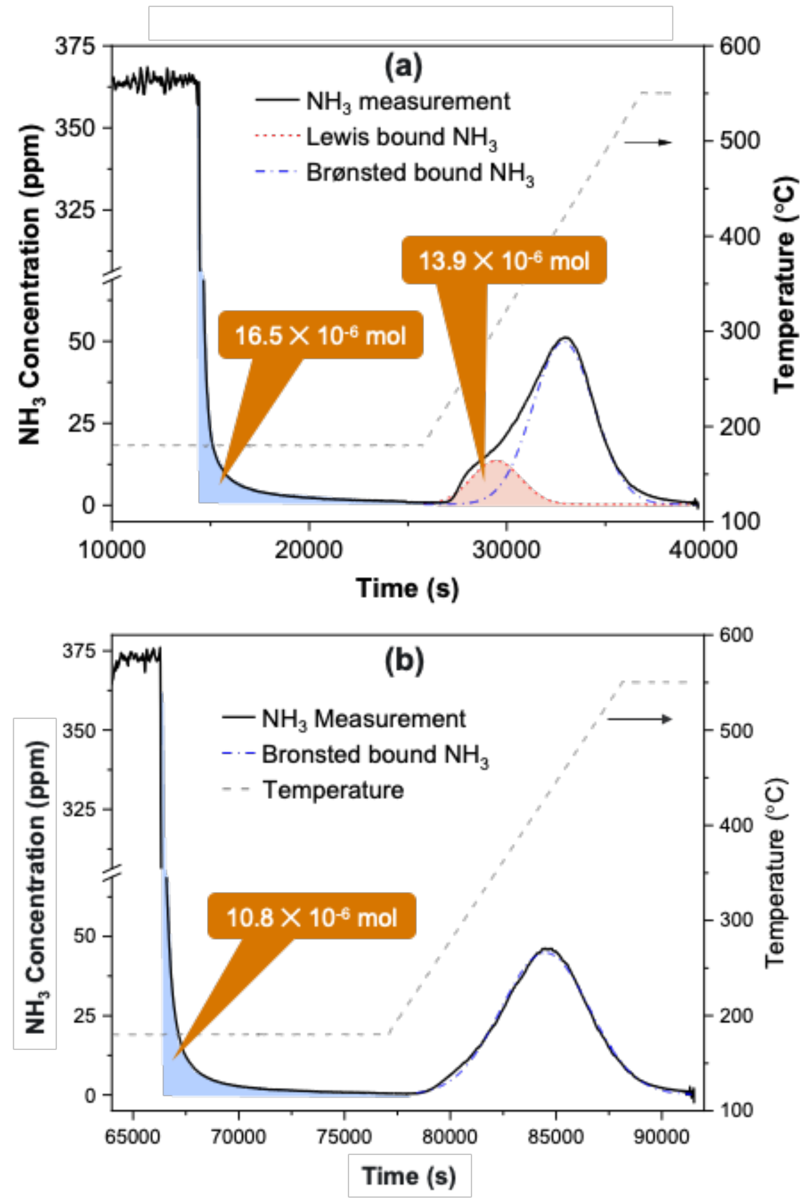

Supplementary Figure 13 | NH_3-TPD comparison of reduced Cu-CHA vs H-CHA. NH_3-temperature programmed desorption (TPD) profiles from (a) Cu-0.5 and (b)H-CHA. The H-CHA in this case is the same zeolite support (Si/Al ~ 12) used to synthesize Cu-0.5. Both plots show NH_3 concentration along primary Y-axis and temperature along secondary Y-axis. The blue shaded region represents weakly-bound NH_3 amount, while the red shaded region represents NH_3 bound strongly on Cu sites. Note that the NH_3 profile collected during temperature ramp phase consists of two peaks for Cu-0.5, and only one peak for

H-CHA. The low temperature peak on Cu-0.5 belongs to NH_3 strongly bound on Cu (area under this peak is the red colored shaded region), while the high temperature peak is attributed to Bronsted-bound NH_3. [10] H-CHA contains only the high-temperature peak due to lack of Cu sites. Note that adsorption of NH_3 was performed in the presence of NO (350 ppm NH_3, 350 ppm NO, balance N_2, 150000/h SV) so that we can calculate the amount of NH_3 adsorbed on a reduced Cu-0.5 sample (i.e., on Cu^I sites). Flow of NH_3 and NO was stopped simultaneously at the point where NH_3 concentration quickly decreases from ~360-370 ppm in **Fig. 13**. A three-hour isothermal hold was used to get rid of weakly-bound NH_3, before ramping up the temperature to perform TPD. Same procedure was also followed for H-CHA.

The area of blue and red shaded regions for Cu-0.5 suggest that the catalyst contains ~16.5 µmol weakly-bound NH_3 and ~13.9 µmol strongly-bound NH_3 (on Cu^I sites). The weakly-bound NH_3 amount on H-CHA was found to be around 10.8 µmol, indicating that Cu-0.5 has around 5.7 µmol of NH_3 weakly bound on Cu^I sites, along with the 13.9 µmol strongly bound NH_3. Since the catalyst amount loaded into the reactor contained around 7.5 µmol of Cu sites, the aforementioned calculation suggests that Cu-0.5 has around 0.75 weakly-bound NH_3 molecules per Cu^I site, in addition to ~2 strongly-bound NH_3 ligands, yielding a total NH_3/Cu^I ratio of 2.75/1. Hence, under the reducing environment of NH_3 and NO, around 75% of the Cu^I sites are present as $[Cu^I(NH_3)_3]^+$ and the other 25% are present as $[Cu^I(NH_3)_2]^+$. Note that the third ligand on $[Cu^I(NH_3)_3]^+$ is only present under NO+NH_3 flow since these NH_3 molecules desorbs quickly during isothermal hold under N_2 flow. These results provide an experimental validation of our DFT calculations that predicts the formation of Cu^I ions containing three NH_3 ligands under excess NH_3 condition.

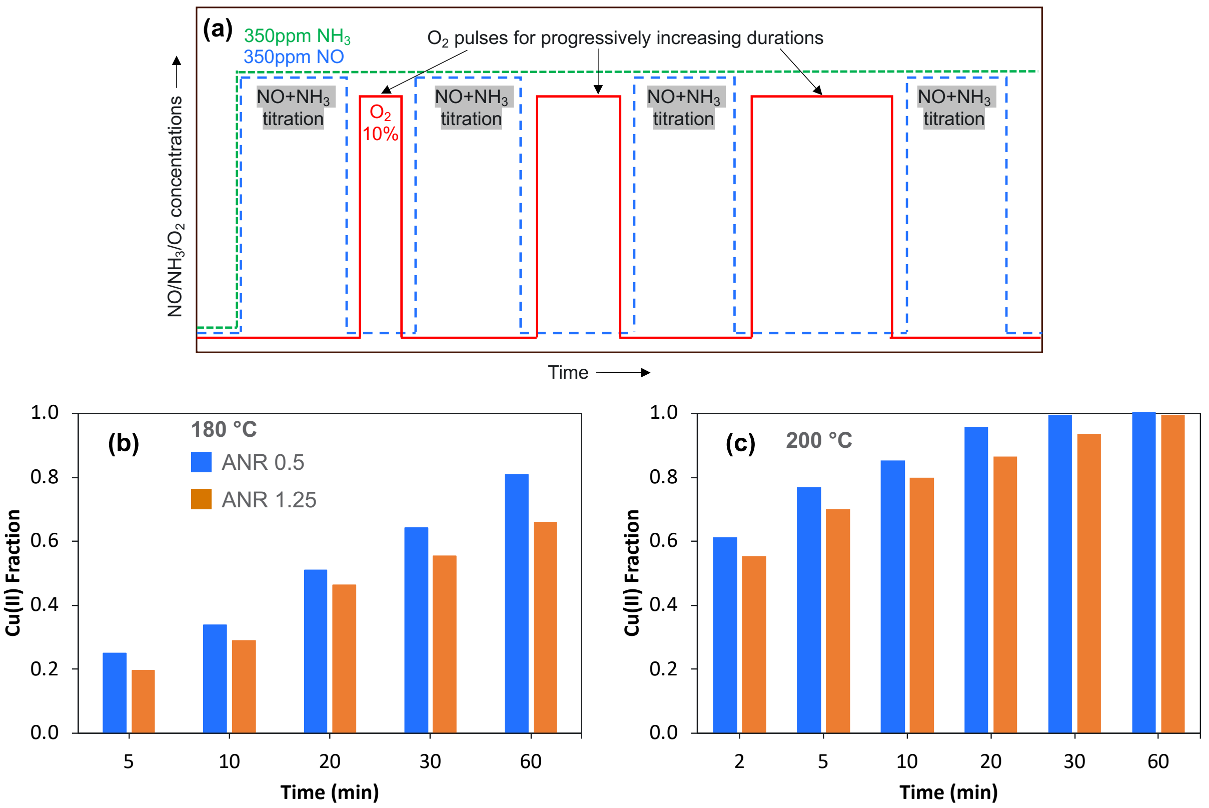

Supplementary Figure 14 | O_2 pulse oxidation kinetics and NH_3 inhibition of Cu^I oxidation. (a) O_2-pulse experimental protocol to probe Cu^I oxidation rate: Initially, 350 ppm NO + 350 ppm NH_3 is flowed over the catalyst (Cu-0.5) to reduce all Cu sites to Cu^I. NO is then turned off and O_2 is introduced for a designated length of time, following which O_2 is turned off. NO and NH_3 is restarted to reduce the Cu^{II} sites formed by O_2-derived oxidation of Cu^I sites, and by calculating the NO consumed at the step provides the number of Cu^I sites oxidized by O_2 pulse. This process is repeated with various time durations of O_2 pulses. The Cu^{II} fraction vs. oxidation time calculated this way for two ANRs: 0.5 and 1.25, at 180 °C and 200 °C, are plotted in **Fig. 14b** and **Fig. 14c**, respectively. the amount of Cu^I oxidized to Cu^{II} during each pulse decreases systematically with increasing ANR from 0.5 to 1.25. This trend directly demonstrates that NH_3-rich conditions suppress the overall oxidation half-cycle.

Note 7: The 3330 G EPR feature is an instrumental artifact

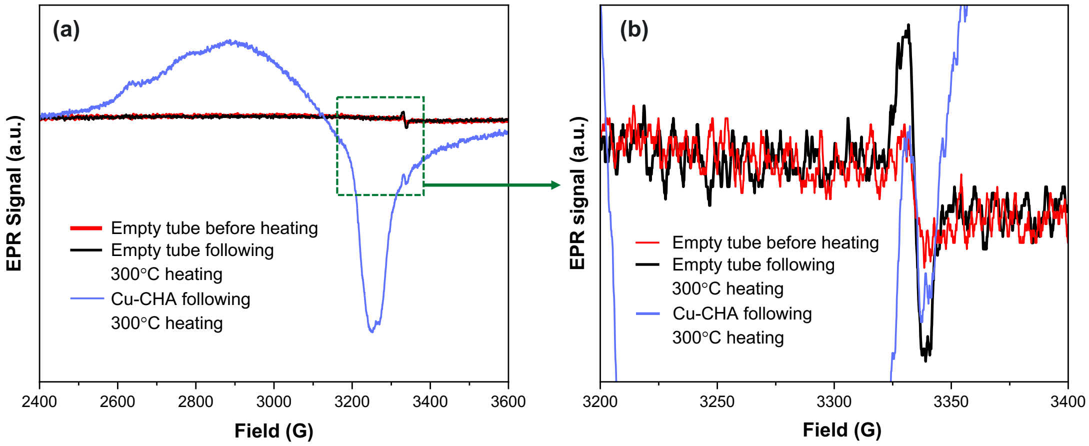

Supplementary Figure 15 | EPR spectra comparison: empty tube vs Cu-0.5 (thermal treatment effects). Continuous wave EPR spectra (a) 2400-3600 G region, (b) 3200-3400 G region. The figures present spectra of three samples- (1) empty sample tube at room temperature, (2) empty tube at room temperature, but after heating it to 300°C and cooling down to room temperature, (3) Cu-0.5 sample at room temperature, but after heating it to 300°C and cooling down to room temperature.

The 3330 G feature is not relevant to our samples. It appears in all spectra, irrespective of temperature or reaction conditions, and its absolute intensity remains constant in all spectra. As a result, it's intensity relative to the Cu signal increases when Cu(II) fraction decreases, for example at high ANRs in **Fig. 9** in the manuscript. These observations tell us that the 3330 G peak is an interference from the instrumental setup.

To confirm this, we collected EPR spectra of the empty sample tube at room temperature before and after heating it to 300°C. We also collected spectra of Cu-0.5 sample at room temperature, following heat treatment at 300°C. **Figure 15a** above shows these three spectra. As expected, the empty tube spectra is a flat line when compared to the Cu-0.5 spectra. All three spectra, however, contain a feature at around 3330 G, the same one that the reviewers' comment addresses. **Figure 15b** provides an enlarged scale

focusing on the 3330 G peak for ease of observation. These results thus confirms that the 3330 G peak comes from the instrument itself and hence is present in all in-situ and ex-situ data presented in this paper.

Note 8: EPR spectra fitting and quantification

The continuous-wave EPR spectra (first-derivative mode) were fitted using a custom Python routine in which each spectrum was represented as the sum of one to three axial Cu(II) components ($I = 3/2$). Each component was parameterized by an axial g-tensor ($g_{\perp}, g_{\parallel}$), an axial hyperfine tensor ($A_{\perp}, A_{\parallel}$, reported in MHz), and a Lorentzian linewidth (γ, in Gauss). Powder-averaged lineshapes were generated by numerical integration over molecular orientations and summation over the four Cu hyperfine lines ($m_I = \pm 3/2, \pm 1/2$). To account for inhomogeneous broadening, g-strain and A-strain terms were included for each component.

The overall spectrum was modeled as a linear combination of site contributions with positive fractions constrained to sum to unity. During fitting, a small field-axis alignment (global shift and scale) and a small microwave-frequency adjustment were allowed. A low-order polynomial baseline (up to quadratic) was included and optimized simultaneously with the spectral parameters. Best-fit parameters were obtained by nonlinear least-squares minimization with bounded constraints.

The operando EPR spectra at each ANR and temperature were fitted using two Cu(II) components:

(i) Site 1, with $g_{\parallel} \approx 2.34$ and $A_{\parallel} \approx 135$ G, assigned to Cu(II) species with dominantly Cu-O_L coordination (Z_2Cu-type sites); and

(ii) Site 2, with $g_{\parallel} \approx 2.27$ and $A_{\parallel} \approx 155$ G, assigned to Cu(II) species with mixed Cu-N/Cu-O_L coordination.

The relative contribution of each site was obtained from the integrated intensity of the fitted components.

At 220 °C, the fitted site distributions show a strong ANR dependence: at ANR = 0.125, Site 1 accounts for ~76 % of the EPR-active Cu(II), with the remaining ~24 % as Site 2. At ANR = 0.25, the Site 1 fraction drops sharply to ~10 %, and for ANR ≥ 0.5 the spectra are fully dominated by Site 2. At 180 °C, Site 1 contributes ~44 % at ANR = 0.125, while Site 2 contributes ~56 %; for ANR ≥ 0.25, Site 2 accounts for essentially 100 % of the EPR-active Cu(II). Representative fits at 220 °C and 180 °C are provided in **Figures 16a-h** and **17a-h**, respectively, and the resulting site distributions are summarized in **Figure 18**.

This quantitative trend demonstrates that increasing NH_3 availability systematically shifts the Cu(II) population from Cu-O_L-dominated sites toward NH_3-coordinated Cu(II) species, with the transition occurring at lower ANR at lower temperature. While it is difficult to obtain absolute Cu speciation from CW-EPR due to overlapping signals and EPR-silent Cu species, this two-site fitting approach yields a robust semi-quantitative measure of the relative Cu-O_L versus Cu-N/Cu-O_L populations and directly links changes in Cu coordination to the observed NH_3-dependent SCR kinetics.

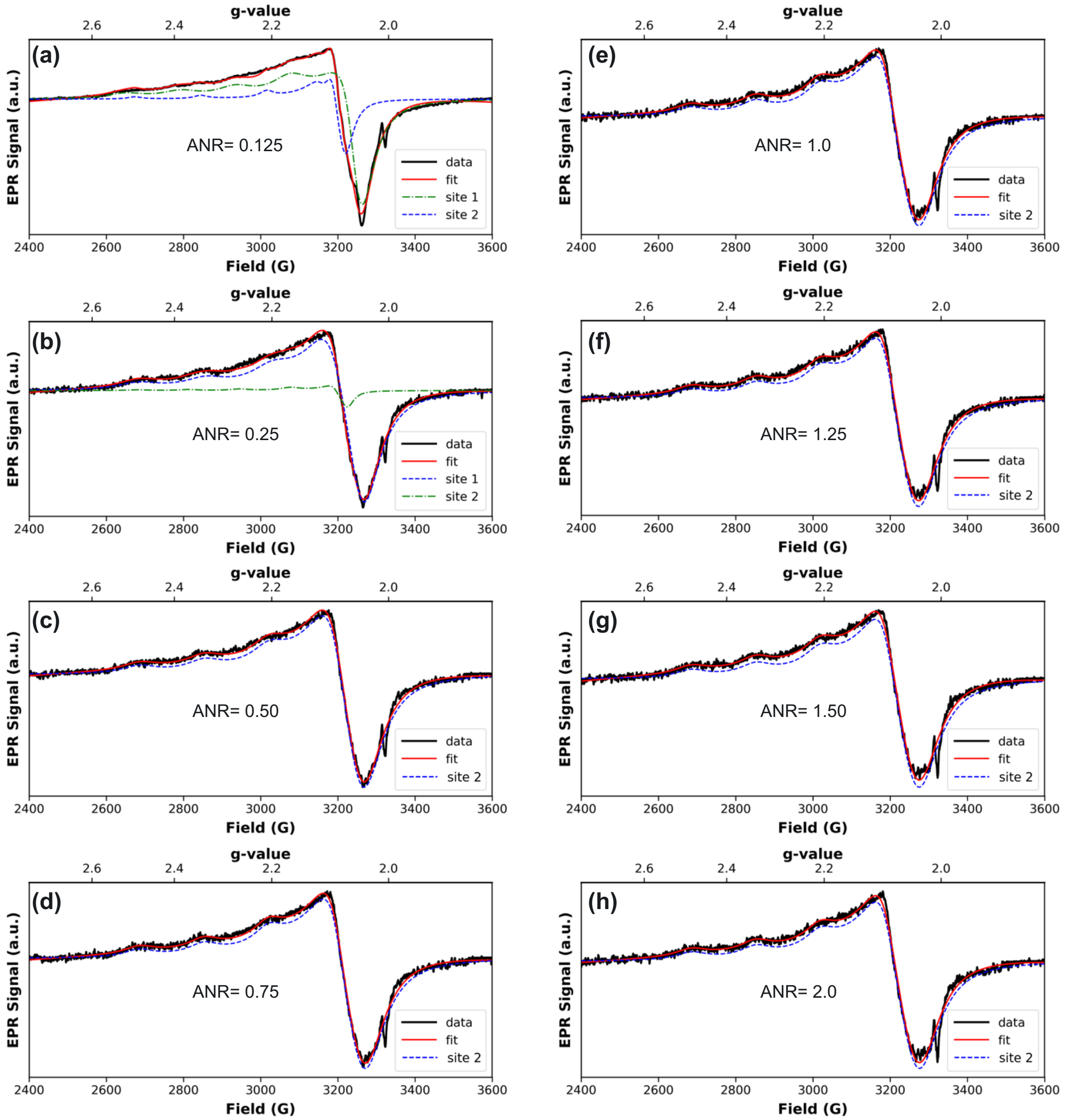

Supplementary Figure 16 | Two Cu-site fitting of operando EPR collected under SCR at 220°C at varying ANRs. Two-site fitting of operando EPR spectra under SCR conditions. Operando CW-EPR spectra of Cu-0.5 acquired at **220 °C** under steady-state standard SCR conditions (500 ppm NO, varying NH_3, 10 % O_2, 3 % H_2O, SV = 400 k h^{-1}) and different ANRs- (a) ANR 0.125, (b) ANR 0.25, (c) ANR 0.50, (d) ANR 0.75, (e) ANR 1.0, (f) ANR 1.25, (g) ANR 1.50, (h) ANR 2.0. Black traces show experimental data, while colored curves represent the best fits obtained using a two-site model consisting of Site 1 ($g_\parallel \approx 2.33$, $A_\parallel \approx 130$ G; Cu–O_L-dominated coordination) and Site 2 ($g_\parallel \approx 2.27$, $A_\parallel \approx 155$ G;

mixed Cu–N/Cu–O coordination). The relative contributions of the two sites were determined from the integrated intensity of the fitted components and are summarized in **Figure 18**.

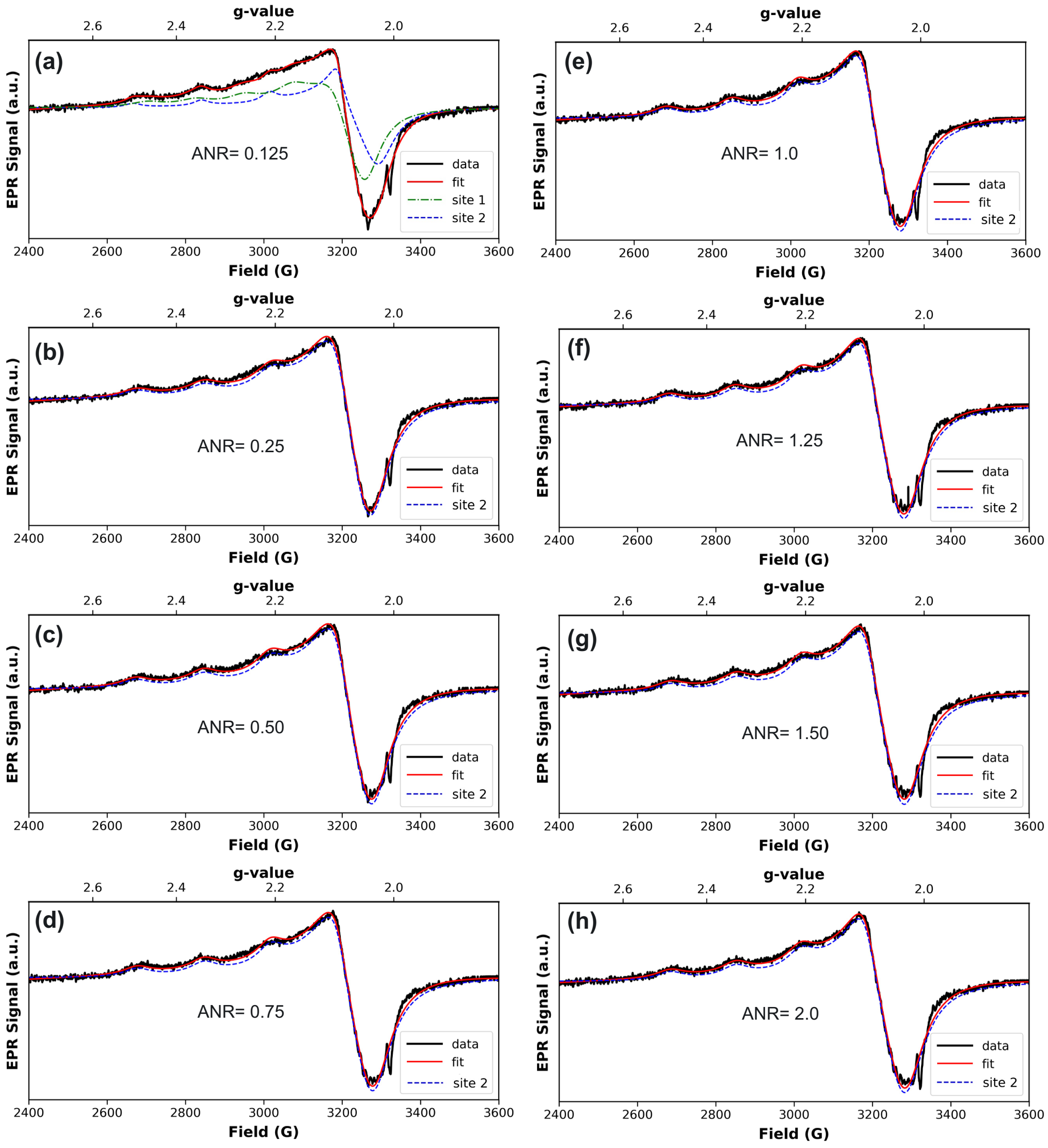

Supplementary Figure 17 | Two Cu-site fitting of operando EPR collected under SCR at 220°C at varying ANRs. Two-site fitting of operando EPR spectra under SCR conditions. Operando CW-EPR spectra of Cu-0.5 acquired at **180 °C** under steady-state standard SCR conditions (500 ppm NO, varying NH_3, 10 % O_2, 3 % H_2O, SV = 400 k h^{-1}) and different ANRs- (a) ANR 0.125, (b) ANR 0.25, (c) ANR 0.50, (d) ANR 0.75, (e) ANR 1.0, (f) ANR 1.25, (g) ANR 1.50, (h) ANR 2.0. Black traces show

experimental data, while colored curves represent the best fits obtained using a two-site model consisting of Site 1 (g∥ ≈ 2.33, A∥ ≈ 130 G; Cu–O_L-dominated coordination) and Site 2 (g∥ ≈ 2.27, A∥ ≈ 155 G; mixed Cu–N/Cu–O coordination). The relative contributions of the two sites were determined from the integrated intensity of the fitted components and are summarized in **Figure 18**.

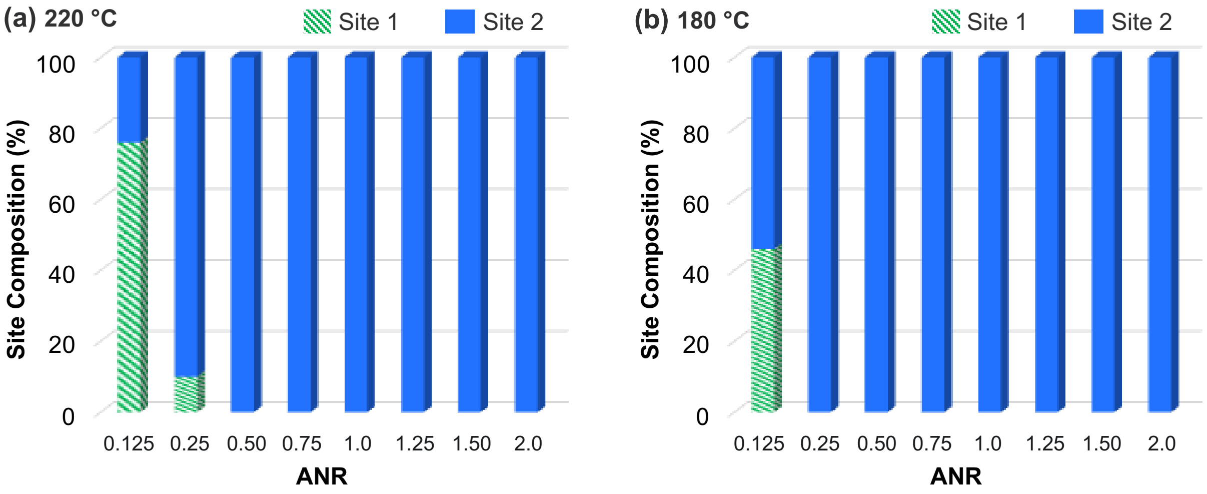

Supplementary Figure 18 | ANR-dependent distribution of Cu^{II} species from operando EPR. Distribution of EPR-active Cu^{II} species as a function of ANR. Relative fractions of Site 1 (Cu–O_L-dominated coordination; g∥ ≈ 2.33, A∥ ≈ 130 G) and Site 2 (mixed Cu–N/Cu–O coordination; g∥ ≈ 2.27, A∥ ≈ 155 G), obtained from two-site fitting of operando EPR spectra of Cu-0.5 under steady-state SCR conditions at (a) 220 °C and (b) 180 °C. The site fractions were calculated from the integrated intensity of the fitted components shown in Figures 7 and 8. Increasing ANR leads to a progressive shift from Cu–O_L-dominated sites to NH_3-coordinated Cu^{II} species, with the transition occurring at lower ANR at lower temperature.

Note 9: Ex-situ EPR spectra of Cu-0.5

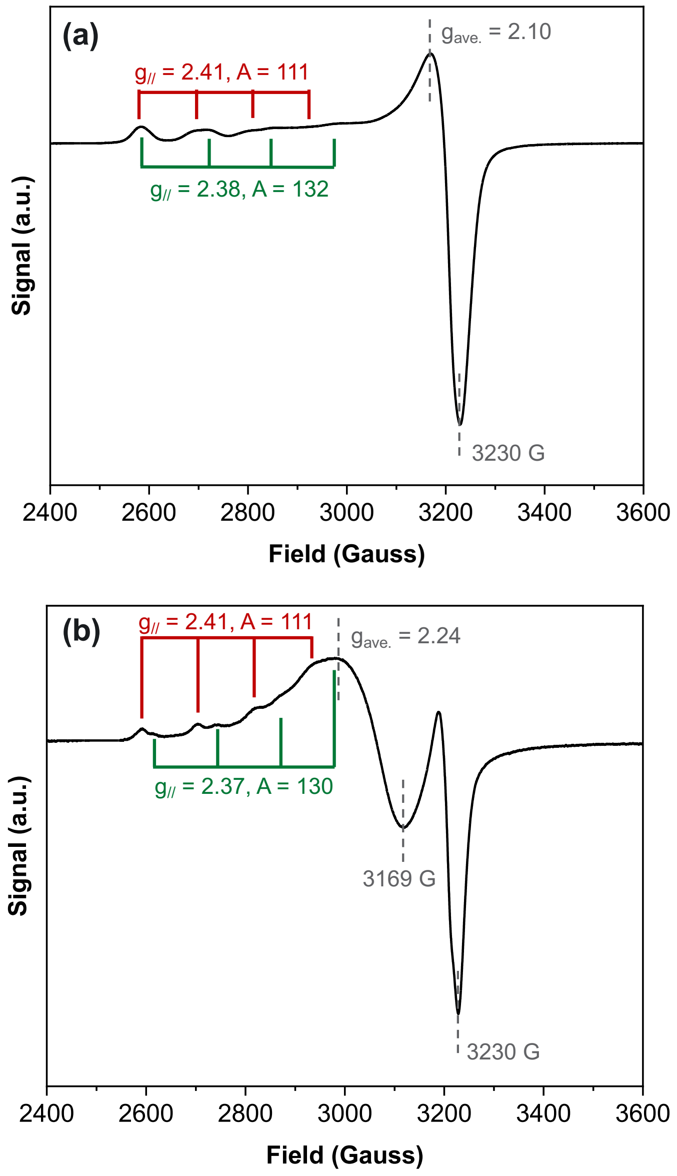

Supplementary Figure 19 | Ex-situ EPR spectra of Cu-0.5. EPR spectra of ydrated Cu-0.5 at (a) −150 °C and (b) 25 °C. The marked hyperfine parameters indicate that Cu^{II} ions are present in anisotropic geometries.

The hyperfine features at $g_{//}$ = 2.41, $A_{//}$ = 111 G and $g_{//}$ = 2.38 G, $A_{//}$ = 132 G at −150 °C are attributed to hydrated Cu^{II} ions with anisotropic geometries. [15] High field feature at 3230 G is also attributed to anisotropic Cu^{II} ions. [15] Spectra at 25 °C also contains similar hyperfine features as those at −150 °C. Since Cu-0.5 is a low-Cu sample, the Cu^{II} sites experience relatively higher electrostatic attraction to the zeolite framework, which probably leads to similar anisotropic features as −150 °C at 25 °C. The high field region at 25 °C has two overlapping signals: one at 3230 G which is attributed to anisotropic Cu^{II}, and the other at 3169 G attributed to isotropic Cu^{II} ions. Presence of such isotropic Cu^{II} also results in a lumped hyperfine feature at g_{ave} = 2.24.[16,17]

Note 10: Double integration of EPR signal

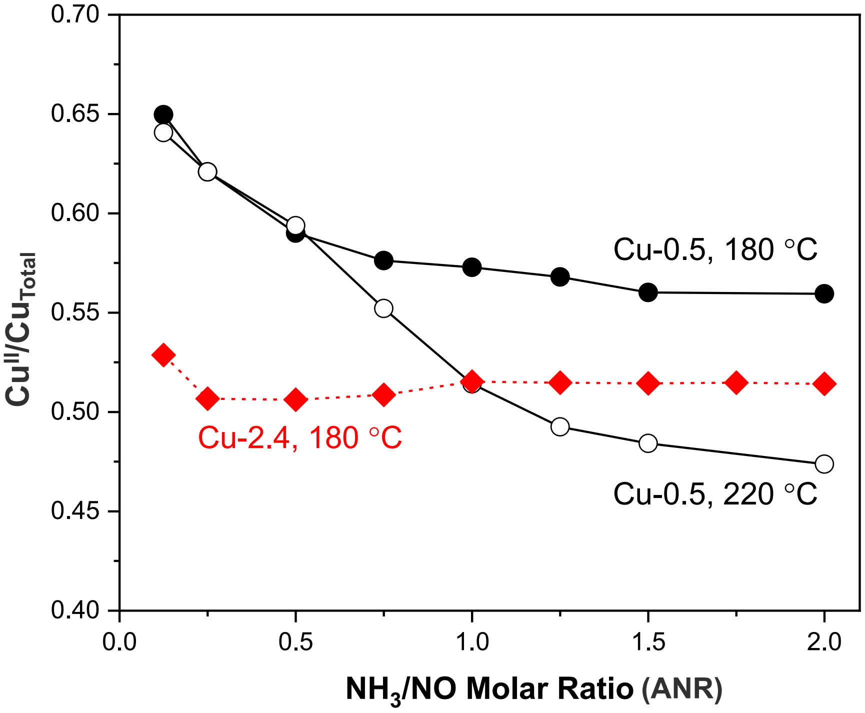

Supplementary Figure 20 | Cu^{II} fraction during SCR at varying ANRs. Cu^{II} fraction at steady state SCR Cu-0.5 (180 °C and 220 °C) and Cu-2.5 (180 °C) as a function of NH_3/NO molar ratio.

The Cu^{II} fraction was calculated with the following method: For Cu-0.5 at 100 °C, almost all Cu ions are in Cu^{II} state since SCR does not light off on this catalyst at this temperature. Hence, the double integrated signal of operando spectra at 100 °C was taken as the signal for total Cu on the catalyst. Signals of all other spectra were normalized with respect to that of 100 °C to find the respective Cu^{II} fractions. Since EPR signal is inversely proportional to temperature, a temperature correction was also used for these calculations. The same procedure was also used to calculate Cu^{II} fractions at steady state SCR on Cu-2.5, except for the reference spectra was collected at 50 °C instead of 100 °C, since a small NO_x reduction is observed on Cu-2.5 at 100 °C.

Note 11: Operando EPR spectra of Cu-2.5

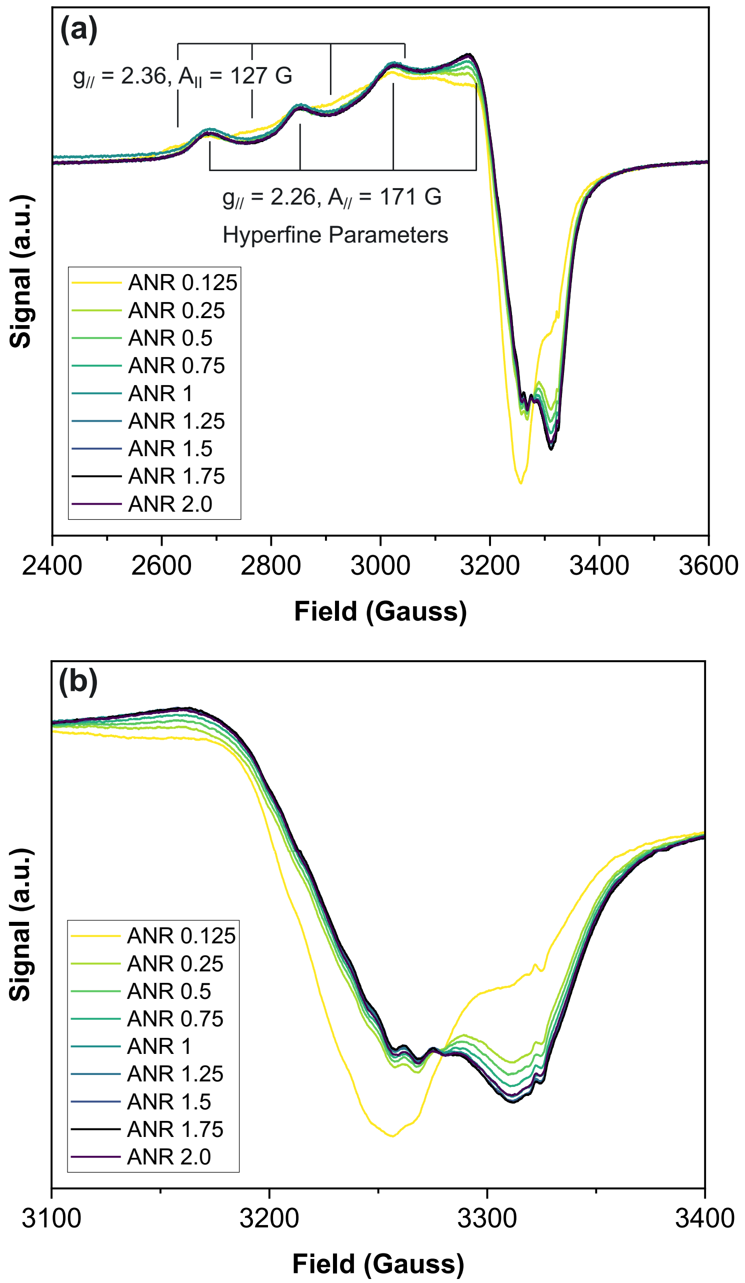

Supplementary Figure 21 | Operando EPR spectra of Cu-2.5 under SCR vs ANR (180 °C). *Operando* EPR spectra acquired under steady-state standard SCR on Cu-2.5 sample at various NH_3/NO molar ratios at 180 °C. Feed conditions: 500 ppm NO, 500 ppm NH_3, 10% O_2, 3% H_2O, SV 400k/h. (a) the complete spectra between 2400 G to 3600 G, (b) only high field spectra between 3100 G to 3400 G.

All spectra except for ANR 0.125 primarily consists of hyperfine signals with parameters $g_{//}$ = 2.26 and $A_{//}$ = 171 G. These tensor values are attributed to $Cu^{II}(NH_3)_n$ (n≤5) complexes. [18, 19] The spectra for ANR 0.125 contains not only the aforementioned hyperfine feature, but also another one with parameters $g_{//}$ = 2.36 and $A_{//}$ = 127 G, which is attributed to immobilized Cu-O_L species. [17] This suggests that the low ANR condition leads to a fraction of the total Cu^{II} ions to be NH_3-solvated while the others remain framework-bound. This is further corroborated by the high field region where the spectra for ANR 0.125 exhibits just one primary signal at 3240 G, attributed to Cu^{II} ions with mixed Cu-O_L/Cu-N coordination. [19] In contract, the high field regions in all other ANR cases consist of two overlapping signals at 3240 G and 3300 G. The one at 3240 G has already been attributed to Cu-O_L/Cu-N. The signal at 3300 G can be attributed to $Cu^{II}(NH_3)_4$ or $Cu^{II}(OH)(NH_3)_3$, [19] which aligns with our understanding that the Cu^{II} sites are well-solvated by NH_3 at these higher ANRs.

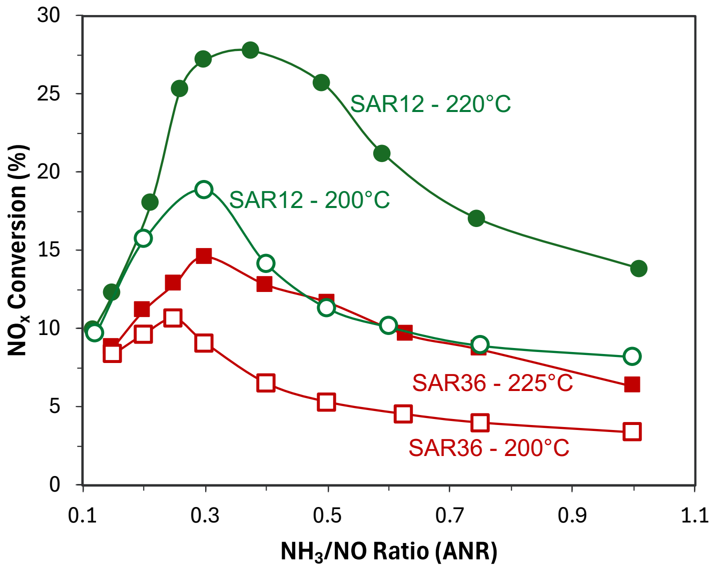

Figure 22 | Effect of Si/Al ratio (SAR) on NH_3 inhibition. Figure shows SCR NO_x conversion on two samples with 0.5 wt% Cu but varying SARs of 12 and 36, at different temperatures and different ANRs. The sample with SAR36 shows lower NO_x conversion and lower critical ANR for NH_3 inhibition onset, hence a greater degree of NH_3 inhibition at any given temperature.

Supplementary References:

(1) Krishna, S. H.; Jones, C. B.; Gounder, R. Temperature dependence of Cu (I) oxidation and Cu (II) reduction kinetics in the selective catalytic reduction of NOx with NH3 on Cu-chabazite zeolites. *Journal of Catalysis* **2021**, *404*, 873-882.

(2) Jones, C. B.; Khurana, I.; Krishna, S. H.; Shih, A. J.; Delgass, W. N.; Miller, J. T.; Ribeiro, F. H.; Schneider, W. F.; Gounder, R. Effects of dioxygen pressure on rates of NOx selective catalytic reduction with NH3 on Cu-CHA zeolites. *Journal of catalysis* **2020**, *389*, 140-149.

(3) Deka, D. J.; Daya, R.; Ladshaw, A.; Trandal, D.; Joshi, S. Y.; Partridge, W. P. Assessing impact of real-world aging on Cu-redox half cycles of a Cu-SSZ-13 SCR catalyst via transient response measurements and kinetic modeling. *Applied Catalysis B: Environmental* **2022**, *309*, 121233.

(4) Nasello, N. D.; Iacobone, U.; Usberti, N.; Gjetja, A.; Nova, I.; Tronconi, E.; Villamaina, R.; Ruggeri, M. P.; Bounechada, D.; York, A. P. Investigation of low-temperature OHC and RHC in NH3–SCR over Cu-CHA catalysts: effects of H2O and SAR. *ACS Catalysis* **2024**, *14* (6), 4265-4276.

(5) Kresse, G.; Furthmüller, J. Efficiency of ab-initio total energy calculations for metals and semiconductors using a plane-wave basis set. *Computational materials science* **1996**, *6* (1), 15-50.

(6) Kresse, G.; Hafner, J. Ab initio molecular dynamics for liquid metals. *Physical review B* **1993**, *47* (1), 558.

(7) Perdew, J. P.; Burke, K.; Ernzerhof, M. Generalized gradient approximation made simple. *Physical review letters* **1996**, *77* (18), 3865.

(8) Monkhorst, H. J.; Pack, J. D. Special points for Brillouin-zone integrations. *Physical review B* **1976**, *13* (12), 5188.

(9) Wang, V.; Xu, N.; Liu, J.-C.; Tang, G.; Geng, W.-T. VASPKIT: A user-friendly interface facilitating high-throughput computing and analysis using VASP code. *Computer Physics Communications* **2021**, *267*, 108033.

(10) Daya, R.; Deka, D. J.; Goswami, A.; Menon, U.; Trandal, D.; Partridge, W. P.; Joshi, S. Y. A redox model for NO oxidation, NH3 oxidation and high temperature standard SCR over Cu-SSZ-13. *Applied Catalysis B: Environmental* **2023**, *328*, 122524.

(11) Luo, J.; Gao, F.; Kamasamudram, K.; Currier, N.; Peden, C. H.; Yezerets, A. New insights into Cu/SSZ-13 SCR catalyst acidity. Part I: Nature of acidic sites probed by NH3 titration. *Journal of catalysis* **2017**, *348*, 291-299.

(12) Gao, F.; Mei, D.; Wang, Y.; Szanyi, J.; Peden, C. H. Selective Catalytic Reduction over Cu/SSZ-13: Linking Homo- and Heterogeneous Catalysis. *J Am Chem Soc* **2017**, *139* (13), 4935-4942.

(13) Paolucci, C.; Khurana, I.; Parekh, A. A.; Li, S.; Shih, A. J.; Li, H.; Di Iorio, J. R.; Albarracin-Caballero, J. D.; Yezerets, A.; Miller, J. T. Dynamic multinuclear sites formed by mobilized copper ions in NO x selective catalytic reduction. *Science* **2017**, *357* (6354), 898-903.

(14) Grimme, S.; Antony, J.; Ehrlich, S.; Krieg, H. A consistent and accurate ab initio parametrization of density functional dispersion correction (DFT-D) for the 94 elements H-Pu. *The Journal of chemical physics* **2010**, *132* (15).

(15) Wu, Y.; Zhao, W.; Ahn, S. H.; Wang, Y.; Walter, E. D.; Chen, Y.; Derewinski, M. A.; Washton, N. M.; Rappe, K. G.; Wang, Y.; et al. Interplay between copper redox and transfer and support acidity and topology in low temperature NH(3)-SCR. *Nat Commun* **2023**, *14* (1), 2633.

(16) Zhang, Y.; Wu, Y.; Peng, Y.; Li, J.; Walter, E. D.; Chen, Y.; Washton, N. M.; Szanyi, J.; Wang, Y.; Gao, F. Quantitative Cu Counting Methodologies for Cu/SSZ-13 Selective Catalytic Reduction Catalysts by Electron Paramagnetic Resonance Spectroscopy. *The Journal of Physical Chemistry C* **2020**, *124* (51), 28061-28073.

(17) Godiksen, A.; Vennestrøm, P. N.; Rasmussen, S. B.; Mossin, S. Identification and quantification of copper sites in zeolites by electron paramagnetic resonance spectroscopy. *Topics in Catalysis* **2017**, *60*, 13-29.

(18) Moreno-González, M.; Hueso, B.; Boronat, M.; Blasco, T.; Corma, A. Ammonia-containing species formed in Cu-chabazite as per in situ EPR, solid-state NMR, and DFT calculations. *The journal of physical chemistry letters* **2015**, *6* (6), 1011-1017.

(19) Wu, Y.; Ma, Y.; Wang, Y.; Rappe, K. G.; Washton, N. M.; Wang, Y.; Walter, E. D.; Gao, F. Rate Controlling in Low-Temperature Standard NH(3)-SCR: Implications from Operando EPR Spectroscopy and Reaction Kinetics. *J Am Chem Soc* **2022**, *144* (22), 9734-9746.
