# Supplementary material for: Insights into the mechanisms of NH3 inhibition on Cu-CHA SCR catalysts
Source: Nat Commun. 2026 Jul 28;17:7421. doi: 10.1038/s41467-026-72879-7 (PMC13408143; doi:10.1038/s41467-026-72879-7)
Supplement: Supplementary file 2 — Transparent Peer Review file [file 41467_2026_72879_MOESM2_ESM.pdf]

nature portfolio

Peer Review File

Insights into the mechanisms of NH3 inhibition on Cu-CHA SCR catalysts

Corresponding Author: Professor Yong Wang

This file contains all reviewer reports in order by version, followed by all author rebuttals in order by version.

Version 0:

Reviewer comments:

Reviewer #1

(Remarks to the Author)
This work thoroughly analyzes the effect of the NH3 concentration on the low-temperature NH3-SCR redox mechanism over Cu-chabazite catalysts. To my understanding, this represents a novelty in scientific literature and a further step towards the full elucidation of the NH3-SCR redox mechanism over Cu-exchanged zeolites. I really appreciate the work performed by the Authors: the coupling of steady-state and, more importantly, transient tests, DFT analysis and spectroscopic measurements is a solid approach to assess the mechanistic features of catalytic processes. More in detail, the inhibiting effect of high NH3/NO feed ratios on the oxidation half-cycle of the NH3-SCR redox mechanism is clearly highlighted and explained.
I have a few comments for the authors, that hopefully will help to further improve the quality of the manuscript.

1) The authors tested catalysts with different Cu loadings and aging treatments. It is not always immediate to understand on which catalysts they collected the data presented in the Figures and Tables. Please, update the captions to clarify this aspect.

2) In the “SCR kinetics and NH3 inhibition” section of the main manuscript, the Authors analyzed their low-temperature NH3-SCR data according to two very different redox kinetic approaches: i) reduction and oxidation rate constants are extracted from steady-state data at different O2 concentrations (Supplementary, Fig.4) assuming first-order RHC and OHC kinetics, see eq. (1): the “Langmuirian” rate expression is readily derived combining first order redox kinetics for RHC and OHC in oxidized and reduced Cu sites, respectively; ii) transient data (Supplementary, Fig.5) are instead analyzed with a different rate model based on second order kinetics for RHC and OHC. I do not fully understand the reason for applying such two distinct models. Furthermore, considering that at least OHC is well known to be second order in the reduced Cu sites (as also described by the Authors in the manuscript), the kinetic constants estimated by the Langmuir-type approach are formally flawed.

3) Analysis of RHC-STDSCR-RHC tests: to estimate the amount of oxidized Cu sites at steady-state NH3-SCR conditions, in my view the Authors should compute the integral amount of NO converted during the final reduction stage of the test, not during the oxidation transient to STDSCR steady state. During this transient, in fact, Cu sites are being oxidized and reduced at the same time, thus it is impossible to decouple the two contributions.

4) The data for the oxidized Cu sites reported in the description of Figure 7 are probably inverted: “At 200 °C, amount of CuI that oxidized to CuII was calculated to be 0.98 µmols and 2.1 µmols at ANR = 0.5 and 1.25, respectively”. Considering the NH3 inhibition of the OHC, at high ANR a lower CuII amount should be expected.

5) The Authors tested also two aged catalyst samples. Do these data reveal the effect of aging on the NH3 inhibition? Is the NH3 inhibition stronger over aged catalysts? I think that this aspect should be mentioned in the manuscript.

Reviewer #2

(Remarks to the Author)
I co-reviewed this manuscript with one of the reviewers who provided the listed reports. This is part of the Nature

Communications initiative to facilitate training in peer review and to provide appropriate recognition for Early Career Researchers who co-review manuscripts.

Reviewer #3

(Remarks to the Author)
The paper addresses a key aspect of the chemistry of the NH3 inhibition of the Ci chabazite ammonia SCR de-NOx catalyst, namely the ammonia inhibition mechanism which it addresses by a joint computational experimental study.
The work is interesting and relevant; however, some specific details, as given below, are required to further improve the quality of the manuscript.
• The authors state that ammonia-to-NOx ratios (ANR) mainly collapse the pre-exponential while Ea changes little, implying an entropic/mobility penalty. Can the authors report ΔG‡(T) (not only energy barriers) for intercage diffusion of $[Cu^{1+}(NH_3)_2]^+$ / $[Cu^{1+}(NH_3)_3]^+$ with and without a free NH_3 in the cage?

• In the DFT, the authors used PBE, but it would be good to see PBE-D3 cross-check to show that the NH_3-crowding trend in diffusion barriers is robust to dispersion treatment.

• The authors emphasise the oxidation half cycle (OHC) throttling under excess NH_3 and attribute it to hindered Cu^+ mobility; however, the present DFT treats only Cu^+ intercage diffusion barriers as NH_3 ligation/free-NH_3 increase, not O_2 binding, O–O activation, or the binuclear Cu–oxo intermediate itself. Can the authors provide O_2-step experiments and DFT of O_2 activation, which will align their emphasis on OHC with a complete demonstration of how O interacts with Cu to convert Cu(I)→Cu(II) under high-ANR, crowded conditions?

The articles below relate to Cu dimer chemistry:
1. Science 357, 898-903 (2017).
2. Chem. Soc. Rev., 2024, 53, 11657-11691

• The authors discuss that excess reactants (NO, H_2O, O_2) can raise $[Cu^{1+}(NH_3)_2]^+$ diffusion barriers. Authors should include one explicit co-adsorbate case to verify this claim.

Minor comments: The colour code "white" should be for H, not for O in Figure 5. e.g. Colour code: orange (Cu), blue (N), white (O), yellow (Si), pink (Al).

Reviewer #4

(Remarks to the Author)
The manuscript " Insights into the mechanisms of NH3 inhibition on Cu-CHA SCR catalysts" investigates a copper substituted chabazite zeolite with different copper concentrations.
The catalytic system has already been heavily investigated in literature. The focus here is to gain more information about the onset conditions and the degree of inhibition on the SCR reaction by excess ammonia.
The authors take a very systematic approach. They use kinetic modelling to unravel the rate coefficients of both the reduction half cycle and of the oxidation half cycle at different ratios of ammonia to nitrogen oxide and different temperatures. To achieve this, they use both steady state and transient conversion data. The most important finding is the change in the pre-exponential factor of the oxidation half cycle as excess of ammonia is present.
Since the evidence points towards limited diffusion of reduced copper species, a theoretical (DFT) investigation is carried out to determine the diffusion of different Cu(I) ammine species. It is concluded that the mobility of Cu(I) in the cages which is necessary for the low temperature oxidation half cycle is severely lessened as the third ammonia attaches to Cu.
A large part of the manuscript is devoted to an operando EPR investigation to follow the Cu(II) species at steady state SCR conditions at different temperatures and quantify the ratio between Cu(I) and Cu(II).
Separately, each of the investigations provides a few new insights but not significant progress. Taken all together, they provide compelling evidence of exactly where the ammonia inhibition begins to limit the SCR activity. The comprehensive data collection on the same sample series allows better comparisons of the evidence across methods that each have different strengths and weaknesses.
In total, the manuscript gives a solid foundation for describing the ammonia inhibition of the SCR reaction on low concentration Cu Chabazite. The conclusions are also extended towards higher concentrations of copper. The manuscript is publishable with minor revisions as given below. The novelty level is borderline for Nature Communications.

The transient kinetic experiments start with Cu fully saturated with ammonia. This raises two concerns:
1. It has previously been described that copper can be autoreduced in ammonia atmosphere at some of the higher temperatures investigated if left long enough. Was the rate of autoreduction determined and deemed irrelevant for the kinetic experiments?
2. The equilibrium state for a sample fully saturated in ammonia is likely to have all Cu at a specific, large dispersion level in the zeolite cages. Cu will have to move the maximal amount from this state to form a dimer in the oxidation half cycle. If a sample is left at SCR conditions, the mean Cu-Cu distance before oxidation is likely always lower, since some copper will not have had time to disperse fully. This might have an impact on the kinetics not observed in this experiment.

Experimental details for the EPR measurements are missing. Wat are the instrument settings and, importantly, the frequency. The field values are not a useful measure on their own, since they will deviate from instrument to instrument.
The EPR spectra almost all show a clear line at 3330 G, which is marked, but not explained. Is it an impurity in the zeolite support or is it a relevant signal?
The analysis of EPR spectra is of a descriptive nature. It would have been beneficial to attempt a more quantitative approach to the data analysis. Is it possible to give an indication of how much of each type of EPR active species is present?

Reviewer #5

(Remarks to the Author)
I co-reviewed this manuscript with one of the reviewers who provided the listed reports. This is part of the Nature Communications initiative to facilitate training in peer review and to provide appropriate recognition for Early Career Researchers who co-review manuscripts.

Version 1:

Reviewer comments:

Reviewer #1

(Remarks to the Author)
First, I congratulate the authors for their extensive work in addressing the reviewers' comments. I was truly impressed by the amount of new data and analysis added to the manuscript and to the supplementary material, which clarifies many of the questions raised.
However, I still have some clarifications to ask regarding the authors' reply to question number 2 in my previous review.
In their rebuttal, the authors claim that the second order Cu(I) OHC kineitcs established the true stoichiometry of the OHC step, while the first-order Langmuir-type analysis of steady-state SCR rates summarizes how the full catalytic cycle responds to O2 pressure. While it is clear that these two approaches are used to address two different levels of kinetic information, the authors do not clarify this discrepancy in the main manuscript: if not fully explained, analyzing STD-SCR activity data with first order RHC and OHC kinetics and, immediately after, modelling RHC and OHC transients with second order kinetics is confusing and contradictory.
I think that, for the sake of clarity, the authors should clearly address this point in the main text. As they write in the Reviewer Response: “the Langmuir-type expression we applied in the steady-state analysis (Fig. 3 in the manuscript) is not meant to represent the elementary Cu(I) kinetics. Its purpose is to describe how the observed macroscopic SCR rate depends on O2 pressure under steady-state conditions. Krishna et al. has shown that for samples with low Cu-loading, like the ones used in our study, the apparent O2 reaction order is close to unity”.

Furthermore, the authors performed OHC transient tests to isolate the elementary Cu(I) oxidation kinetics based on its 2nd order dependence on Cu. However, the kinetic analysis of these new data (reported in the Reviewer Response) was not added to the manuscript. Since reduction transients and their modeling are already included, I think that this OHC kinetic analysis will represent a meaningful addition to this work, completing the STD-SCR redox cycle investigation.

Reviewer #2

(Remarks to the Author)
I co-reviewed this manuscript with one of the reviewers who provided the listed reports. This is part of the Nature Communications initiative to facilitate training in peer review and to provide appropriate recognition for Early Career Researchers who co-review manuscripts.

Reviewer #3

(Remarks to the Author)
The authors have responded in great detail to my earlier comments. This is good work on an important system and I am now happy to recommend that it is accepted.

Reviewer #4

(Remarks to the Author)
In the revised manuscript, the authors have followed up on the concerns of all reviewers conscientiously. The manuscript is publishable in the current form with a small correction:

Please delete the comment "We have incorporated this analysis and discussion into the revised manuscript" at the bottom of page 26 in the supporting information.

Reviewer #5

(Remarks to the Author)

I co-reviewed this manuscript with one of the reviewers who provided the listed reports. This is part of the Nature Communications initiative to facilitate training in peer review and to provide appropriate recognition for Early Career Researchers who co-review manuscripts.

Version 2:

Reviewer comments:

Reviewer #1

(Remarks to the Author)
The authors have addressed my concerns, the paper can be published in its present form.

Open Access This Peer Review File is licensed under a Creative Commons Attribution 4.0 International License, which permits use, sharing, adaptation, distribution and reproduction in any medium or format, as long as you give appropriate credit to the original author(s) and the source, provide a link to the Creative Commons license, and indicate if changes were made.
In cases where reviewers are anonymous, credit should be given to 'Anonymous Referee' and the source.
The images or other third party material in this Peer Review File are included in the article's Creative Commons license, unless indicated otherwise in a credit line to the material. If material is not included in the article's Creative Commons license and your intended use is not permitted by statutory regulation or exceeds the permitted use, you will need to obtain permission directly from the copyright holder.
To view a copy of this license, visit https://creativecommons.org/licenses/by/4.0/

Responses to Reviewers' Comments

Reviewer #1:

This work thoroughly analyzes the effect of the NH_3 concentration on the low-temperature NH_3-SCR redox mechanism over Cu-chabazite catalysts. To my understanding, **this represents a novelty in scientific literature** and a further step towards the full elucidation of the NH_3-SCR redox mechanism over Cu-exchanged zeolites. I really appreciate the work performed by the Authors: the coupling of steady-state and, more importantly, transient tests, DFT analysis and spectroscopic measurements is a solid approach to assess the mechanistic features of catalytic processes. More in detail, the inhibiting effect of high NH_3/NO feed ratios on the oxidation half-cycle of the NH_3-SCR redox mechanism is clearly highlighted and explained.

I have a few comments for the authors, that hopefully will help to further improve the quality of the manuscript.

1) The authors tested catalysts with different Cu loadings and aging treatments. It is not always immediate to understand on which catalysts they collected the data presented in the Figures and Tables. Please, update the captions to clarify this aspect.

Response:

We are thankful to the reviewer for finding these issues. Upon inspection, we found that Figures 3, 4 and Table 1 lacked information on the samples. We have revised the captions to show that these data were collected on Cu-0.5 sample.

2) In the "SCR kinetics and NH_3 inhibition" section of the main manuscript, the Authors analyzed their low-temperature NH_3-SCR data according to two very different redox kinetic approaches: i) reduction and oxidation rate constants are extracted from steady-state data at different O_2 concentrations (Supplementary, Fig.4) assuming first-order RHC and OHC kinetics, see eq. (1): the "Langmuirian" rate expression is readily derived combining first order redox kinetics for RHC and OHC in oxidized and reduced Cu sites, respectively; ii) transient data (Supplementary, Fig.5) are instead analyzed with a different rate model based on second order kinetics for RHC and OHC. I do not fully understand the reason for applying such two distinct models. Furthermore, considering that at least OHC is well known to be second order in the reduced Cu sites (as also

described by the Authors in the manuscript), the kinetic constants estimated by the Langmuir-type approach are formally flawed.

Response:

This is a thoughtful comment from the reviewer. Our use of the Langmuir-type rate expression follows directly from prior SCR literature, including Krishna et al. (Nature Catalysis, 2023; Journal of Catalysis, 2021) [1, 2] and Jones et al. (Journal of Catalysis, 2020) [3], where the same expression is used to extract global half cycle rate constants from steady-state SCR data on Cu-CHA catalysts with varying Cu-loading across a wide range of O_2 pressures. We fully agree with the reviewer that the intrinsic Cu(I) oxidation step requires two Cu(I) ions, making it second order in Cu(I). As the reviewer mentioned, our transient NO+NH_3-titration, transient SCR experiments and associated kinetic models already take this into account (Note 3 and Figure 5 in supplementary information). In addition, we also performed new additional O_2-pulse experiments that isolates the oxidation half-cycle rate constant and confirmed a second-order dependence on Cu(I) (details provided below). However, the Langmuir-type expression we applied in the steady-state analysis (Fig. 3 in the manuscript) is not meant to represent the elementary Cu(I) kinetics. Its purpose is to describe how the observed macroscopic SCR rate depends on O_2 pressure under steady-state conditions. Krishna et al. has shown that for samples with low Cu-loading, like the ones used in our study, the apparent O_2 reaction order is close to unity [2]. When SCR rates are measured as a function of O_2 pressure, they invariably show a Langmuir-type saturation behavior: at low O_2 pressure the rate increases in a first-order manner because Cu(I) oxidation is rate-limiting, while at high O_2 pressure the rate becomes zero-order because Cu(II) reduction limits the cycle. This transition reflects the continuous shift in the steady-state Cu(I)/Cu(II) distribution with O_2 pressure. The Langmuir expression captures this macroscopic O_2-dependence very well and enables extraction of global oxidation-limited and reduction-limited rate constants. Importantly, this O_2-dependent rate behavior emerges from the changing steady-state Cu oxidation state, not from the intrinsic molecularity of the Cu(I) oxidation step. Therefore, the second-order Cu(I) chemistry revealed in our transient experiments and the Langmuir-type analysis of steady-state SCR rates address two different levels of kinetic information: the former establishes the true stoichiometry of the OHC step, while the latter summarizes how the full catalytic cycle responds

to O_2 pressure. Together, these two approaches are complementary and collectively strengthen our mechanistic interpretation.

To address the reviewer's point, we further performed Cu(I) oxidation measurements designed to isolate the elementary Cu(I) oxidation kinetics based on its 2nd order dependence on Cu. In these experiments (**Figure R1a**), the entire Cu inventory was first reduced to Cu(I) by exposing the catalyst to NO+NH_3 at 200 °C, followed by exposure to O_2 for controlled time periods to oxidize a finite fraction of Cu(I) to Cu(II), and then re-reduced to Cu(I) with NO+NH_3. The amount of NO consumed in these NO+NH_3 titrations are then calculated to quantify the amount of Cu(II) formed during each discrete oxidation period. **Figure R1b** presents such Cu(II) fraction formation as a function of time. These measurements were performed at ANRs 0.5 and 1.25, as the Cu(II) formation rate was higher in the former case than the latter as evident from, **Figure R1b**, indicating a higher Cu(I) oxidation rate at the lower ANR. Such Cu(II) oxidation rate are then fitted to the following rate expression depicting 2nd order dependence on Cu(I):

$$R_{OHC} = k_{OHC} \cdot [O_2] \cdot [Cu(I)]^2$$

Since O_2 is in large excess, [O_2] can be considered a constant. The Cu(I) oxidation rate vs. [Cu(I) fraction]^2 plot is linear as seen in Fig. 1c, confirming the Cu(I) reaction order as 2. The rate constants calculated from the plot are 1.35×10^{-2} s^{-1} at ANR = 0.5, and 1.15×10^{-2} s^{-1} at ANR = 1.25, consistent with the lower oxidation rate at higher the ANR.

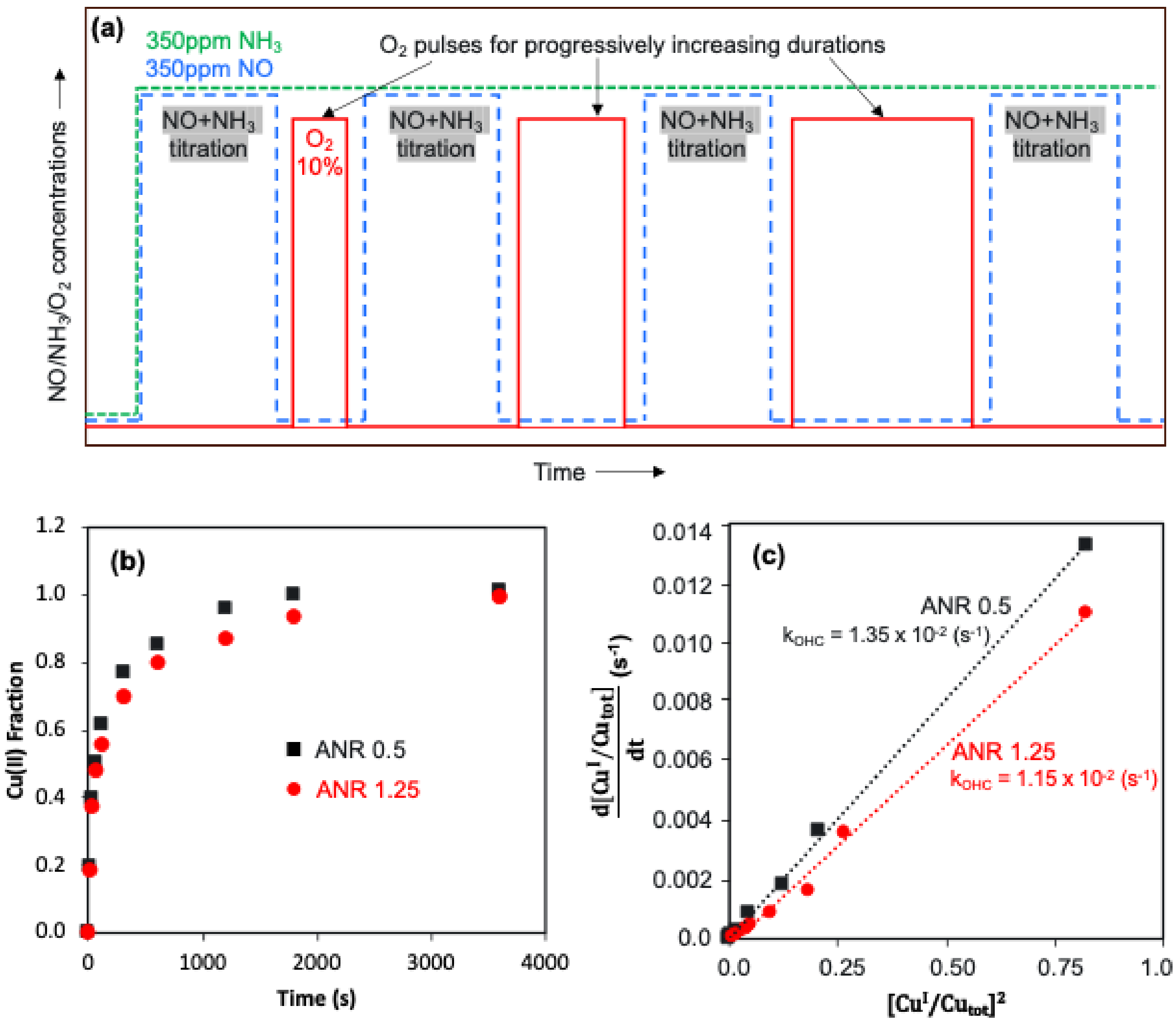

Figure R1: (a) O_2-pulse experimental protocol to probe Cu(I) oxidation rate: Initially, 350 ppm NO + 350 ppm NH_3 is flowed over the catalyst (Cu-0.5) to reduce all Cu sites to Cu(I). NO is then turned off and O_2 is introduced for a designated length of time, following which O_2 is turned off. NO and NH_3 is restarted to reduce the Cu(II) sites formed by O_2-derived oxidation of Cu(I) sites, and by calculating the NO consumed at the step provides the number of Cu(I) sites oxidized by O_2 pulse. This process is repeated with various time durations of O_2 pulses. The Cu(II) fraction vs. oxidation time calculated this way is plotted in Fig. 1b for two ANRs: 0.5 and 1.25. Fig. 1c plots the consumption rate of Cu(I) by O_2-derived oxidation vs. the square of Cu(I) fraction. A linear relationship is obtained indicating a second order dependence of Cu(I) oxidation rate on C(I) concentration.

In conclusion, the intrinsic (transient) and global (steady-state) models probe different levels of mechanistic detail and are complementary rather than contradictory. The transient experiments as well as new O_2-pulse experiments confirm the reviewer's point that OHC is second order in Cu(I), while the steady-state Langmuir analysis allows extraction of apparent rate constants in a manner consistent with global SCR kinetics. Importantly, both approaches lead to the same mechanistic conclusion in our manuscript: NH_3 inhibits SCR primarily by suppressing Cu mobility and Cu-Cu pairing, thereby slowing the OHC step.

3) Analysis of RHC-StdSCR-RHC tests: to estimate the amount of oxidized Cu sites at steady-state NH_3-SCR conditions, in my view the Authors should compute the integral amount of NO converted during the final reduction stage of the test, not during the oxidation transient to StdSCR steady state. During this transient, in fact, Cu sites are being oxidized and reduced at the same time, thus it is impossible to decouple the two contributions.

Response:

We really appreciate the reviewer for this important point. We agree that calculation of NO consumption from the final reduction stage provides a more accurate estimation of the OHC rate constant, and we revised our calculations accordingly. The results are reflected in the revised Figure 4 and Table 1 in the manuscript. Although the OHC activation energies and pre-exponential factors changed slightly (e.g. "E_a" changed from 42 kJ/mol vs 45 kJ/mol at ANR 0.5, "A" changed from 6×10^3 to 4.3×10^3 s^{-1}) due to this modified method, the overall trend and mechanistic interpretations remain the same. We have added the following statement to **Note 3** in the supplementary information to reflect such changes.

"Another RHC transient was collected following this SCR steady state by stopping O_2 flow and allowing only NO and NH_3 to flow. The area under this post-SCR RHC NO transient provides an estimate of the total number of Cu^I sites that get oxidized to Cu^{II} (i.e. $x_{Cu^{II}}$) during steady state SCR."

4) The data for the oxidized Cu sites reported in the description of Figure 7 are probably inverted: "At 200 °C, amount of Cu^I that oxidized to Cu^{II} was calculated to be 0.98 µmols and 2.1 µmols at ANR = 0.5 and 1.25, respectively". Considering the NH_3 inhibition of the OHC, at high ANR a lower Cu^{II} amount should be expected.

Response:

We are thankful to the reviewer for catching this inadvertent mistake. We have corrected it in the revised SI, and the values now reflect Cu(II) oxidized during SCR, as calculated from the final RHC transient (following the reviewers suggestion in Comment #3).

5) The Authors tested also two aged catalyst samples. Do these data reveal the effect of aging on the NH_3 inhibition? Is the NH_3 inhibition stronger over aged catalysts? I think that this aspect should be mentioned in the manuscript.

Response:

Thank you for this valuable comment. The aged catalyst data indeed reveal differences in NH_3 inhibition behavior. The field-aged (FA) catalyst exhibits stronger NH_3 inhibition than the degreened (DG) sample: the FA catalyst begins to show measurable inhibition already at ANR ≈ 0.5, whereas the DG sample requires ANR ≈ 1 for comparable inhibition to appear. This indicates that aging increases the sensitivity of the catalyst to NH_3-rich conditions, likely due to reduced Cu mobility and altered Cu speciation, which make OHC more susceptible to NH_3-driven suppression. Field aging alters the catalyst state in two major routes: it depletes the active Cu inventory via CuO_x cluster formation and Al^{3+} centers via dealumination. While our results in FA sample shows the these two changes ultimately lead to more detrimental NH_3 inhibition, it also important to probe the individual effect of each of these chemical changes. We have already shown that the degree of NH_3-inhibtion is larger on low Cu-loaded catalysts. As a part of this response to the reviewer, we further synthesized a 0.5 wt% Cu-CHA catalyst with a higher Si/Al ratio (Si/Al = 36). Note that all the samples included in the original manuscript had a Si/Al of ~12. Increasing the Si/Al ratio reduces the density of Al sites and increases the average separation between framework charge-compensating positions, resulting in lower Cu mobility. As observed in **Figure R2** below, the Si/Al = 36 sample shows much stronger NH_3 inhibition, with the critical ANR shifting to substantially lower values compared to the original Si/Al = 12 catalyst with 0.5 wt% Cu (i.e. Cu-0.5 sample). This confirms that NH_3 inhibition becomes more pronounced in materials with limited Cu mobility, whether that mobility restriction comes from lower Cu loading, or lower Al^{3+} centers.

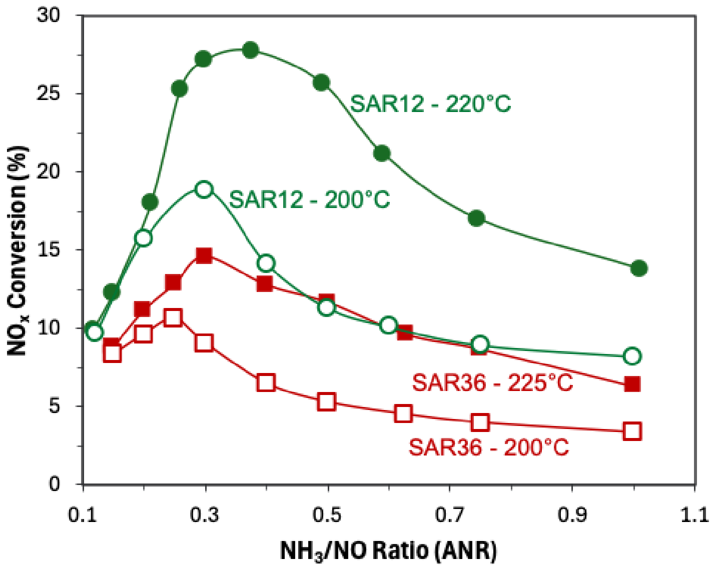

Figure R2: Effect of Si/Al ratio (SAR) on NH_3 inhibition. Figure shows SCR NOx conversion on two samples with 0.5 wt% Cu but varying SARs of 12 and 36, at different temperatures and different ANRs. The sample with SAR36 shows lower NO_x conversion and lower critical ANR for NH_3 inhibition onset, hence a greater degree of NH_3 inhibition at any given temperature.

We had already noted in the beginning of Section 2.1 that the FA sample shows a higher extent of NH_3 inhibition, and we have now added the following paragraph at the end of Section 2.3 and **Supplementary Fig. 21** to reinforce and connect back to that earlier observation, and to clarify that aging not only decreases overall SCR activity but also enhances the extent of NH_3 inhibition.

*"At the beginning of Section 2.1, we discussed how the field-aged (FA) catalyst exhibits a higher extent of NH_3 inhibition than the degreened (DG) sample, with inhibition appearing at a substantially lower ANR. Field aging modifies Cu-CHA catalysts via two primary pathways: partial loss of active Cu through CuO_x cluster formation and depletion of framework Al via dealumination, both of which restrict Cu ion mobility. The effect of reduced active Cu-sites has been already discussed with the help of **Fig. 1**. We independently probed the role of framework Al density by synthesizing a 0.5 wt% Cu-CHA catalyst with a higher Si/Al ratio (Si/Al = 36), compared to the Si/Al ≈ 12 samples used throughout this study. Increasing the Si/Al ratio lowers the density of charge-compensating Al sites and increases their average separation, thereby*

*reducing Cu mobility. As shown in **Supplementary Fig. 21**, the Si/Al = 36 catalyst displays significantly stronger NH_3 inhibition, with the critical ANR shifting to markedly lower values relative to the Si/Al ≈ 12 Cu-0.5 sample. Together with the FA-DG comparison, these results demonstrate that NH_3 inhibition becomes more severe as Cu mobility decreases, whether mobility is restricted by lower Cu loading, reduced Al density, or combined aging effects. This observation reinforces the central conclusion that NH_3 inhibition is governed by mobility-limited oxidation half-cycle kinetics."*

Reviewer #2 (Remarks to the Author):

I co-reviewed this manuscript with one of the reviewers who provided the listed reports. This is part of the Nature Communications initiative to facilitate training in peer review and to provide appropriate recognition for Early Career Researchers who co-review manuscripts.

Reviewer #3 (Remarks to the Author):

The paper addresses a key aspect of the chemistry of the NH_3 inhibition of the Cu chabazite ammonia SCR de-NOx catalyst, namely the ammonia inhibition mechanism which it addresses by a joint computational experimental study.

The work is interesting and relevant; however, some specific details, as given below, are required to further improve the quality of the manuscript.

1) The authors state that ammonia-to-NO_x ratios (ANR) mainly collapse the pre-exponential while E_a changes little, implying an entropic/mobility penalty. Can the authors report $\Delta G^{\ddagger}(T)$ (not only energy barriers) for intercage diffusion of $[Cu^{1+}(NH_3)_2]^+$ / $[Cu^{1+}(NH_3)_3]^+$ with and without a free NH_3 in the cage?

Response:

We thank the reviewer for this helpful suggestion. We have taken into account zero-point energy, internal energy, and entropy contributions to calculate the diffusion barriers of $[Cu_1^+(NH_3)_2]^+$ and $[Cu_1^+(NH_3)_3]^+$ with and without a free NH_3 at 473 K, as shown in **Figure R3**. After applying the thermodynamic corrections, our conclusion remains unchanged: excess NH_3 inhibits the inter-cage diffusion of Cu^I ions in CHA.

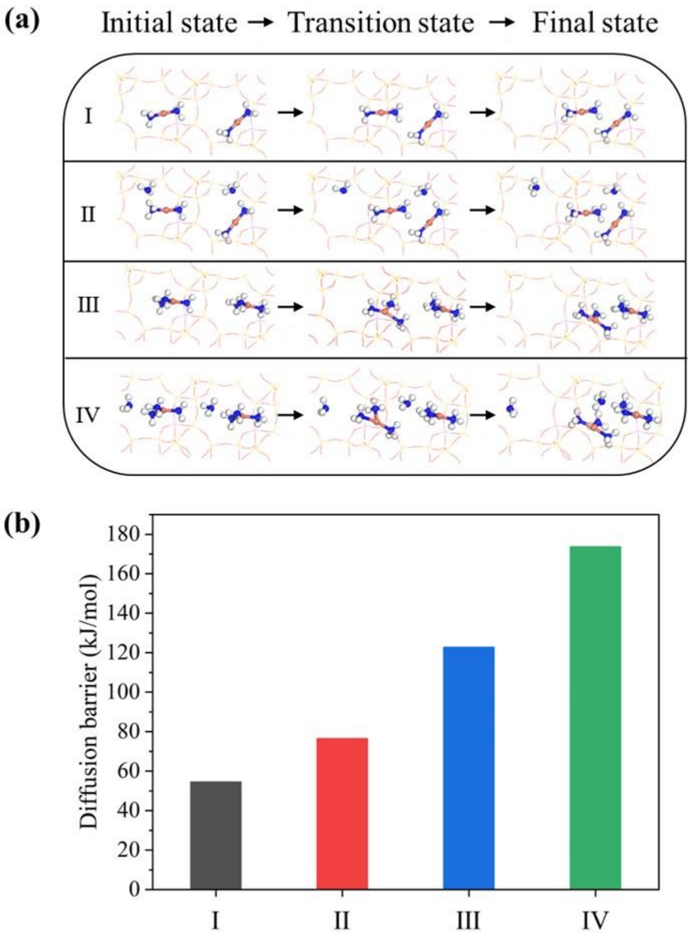

Figure R3. DFT simulation of $Cu^I(NH_3)_x$ (x = 2 or 3) diffusion during SCR on Cu-CHA at 473 K: (a) geometries of the initial state, transition state, and final state of Cu^I ions with different concentrations of ligated and free NH_3, where I: 2 ligated NH_3, II: 2 ligated NH_3, 1 free NH_3, III: 3 ligated NH_3, IV: 3 ligated NH_3, 1 free NH_3. (b) Diffusion barrier of Cu^I ions with different numbers of ligated and free NH_3. Color code: orange (Cu), blue (N), white (H), red (O), yellow (Si), pink (Al).

To clarify, we replaced **Figure 5** with **Figure R3**, and added the following statement in the methods section in supporting information (page 5):

"While calculating the energy barriers, all DFT energies were corrected by including zero-point energy (ZPE), internal energy (ΔU) and entropy (S) contribution, and the Gibbs free energy (G) was obtained by $G = E_{DFT} + ZPE + \Delta U - TS$*. The thermodynamic corrections were performed using the VASPKIT package [4]."*

2) In the DFT, the authors used PBE, but it would be good to see PBE-D3 cross-check to show that the NH_3-crowding trend in diffusion barriers is robust to dispersion treatment.

Response:

We thank the reviewer for raising this point. To examine the influence of the D3 dispersion correction on our conclusions, we compared the diffusion energy barriers of copper ions calculated using the PBE and PBE-D3 functionals. As shown in **Figure R4**, after accounting for long-range interactions, the absolute values of the diffusion barriers for Cu^I ions in all four cases decrease by approximately 5 - 20 kJ/mol. However, this does not alter our qualitative conclusion that excess NH_3 inhibits Cu^I ion diffusion. Therefore, the PBE functional remains appropriate for the present study.

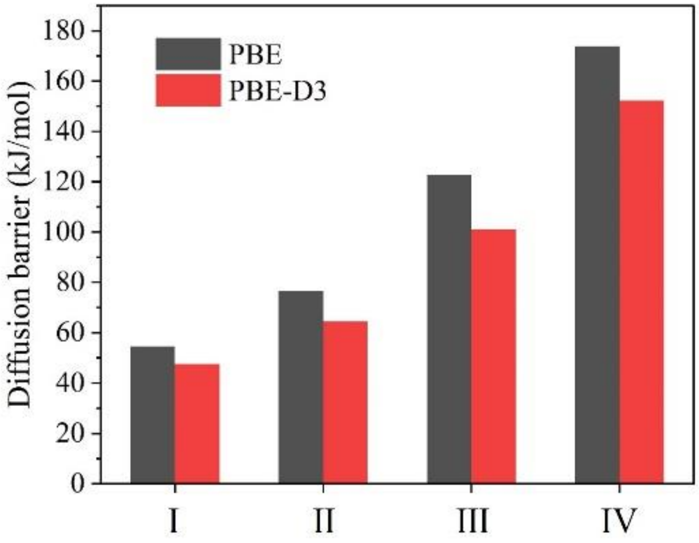

Figure R4. Comparison of $Cu^I(NH_3)_x$ (x = 2 or 3) diffusion barrier using PBE and PBE-D3 functionals. To examine the influence of the D3 dispersion correction [5] on our conclusions, we compared the diffusion energy barriers of copper ions calculated using the PBE and PBE-D3 functionals. The result shows, after accounting for long-range interactions, the absolute values of the diffusion barriers for Cu^I ions in all four cases decrease by approximately 5–20 kJ/mol. However, this does not alter our qualitative conclusion that excess NH_3 inhibits Cu^I ion diffusion. Therefore, the PBE functional remains appropriate for the present study.

To clarify, we added **Supplementary Figure 11** (**Figure R4**) to the manuscript and added the following explanation in the supporting information on page 20:

"To examine the influence of the D3 dispersion correction on our conclusions, we compared the diffusion energy barriers of copper ions calculated using the PBE and PBE-D3 functionals. The result shows, after accounting for long-range interactions, the absolute values of the diffusion barriers for Cu^I ions in all four cases decrease by approximately 5 - 20 kJ/mol. However, this does not alter our qualitative conclusion that excess NH_3 inhibits Cu^I ion diffusion. Therefore, the PBE functional remains appropriate for the present study."

3) The authors emphasise the oxidation half cycle (OHC) throttling under excess NH_3 and attribute it to hindered Cu^+ mobility; however, the present DFT treats only Cu^+ intercage diffusion barriers as NH_3 ligation/free-NH_3 increase, not O_2 binding, O–O activation, or the binuclear Cu–oxo intermediate itself. Can the authors provide O_2-step experiments and DFT of O_2 activation, which will align their emphasis on OHC with a complete demonstration of how O interacts with Cu to convert Cu(I)→Cu(II) under high-ANR, crowded conditions?

The articles below relate to Cu dimer chemistry:

1. Science 357, 898-903 (2017).
2. Chem. Soc. Rev., 2024, 53, 11657-11691

Response:

We thank the reviewer for this thoughtful suggestion. To address the experimental aspect of the comment, we performed O_2-pulse experiments in which fully reduced Cu(I) inventories were exposed to O_2 for progressively increasing durations under different ANR conditions. As shown in **Figure R5** below, the amount of Cu(I) oxidized to Cu(II) during each pulse decreases systematically with increasing ANR from 0.5 to 1.25. This trend directly demonstrates that NH_3-rich conditions suppress the overall oxidation half-cycle, consistent with the kinetic inhibition observed in Section 2.2. These measurements confirm that the net O_2-consuming step of the SCR redox cycle becomes slower as NH_3 ligation increases, in agreement with the hypothesized mobility-limited OHC under high ANR.

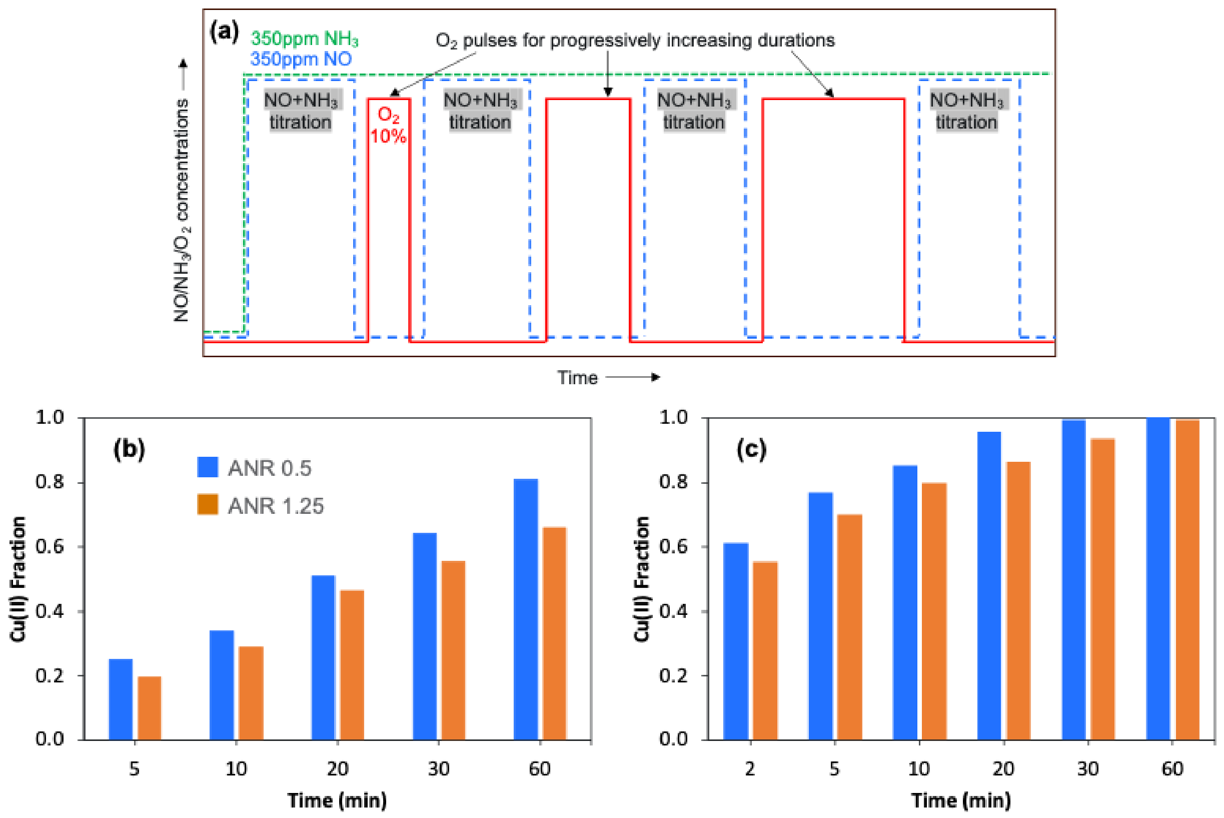

Figure R5: (a) O_2-pulse experimental protocol to probe Cu(I) oxidation rate: Initially, 350 ppm NO + 350 ppm NH_3 is flowed over the catalyst (Cu-0.5) to reduce all Cu sites to Cu(I). NO is then turned off and O_2 is introduced for a designated length of time, following which O_2 is turned off. NO and NH_3 is restarted to reduce the Cu(II) sites formed by O_2-derived oxidation of Cu(I) sites, and by calculating the NO consumed at the step provides the number of Cu(I) sites oxidized by O_2 pulse. This process is repeated with various time durations of O_2 pulses. The Cu(II) fraction vs. oxidation time calculated this way for two ANRs: 0.5 and 1.25, at 180 °C and 200 °C, are plotted in Fig. 3b and Fig. 3c, respectively.

We also performed DFT calculations to investigate O_2 binding and O-O activation after two Cu ions diffuse into the same CHA cage, as shown in **Figure R6**. For $[Cu^+(NH_3)_2]^+$(Case I), the reaction barrier for O_2 binding and O-O activation are 8.73 and 17.08 kJ/mol, respectively. When an additional free NH_3 is present (Case II), the corresponding barriers are 8.37 and 15.15 kJ/mol, indicating that excess free NH_3 has a small effect on O_2 binding and O-O activation on $[Cu_1^+(NH_3)_2]^+$. In contrast, for $[Cu^+(NH_3)_3]^+$(Case III), the barrier for O_2 binding and O-O activation increase to 17.17 and 35.7 kJ/mol, respectively, demonstrating that excess ligated NH_3 inhibits both processes. When an additional free NH_3 molecule is introduced (Case IV), the barriers

further increase to 22.29 and 48.24 kJ/mol, indicating that excess free NH_3 further suppresses O_2 binding and O-O activation on $[Cu^+(NH_3)_3]^+$. However, as shown in the preceding analysis, the diffusion barriers for $[Cu^+(NH_3)_2]^+$ (Case I) and $[Cu^+(NH_3)_3]^+$ (Case III) across the eight-membered ring are 54.9 and 122.54 kJ/mol, respectively, which are significantly higher than the barriers associated with O_2 binding and O-O activation. This indicates that Cu^I ion diffusion remains as the rate-controlling step of the overall reaction.

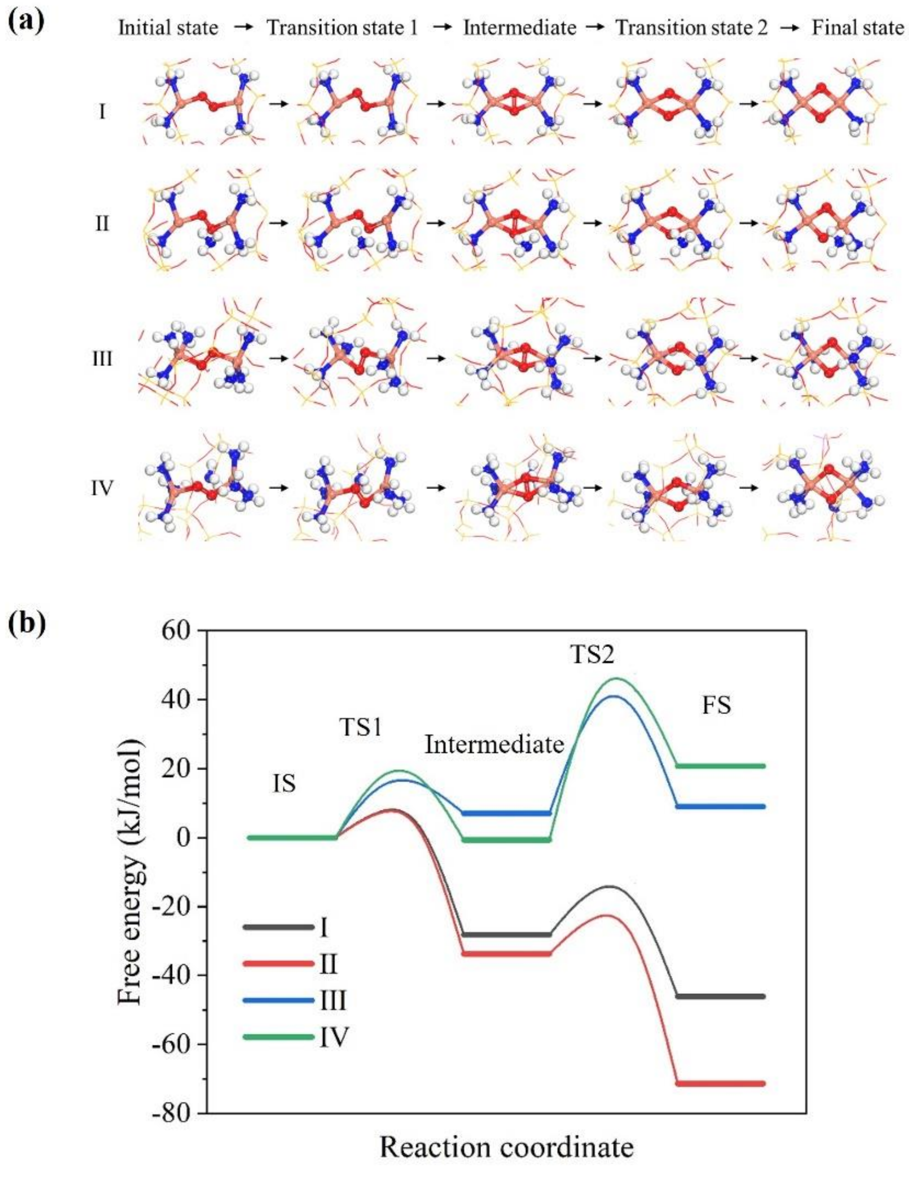

Figure R6. DFT-calculated free energy of O_2 binding (transition state 1) and O-O activation (transition state 2) on $Cu^I(NH_3)_x$ (x = 2 or 3) at 473 K: (a) geometries of the initial state (IS), transition state 1 (TS1), intermediate, transition state 2 (TS2), and final state (FS) of Cu^I ions with different concentrations of ligated

and free NH_3, where I: 2 ligated NH_3, II: 2 ligated NH_3, 1 free NH_3, III: 3 ligated NH_3, IV: 3 ligated NH_3, 1 free NH_3. (b) Reaction barrier of O_2 binding and O-O activation on Cu^I ions with different numbers of ligated and free NH_3.

To clarify, we added **Figure 6** (**Figure R6**) and the following statement in the manuscript on page 21:

*" Since the O_2-driven oxidation half-cycle requires two $[Cu^I(NH_3)_2]^+$ species to come together and form an O_2-bridged dimer, this pairing enables O_2 activation [6, 7]. Because both the dimer formation and the subsequent O_2 activation may be rate-limiting, we next investigate these two elementary steps after the two Cu ions diffuse into the same CHA cage (**Fig. 6**). We also investigate O_2 binding and O-O activation after two Cu ions diffuse into the same CHA cage, as shown in **Fig. 6**. For $[Cu_1^+(NH_3)_2]^+$(Case I), the reaction barrier for O_2 binding and O-O activation are 8.73 and 17.08 kJ/mol, respectively. When an additional free NH_3 is present (Case II), the corresponding barriers are 8.37 and 15.15 kJ/mol, indicating that excess free NH_3 has a negligible effect on O_2 binding and O-O activation on $[Cu_1^+(NH_3)_2]^+$. In contrast, for $[Cu_1^+(NH_3)_3]^+$(Case III), the barrier for O_2 binding and O-O activation increase to 17.17 and 35.7 kJ/mol, respectively, demonstrating that excess ligated NH_3 inhibits both processes. When an additional free NH_3 molecule is introduced (Case IV), the barriers further increase to 22.29 and 48.24 kJ/mol, indicating that excess free NH_3 suppresses O_2 binding and O-O activation on $[Cu_1^+(NH_3)_3]^+$. However, as shown in the preceding analysis (**Figure 5b**), the diffusion barriers for $[Cu_1^+(NH_3)_2]^+$ and $[Cu_1^+(NH_3)_3]^+$ across the eight-membered ring are 54.9 and 122.54 kJ/mol, respectively, which are significantly higher than the barriers associated with O_2 binding and O-O activation, suggesting that Cu^I ion diffusion is the rate-controlling step of the overall reaction.*

In summary, excess NH_3 in $[Cu_1^+(NH_3)_3]^+$ modestly increases the barriers for O_2 binding and O-O activation but these barriers remain substantially lower than the corresponding diffusion barriers across the 8-membered ring. This indicates that Cu^I ion diffusion, which is strongly hindered by excess NH_3 through increased ligation and steric bulk, is the dominant rate-controlling step governing Cu dimer formation and, consequently, the overall SCR activity."

We also added the following statement in the supporting information on page 5:

"The climbing image nudged elastic band (CI-NEB) and dimer methods [8] were used to search for the transition state of O_2 binding and O-O activation, which was further verified by calculating the vibrational frequencies confirming that a single imaginary frequency was obtained."

4) The authors discuss that excess reactants (NO, H_2O, O_2) can raise $[Cu^{1+}(NH_3)_2]^+$ diffusion barriers. Authors should include one explicit co-adsorbate case to verify this claim.

Response:

In the Introduction, we mentioned that excess reactants (NO, H_2O, O_2) may increase the diffusion barriers of $[Cu^{1+}(NH_3)_2]^+$, which is based on conclusions reported in the literature [9]; no corresponding experiments were performed in the present work. To address the reviewer's concern, we carried out DFT calculations to evaluate the diffusion barrier of $[Cu^{1+}(NH_3)_2]^+$ in the presence of an additional H_2O. As shown in **Figure R7**, the diffusion barrier increases by approximately 9 kJ/mol, compared with the case without H_2O, indicating that extra water inhibits $[Cu^{1+}(NH_3)_2]^+$ diffusion. Since this work mainly focuses on the inhibition effect from excess NH_3 rather than other reactants, we don't make any modifications regarding this point in our work.

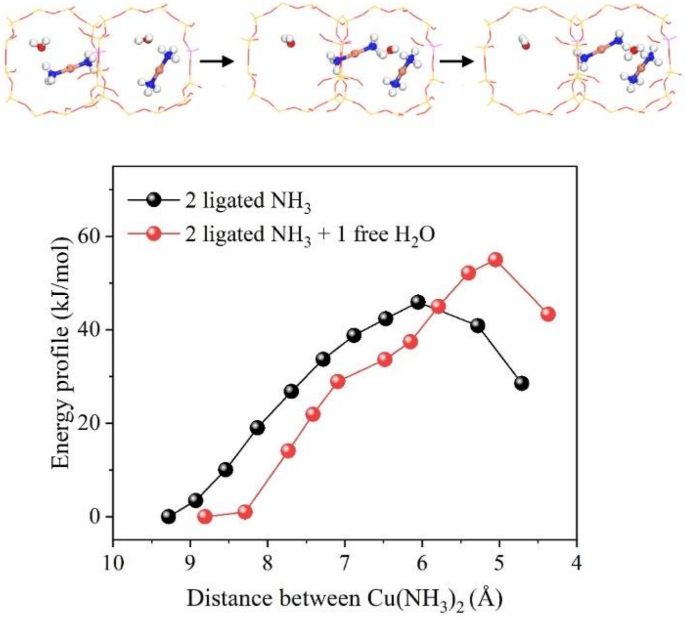

Figure R7: Diffusion barrier for $[Cu^{I}(NH_3)_2]^+$ in presence and absence of a free H_2O molecule

5) Minor comments: The colour code "white" should be for H, not for O in Figure 5. e.g. Colour code: orange (Cu), blue (N), white (O), yellow (Si), pink (Al).

Response:

We appreciate the reviewer for raising this point. We have addressed the typo.

Reviewer #4 (Remarks to the Author):

The manuscript "Insights into the mechanisms of NH_3 inhibition on Cu-CHA SCR catalysts" investigates a copper substituted chabazite zeolite with different copper concentrations. The catalytic system has already been heavily investigated in literature. The focus here is to gain more information about the onset conditions and the degree of inhibition on the SCR reaction by excess ammonia.

The authors take a very systematic approach. They use kinetic modelling to unravel the rate coefficients of both the reduction half cycle and of the oxidation half cycle at different ratios of ammonia to nitrogen oxide and different temperatures. To achieve this, they use both steady state and transient conversion data. The most important finding is the change in the pre-exponential factor of the oxidation half cycle as excess of ammonia is present. Since the evidence points towards limited diffusion of reduced copper species, a theoretical (DFT) investigation is carried out to determine the diffusion of different Cu(I) ammine species. It is concluded that the mobility of Cu(I) in the cages which is necessary for the low temperature oxidation half cycle is severely lessened as the third ammonia attaches to Cu. A large part of the manuscript is devoted to an operando EPR investigation to follow the Cu(II) species at steady state SCR conditions at different temperatures and quantify the ratio between Cu(I) and Cu(II).

Separately, each of the investigations provides a few new insights but not significant progress. Taken all together, **they provide compelling evidence** of exactly where the ammonia inhibition begins to limit the SCR activity. The comprehensive data collection on the same sample series allows better comparisons of the evidence across methods that each have different strengths and weaknesses.

In total, **the manuscript gives a solid foundation** for describing the ammonia inhibition of the

SCR reaction on low concentration Cu Chabazite. The conclusions are also extended towards higher concentrations of copper. **The manuscript is publishable with minor revisions** as given below. The novelty level is borderline for Nature Communications.

The transient kinetic experiments start with Cu fully saturated with ammonia. This raises two concerns:

1) It has previously been described that copper can be autoreduced in ammonia atmosphere at some of the higher temperatures investigated if left long enough. Was the rate of autoreduction determined and deemed irrelevant for the kinetic experiments?

Response:

We thank the reviewer for this important comment. To determine whether Cu autoreduction (sample: Cu-0.5, which has 0.5 wt% Cu and a Si/Al of 12) in NH_3 could influence our kinetic measurements, we performed steady state NH_3 oxidation and dedicated NO+NH_3 titration experiments comparing the total reducible Cu inventory after NH_3+O_2 exposure versus after a 1-hour NH_3-only hold. In the baseline protocol, the catalyst was first oxidized under NH_3+O_2, after which O_2 was removed and NO+NH_3 was introduced; the integrated NO consumption reflects the total Cu(II) concentration, given the 1:1 NO/Cu(II) reduction stoichiometry. We then repeated this procedure but inserted an intermediate 1-hour NH_3-only exposure step. Any Cu autoreduction during this NH_3-only period would be detected as a lower NO uptake.

As shown in **Figures R8b-d** below, the titration curves at 200 °C are identical, demonstrating no detectable Cu autoreduction under NH_3-only conditions. At 300 °C, the difference is minimal and within experimental uncertainty. Only at 450 °C do we observe a significant decrease in NO uptake (6.76×10^{-7} mol), corresponding to ~20% Cu(II) reduction (out of 3.38×10^{-6} mol total Cu). These results indicate that Cu autoreduction becomes appreciable only at high temperatures.

Furthermore, **Figure R8a** below shows that NH_3 oxidation does not light off until ≈250 °C (feed: 350 ppm NH_3, 10% O_2, 6% H_2O, 120,000/h space velocity) which confirms that NH_3 remains largely unreactive at lower temperatures and therefore cannot act as a reductant in this regime. This observation fully aligns with the titration measurements and provides independent evidence that autoreduction is negligible at temperatures below 250 °C.

Since all kinetic measurements relevant to NH_3 inhibition in our study were performed between 150–250 °C, where both NH_3 oxidation and NH_3-driven Cu autoreduction are absent for Cu-0.5, we conclude that autoreduction does not influence our kinetic analysis or mechanistic interpretation.

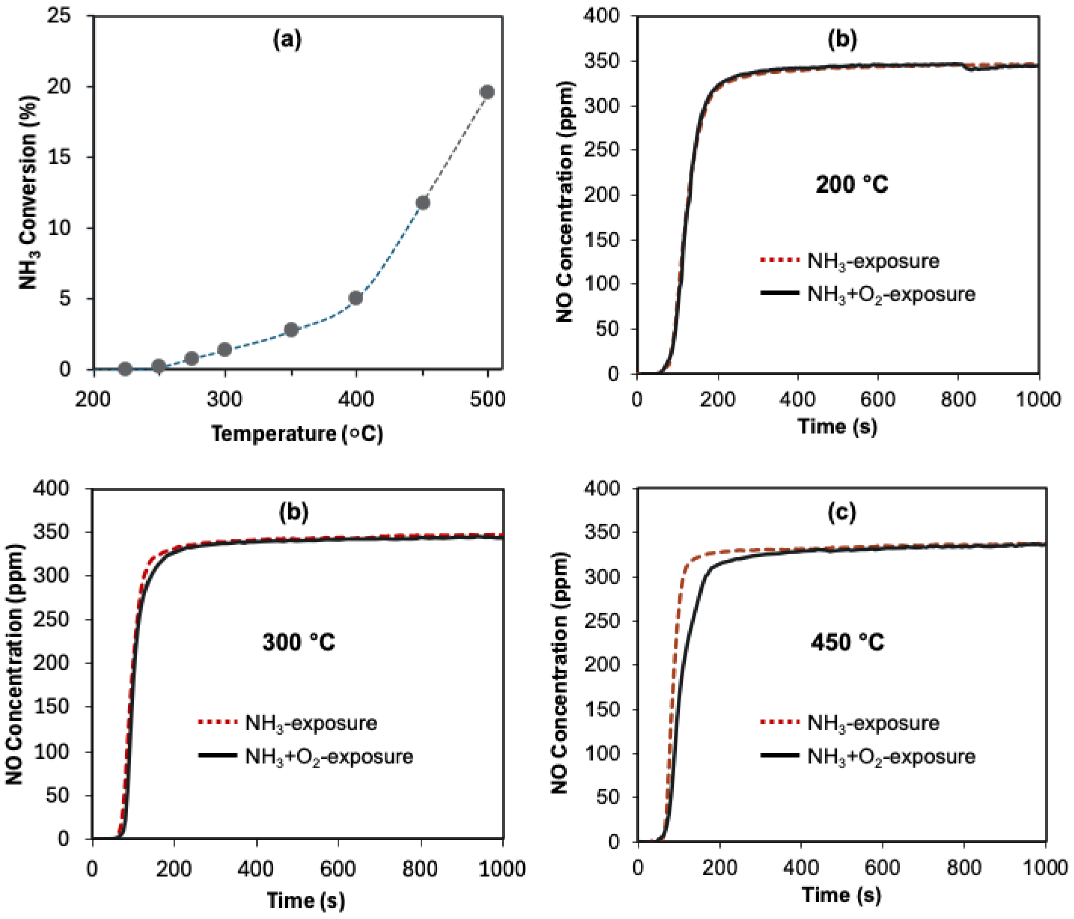

Figure R8. (a) NH_3 oxidation on Cu-0.5 sample (feed: 350 ppm NH_3, 10% O_2, 6% H_2O, 120,000/h space velocity). NO transients obtained during NO+NH_3 titration of Cu-0.5 at (b) 200 °C, (c) 300 °C and (d) 450 °C. Each of these figures contains two NO transients: solid black line presents NO transient from NO+NH_3 titration after NH_3+O_2 exposure of the catalyst, while dashed red line presents NO transient from titration after 1-hour NH_3 exposure (in absence of O_2) of the catalyst.

2) The equilibrium state for a sample fully saturated in ammonia is likely to have all Cu at a specific, large dispersion level in the zeolite cages. Cu will have to move the maximal amount from this state to form a dimer in the oxidation half cycle. If a sample is left at SCR conditions, the mean Cu-Cu distance before oxidation is likely always lower, since some copper will not have

had time to disperse fully. This might have an impact on the kinetics not observed in this experiment.

Response:

We appreciate the reviewer's insightful comment regarding the Cu-Cu distribution in a fully NH_3-saturated state versus the dynamically sampled Cu distribution under steady SCR conditions. To directly evaluate whether the initial level of Cu dispersion affects the observed oxidation half-cycle kinetics, we performed O_2-pulse OHC experiments starting from a fully reduced catalyst, a state that closely represents the maximally dispersed Cu(I) configuration described by the reviewer. In this protocol, the catalyst is first driven completely to Cu(I) using NO+NH_3, ensuring the highest achievable Cu(I) dispersion, after which it is exposed to O_2 for controlled time intervals. The subsequent NO+NH_3 titration quantitatively measures the Cu(II) formed during each discrete O_2 pulse.

These experiments (**Figure R9**) show that even when starting from the fully dispersed Cu(I) state, the extent of Cu(I) oxidation decreases with increasing ANR, demonstrating that NH_3-rich environments inhibit the OHC regardless of the initial Cu-Cu distance. This confirms that the dominant kinetic limitation arises from an increase in the Cu(I) migration/pairing barrier, not from differences in the initial Cu spatial distribution.

Importantly, the manuscript also reports OHC inhibition under steady SCR conditions, where the Cu population samples a more dynamic ensemble of positions with shorter average Cu-Cu distances than in the fully dispersed NH_3-saturated limit. The fact that OHC inhibition is observed in both cases, (i) the fully dispersed Cu(I) initial state and (ii) the dynamically relocalizing SCR state, demonstrates that our mechanistic conclusions are robust across the relevant Cu distributions.

Therefore, although the equilibrium NH_3-saturated state may correspond to maximal Cu dispersion, our experiments explicitly show that the key kinetic effect (lower Cu oxidation at higher ANR) is unchanged whether we begin from the maximally dispersed Cu(I) configuration or from the dynamically sampled configuration under SCR. We have added this clarification to the revised manuscript (**Supplementary Fig. 13**).

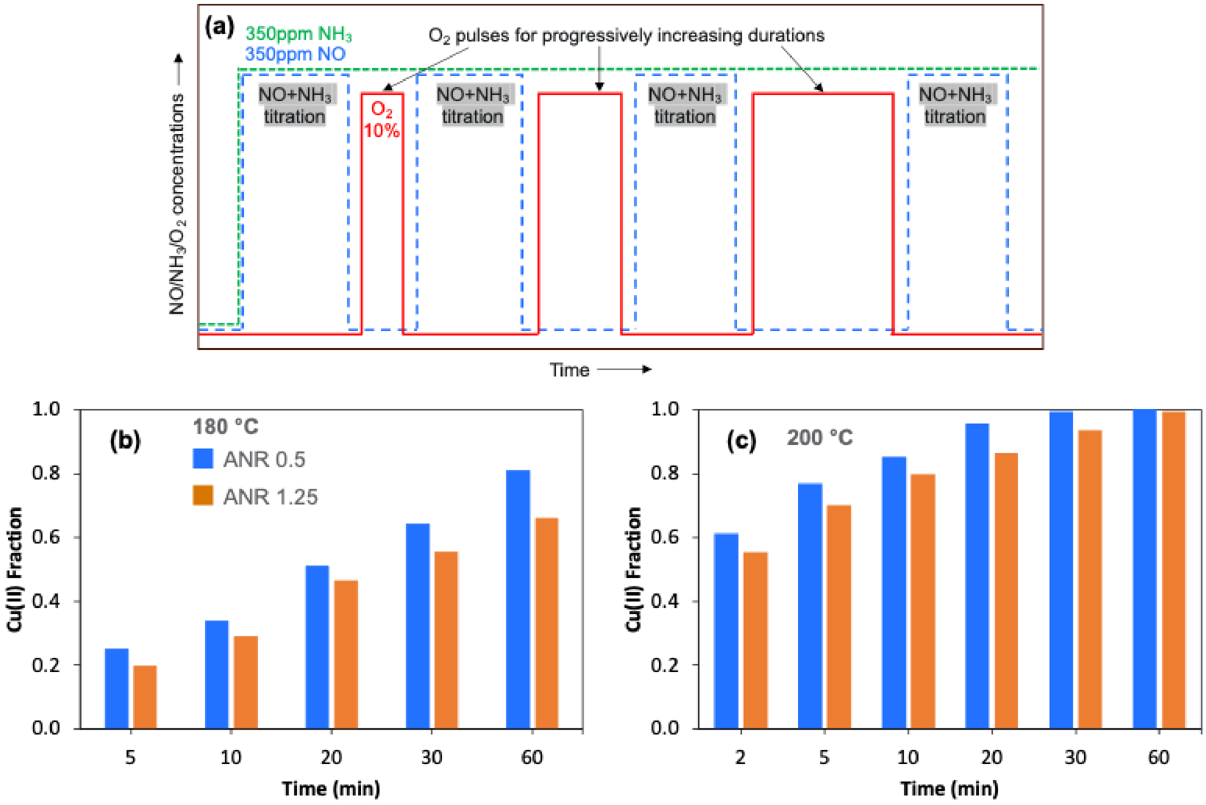

Figure R9: (a) O_2-pulse experimental protocol to probe Cu(I) oxidation rate: Initially, 350 ppm NO + 350 ppm NH_3 is flowed over the catalyst (Cu-0.5) to reduce all Cu sites to Cu(I). NO is then turned off and O_2 is introduced for a designated length of time, following which O_2 is turned off. NO and NH_3 is restarted to reduce the Cu(II) sites formed by O_2-derived oxidation of Cu(I) sites, and by calculating the NO consumed at the step provides the number of Cu(I) sites oxidized by O_2 pulse. This process is repeated with various time durations of O_2 pulses. The Cu(II) fraction vs. oxidation time calculated this way for two ANRs: 0.5 and 1.25, at 180 °C and 200 °C, are plotted in Fig. 5b and Fig. 5c, respectively.

3) Experimental details for the EPR measurements are missing. What are the instrument settings and, importantly, the frequency. The field values are not a useful measure on their own, since they will deviate from instrument to instrument.

Response:

We now include experimental procedure for EPR measurements on page 6 of the original supplementary information file. We have revised the section to include the following additional details.

"EPR spectra were collected with a Bruker 580 Elexsys equipped with a SHQE resonator. The sample temperature was maintained with a Bruker ER4131VT nitrogen temperature control system. Typically, the microwave frequency was 9.3 GHz at 0.2 mW power. The field was swept 1200 G in 84 seconds with 5 G field modulation and 82 ms of time constant. For ex situ measurements, the samples were contained in 4 mm OD quartz tubes (Wilmad). For in situ measurements the samples were held in a laboratory built coaxial plug flow reactor consisting of an inner 3 mm tube that contained the sample (~15 mg) between plugs of quartz wool. An outer 5 mm tube carried the gas in a cross-flow manner to preheat the reactant gases to the desired temperature before contacting the sample. Total gas flow was 100 ml/minute, with the mixture controlled by four Brooks computer controlled flow meters."

4) The EPR spectra almost all show a clear line at 3330 G, which is marked, but not explained. Is it an impurity in the zeolite support or is it a relevant signal?

Response:

The 3330 G feature is not relevant to our samples. It appears in all spectra, irrespective of temperature or reaction conditions, and its absolute intensity remains constant in all spectra. As a result, its intensity relative to the Cu signal increases when Cu(II) fraction decreases, for example at high ANRs in Fig. 7 in the manuscript. These observations tell us that the 3330 G peak is an interference from the instrumental setup.

To confirm this, we collected EPR spectra of the empty sample tube at room temperature before and after heating it to 300°C. We also collected spectra of Cu-0.5 sample at room temperature, following heat treatment at 300°C. **Figure R10a** below shows these three spectra. As expected, the empty tube spectra is a flat line when compared to the Cu-0.5 spectra. All three spectra, however, contain a feature at around 3330 G, the same one that the reviewers' comment addresses. **Figure R10b** provides an enlarged scale focusing on the 3330 G peak for ease of observation. These results thus confirms that the 3330 G peak comes from the instrument itself and hence is present in all in-situ and ex-situ data presented in this paper.

We have added these clarifications in the revised manuscript and **Supplementary Note 6** and **Fig. 14.** We also added the following sentence in the first paragraph of section 2.3:

"The 3330 G signal is an instrumental artifact, and hence is not associated to any catalytically relevant process (see details in Supplementary Note 6 and Fig. 14)."

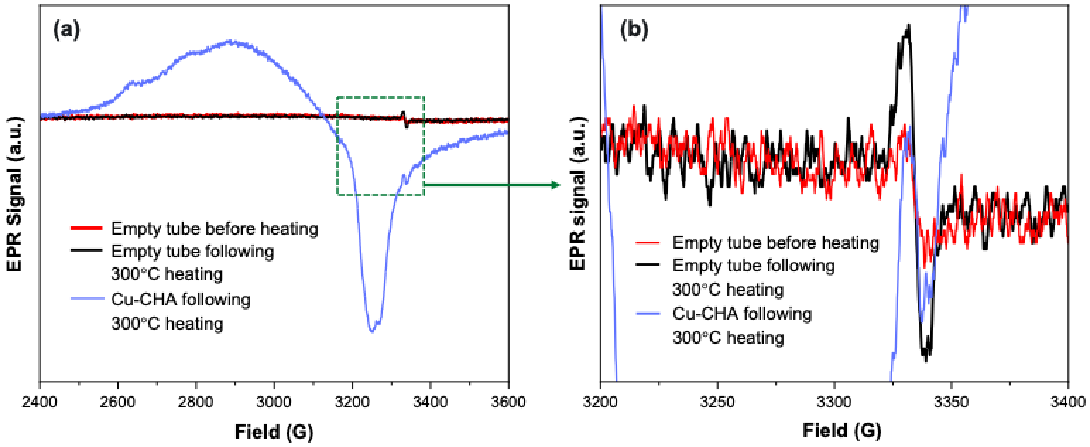

Figure R10: Continuous wave EPR spectra (a) 2400-3600 G region, (b) 3200-3400 G region. The figures present spectra of three samples- (1) empty sample tube at room temperature, (2) empty tube at room temperature, but after heating it to 300°C and cooling down to room temperature, (3) Cu-0.5 sample at room temperature, but after heating it to 300°C and cooling down to room temperature.

5) The analysis of EPR spectra is of a descriptive nature. It would have been beneficial to attempt a more quantitative approach to the data analysis. Is it possible to give an indication of how much of each type of EPR active species is present?

Response:

We thank the reviewer for this constructive suggestion. In response, we have extended the EPR analysis beyond a descriptive comparison and now provide a semi-quantitative determination of the relative populations of the dominant EPR-active Cu(II) species by explicitly fitting the operando spectra using a two-site model.

In brief, continuous-wave EPR spectra (first-derivative mode) were fitted using a custom Python routine in which each spectrum was represented as the sum of one to three axial Cu(II) components ($I = 3/2$). Each component was parameterized by an axial g-tensor ($g_{\perp}, g_{\parallel}$), an axial hyperfine tensor ($A_{\perp}, A_{\parallel}$, reported in MHz), and a Lorentzian linewidth (γ, in Gauss). Powder-averaged lineshapes were generated by numerical integration over molecular orientations and

summation over the four Cu hyperfine lines ($m_I = \pm3/2, \pm1/2$). To account for inhomogeneous broadening, g-strain and A-strain terms were included for each component.

The overall spectrum was modeled as a linear combination of site contributions with positive fractions constrained to sum to unity. During fitting, a small field-axis alignment (global shift and scale) and a small microwave-frequency adjustment were allowed. A low-order polynomial baseline (up to quadratic) was included and optimized simultaneously with the spectral parameters. Best-fit parameters were obtained by nonlinear least-squares minimization with bounded constraints.

The operando EPR spectra at each ANR and temperature were fitted using two Cu(II) components:

(i) Site 1, with $g_\parallel \approx 2.34$ and $A_\parallel \approx 135$ G, assigned to Cu(II) species with dominantly Cu-O_L coordination (Z_2Cu-type sites); and

(ii) Site 2, with $g_\parallel \approx 2.27$ and $A_\parallel \approx 155$ G, assigned to Cu(II) species with mixed Cu-N/Cu-O_L coordination.

The relative contribution of each site was obtained from the integrated intensity of the fitted components.

At 220 °C, the fitted site distributions show a strong ANR dependence: at ANR = 0.125, Site 1 accounts for ~76 % of the EPR-active Cu(II), with the remaining ~24 % as Site 2. At ANR = 0.25, the Site 1 fraction drops sharply to ~10 %, and for ANR ≥ 0.5 the spectra are fully dominated by Site 2. At 180 °C, Site 1 contributes ~44 % at ANR = 0.125, while Site 2 contributes ~56 %; for ANR ≥ 0.25, Site 2 accounts for essentially 100 % of the EPR-active Cu(II). Representative fits at 220 °C and 180 °C are provided in **Figures R11a-h** and **R12a-h**, respectively, and the resulting site distributions are summarized in **Figure R13**.

This quantitative trend demonstrates that increasing NH_3 availability systematically shifts the Cu(II) population from Cu-O_L-dominated sites toward NH_3-coordinated Cu(II) species, with the transition occurring at lower ANR at lower temperature. While it is difficult to obtain absolute Cu speciation from CW-EPR due to overlapping signals and EPR-silent Cu species, this two-site fitting approach yields a robust semi-quantitative measure of the relative Cu-O_L versus Cu-N/Cu-

O$_L$ populations and directly links changes in Cu coordination to the observed NH_3-dependent SCR kinetics. We have incorporated this analysis and discussion into the revised manuscript and **Supplementary Note 7** and **Supplementary Figs. 15-17**.

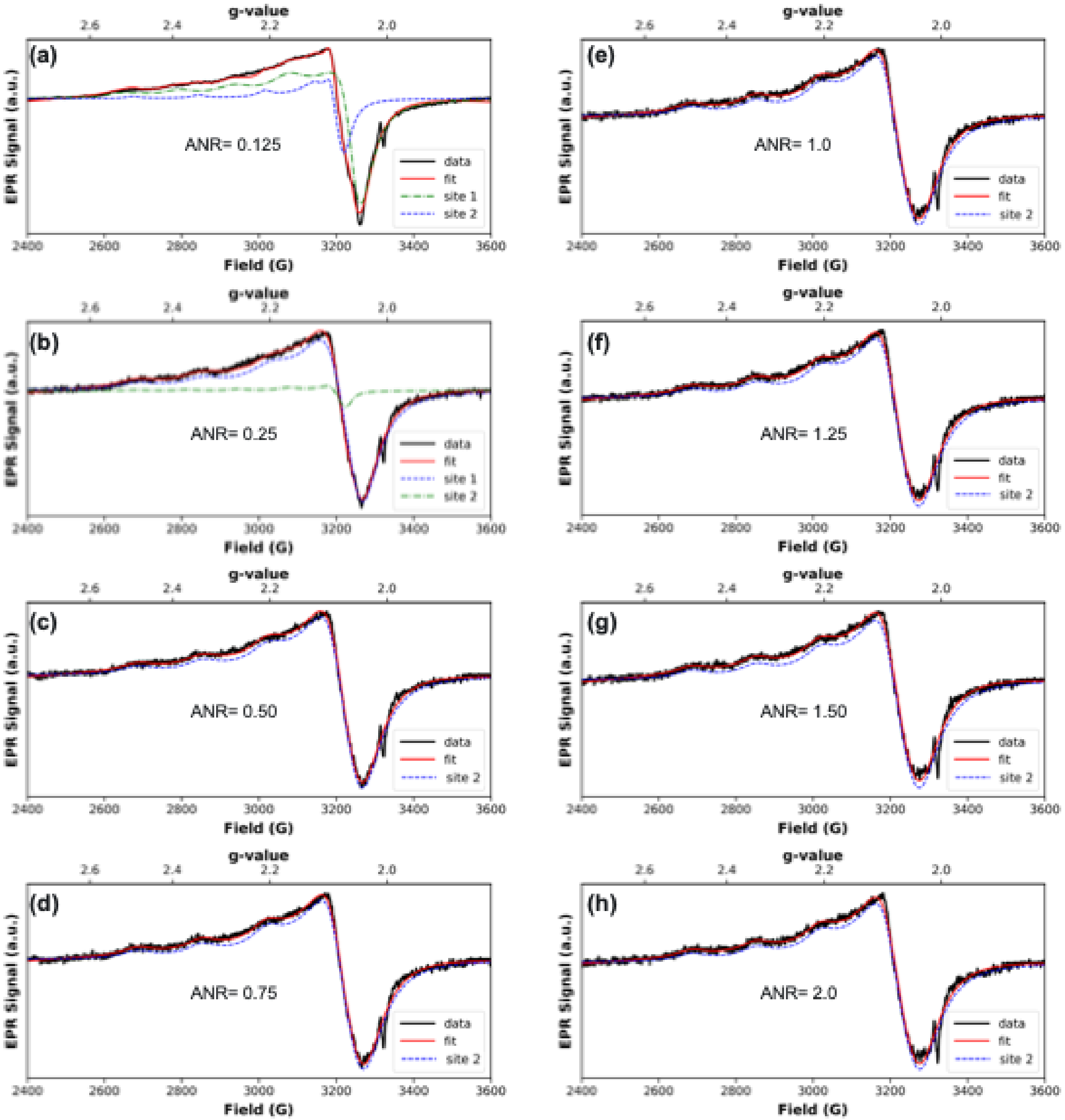

Figure R11: Two-site fitting of operando EPR spectra under SCR conditions. Operando CW-EPR spectra of Cu-0.5 acquired at **220 °C** under steady-state standard SCR conditions (500 ppm NO, varying NH_3, 10 % O_2, 3 % H_2O, SV = 400 k h^{-1}) and different ANRs = 0.125–2.0). Black traces show experimental data, while colored curves represent the best fits obtained using a two-site model consisting of Site 1 ($g_\parallel \approx 2.33$, $A_\parallel \approx 130$ G; Cu–O$_L$-dominated coordination) and Site 2 ($g_\parallel \approx 2.27$, $A_\parallel \approx 155$ G; mixed Cu–N/Cu–O

coordination). The relative contributions of the two sites were determined from the integrated intensity of the fitted components and are summarized in Figure R13.

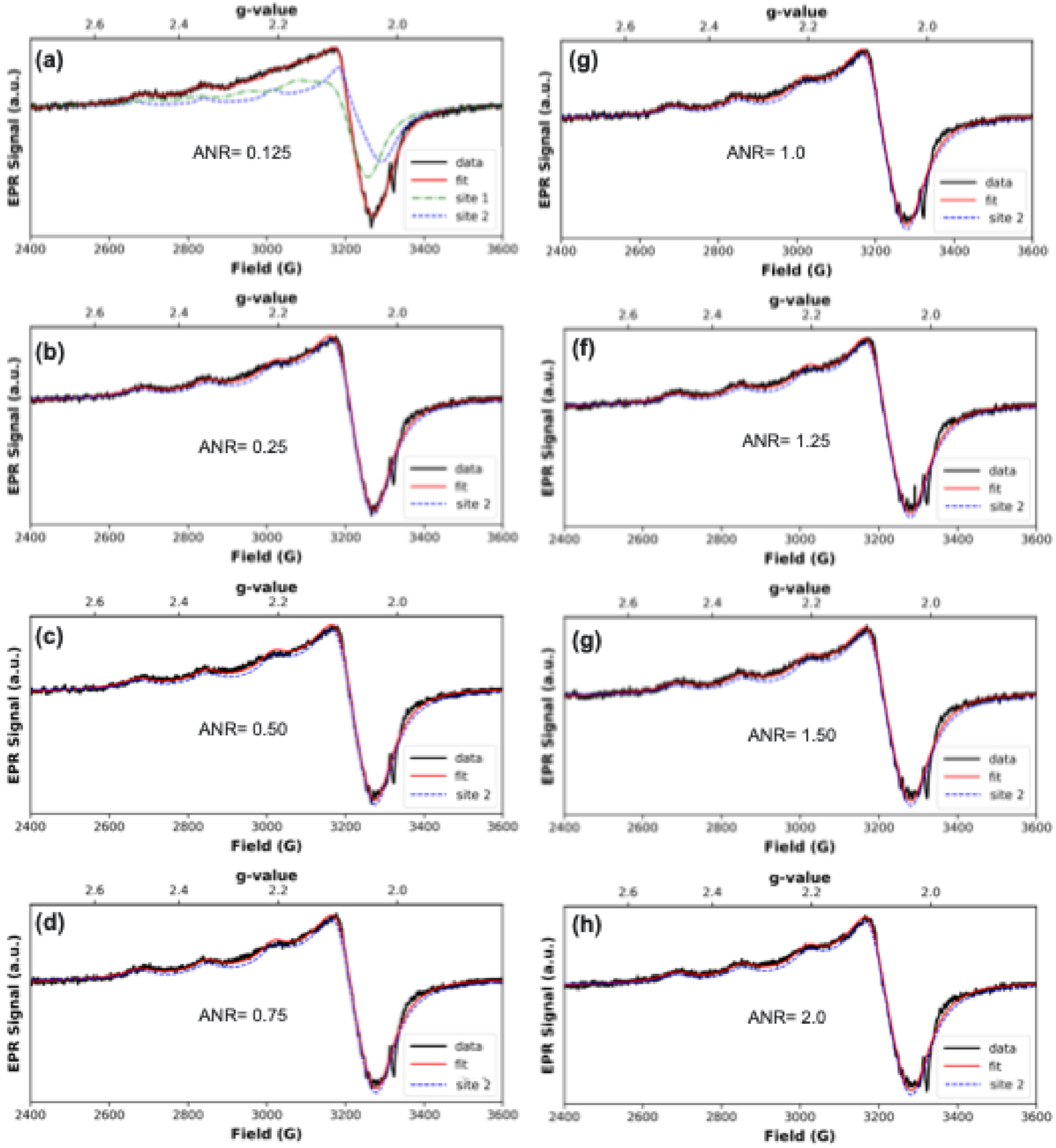

Figure R12: Two-site fitting of operando EPR spectra under SCR conditions. Operando CW-EPR spectra of Cu-0.5 acquired at **180 °C** under steady-state standard SCR conditions (500 ppm NO, varying NH_3, 10 % O_2, 3 % H_2O, SV = 400 k h^{-1}) and different ANRs = 0.125–2.0). Black traces show experimental data, while colored curves represent the best fits obtained using a two-site model consisting of Site 1 ($g_\parallel \approx 2.33$, $A_\parallel \approx 130$ G; Cu–O_L-dominated coordination) and Site 2 ($g_\parallel \approx 2.27$, $A_\parallel \approx 155$ G; mixed Cu–N/Cu–O

coordination). The relative contributions of the two sites were determined from the integrated intensity of the fitted components and are summarized in Figure R13.

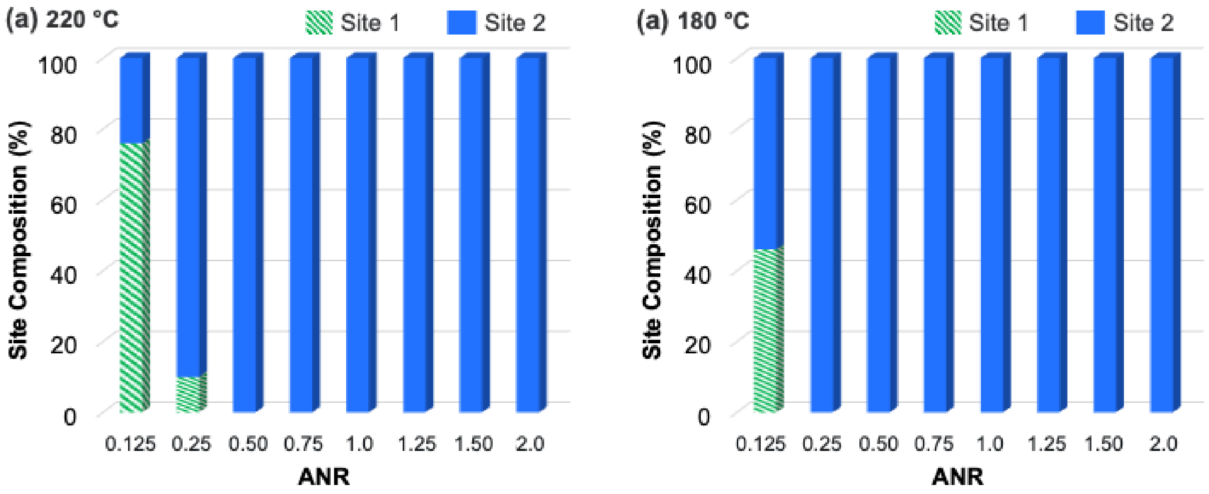

Figure R13: Distribution of EPR-active Cu(II) species as a function of ANR. Relative fractions of Site 1 (Cu–O_L-dominated coordination; $g_{\parallel} \approx 2.33$, $A_{\parallel} \approx 130$ G) and Site 2 (mixed Cu–N/Cu–O coordination; $g_{\parallel} \approx 2.27$, $A_{\parallel} \approx 155$ G), obtained from two-site fitting of operando EPR spectra of Cu-0.5 under steady-state SCR conditions at (a) 220 °C and (b) 180 °C. The site fractions were calculated from the integrated intensity of the fitted components shown in Figures 7 and 8. Increasing ANR leads to a progressive shift from Cu–O_L-dominated sites to NH_3-coordinated Cu(II) species, with the transition occurring at lower ANR at lower temperature.

We have added Supplementary Note 7 to the revised SI reflecting these new analyses. In addition, we also added the following statements to the text related to Fig. 8 in the revised manuscript:

"These two features were used to fit each EPR spectrum, and the detailed method and results are described in Supplementary Note 7 and Supplementary Figs. 15, 16 and 17. The fitting results show that tThe first feature dominates at higher ANR, while the second is more prominent at the lowest ANR."

"These trends of Cu-N/Cu-O coordination as a function of ANR as inferred from superhyperfine features agree well with those obtained from hyperfine parameters and fit results shown in Figs. 8a and Supplementary Figs. 15-17."

Reviewer #5 (Remarks to the Author):

I co-reviewed this manuscript with one of the reviewers who provided the listed reports. This is part of the Nature Communications initiative to facilitate training in peer review and to provide appropriate recognition for Early Career Researchers who co-review manuscripts.

References:

[1] S.H. Krishna, A. Goswami, Y. Wang, C.B. Jones, D.P. Dean, J.T. Miller, W.F. Schneider, R. Gounder, Influence of framework Al density in chabazite zeolites on copper ion mobility and reactivity during NO x selective catalytic reduction with NH3, Nature Catalysis, 6 (2023) 276-285.

[2] S.H. Krishna, C.B. Jones, R. Gounder, Temperature dependence of Cu (I) oxidation and Cu (II) reduction kinetics in the selective catalytic reduction of NOx with NH3 on Cu-chabazite zeolites, Journal of Catalysis, 404 (2021) 873-882.

[3] C.B. Jones, I. Khurana, S.H. Krishna, A.J. Shih, W.N. Delgass, J.T. Miller, F.H. Ribeiro, W.F. Schneider, R. Gounder, Effects of dioxygen pressure on rates of NOx selective catalytic reduction with NH3 on Cu-CHA zeolites, Journal of catalysis, 389 (2020) 140-149.

[4] V. Wang, N. Xu, J.-C. Liu, G. Tang, W.-T. Geng, VASPKIT: A user-friendly interface facilitating high-throughput computing and analysis using VASP code, Computer Physics Communications, 267 (2021) 108033.

[5] S. Grimme, J. Antony, S. Ehrlich, H. Krieg, A consistent and accurate ab initio parametrization of density functional dispersion correction (DFT-D) for the 94 elements H-Pu, The Journal of chemical physics, 132 (2010).

[6] C. Paolucci, I. Khurana, A.A. Parekh, S. Li, A.J. Shih, H. Li, J.R. Di Iorio, J.D. Albarracin-Caballero, A. Yezerets, J.T. Miller, Dynamic multinuclear sites formed by mobilized copper ions in NO x selective catalytic reduction, Science, 357 (2017) 898-903.

[7] J.A. Nasir, A.M. Beale, C.R.A. Catlow, Understanding deNO x mechanisms in transition metal exchanged zeolites, Chemical Society Reviews, (2024).
[8] G. Henkelman, B.P. Uberuaga, H. Jónsson, A climbing image nudged elastic band method for finding saddle points and minimum energy paths, The Journal of chemical physics, 113 (2000) 9901-9904.
[9] R. Millan, P. Cnudde, V. van Speybroeck, M. Boronat, Mobility and Reactivity of Cu(+) Species in Cu-CHA Catalysts under NH(3)-SCR-NOx Reaction Conditions: Insights from AIMD Simulations, JACS Au, 1 (2021) 1778-1787.

Comment from Reviewer-1:

First, I congratulate the authors for their extensive work in addressing the reviewers' comments. I was truly impressed by the amount of new data and analysis added to the manuscript and to the supplementary material, which clarifies many of the questions raised. However, I still have some clarifications to ask regarding the authors' reply to question number 2 in my previous review.

In their rebuttal, the authors claim that the second order Cu(I) OHC kinetics established the true stoichiometry of the OHC step, while the first-order Langmuir-type analysis of steady-state SCR rates summarizes how the full catalytic cycle responds to O_2 pressure. While it is clear that these two approaches are used to address two different levels of kinetic information, the authors do not clarify this discrepancy in the main manuscript: if not fully explained, analyzing STD-SCR activity data with first order RHC and OHC kinetics and, immediately after, modelling RHC and OHC transients with second order kinetics is confusing and contradictory.

I think that, for the sake of clarity, the authors should clearly address this point in the main text. As they write in the Reviewer Response: "the Langmuir-type expression we applied in the steady-state analysis (Fig. 3 in the manuscript) is not meant to represent the elementary Cu(I) kinetics. Its purpose is to describe how the observed macroscopic SCR rate depends on O_2 pressure under steady-state conditions. Krishna et al. has shown that for samples with low Cu-loading, like the ones used in our study, the apparent O_2 reaction order is close to unity".

Furthermore, the authors performed OHC transient tests to isolate the elementary Cu(I) oxidation kinetics based on its 2nd order dependence on Cu. However, the kinetic analysis of these new data (reported in the Reviewer Response) was not added to the manuscript. Since reduction transients and their modeling are already included, I think that this OHC kinetic analysis will represent a meaningful addition to this work, completing the STD-SCR redox cycle investigation.

Response: We thank the reviewer for this helpful suggestion. As stated in our previous response, and as the reviewer agrees, the Langmuir-type expression used in the steady-state analysis is not intended to represent the intrinsic molecularity of the Cu(I) oxidation half-cycle; rather, it provides an empirical/macroscopic description of the apparent dependence of the overall steady-state SCR rate on O_2 pressure.

To remove any potential ambiguity for readers, we have added a clarifying paragraph in the main manuscript (page 11) explicitly distinguishing the scope and purpose of the steady-state rate expression from the transient kinetic treatment. In addition, as requested, we have incorporated the kinetic analysis of the Cu(I) oxidation (O_2-pulse) transients into the manuscript/SI (SI, page 13), explicitly demonstrating the second-order dependence of the oxidation half-cycle on Cu(I).

Comment from Reviewer-4:

In the revised manuscript, the authors have followed up on the concerns of all reviewers conscientiously. The manuscript is publishable in the current form with a small correction: Please delete the comment "We have incorporated this analysis and discussion into the revised manuscript" at the bottom of page 26 in the supporting information.

Response: We thank the reviewer for carefully reviewing the manuscript and SI. We have removed this sentence from page 26 of the SI.
